# Integrative transcriptomics reveals association of abscisic acid and lignin pathways with cassava whitefly resistance

**DOI:** 10.1186/s12870-023-04607-y

**Published:** 2023-12-20

**Authors:** Danielle G. Nye, Maria L. Irigoyen, Laura Perez-Fons, Adriana Bohorquez-Chaux, Manhoi Hur, Diana Medina-Yerena, Luis Augusto Becerra Lopez-Lavalle, Paul D. Fraser, Linda L. Walling

**Affiliations:** 1 Department of Botany and Plant Sciences, University of California, Riverside, CA 92521 USA; 2 Department of Biological Sciences, Royal Holloway University of London, Egham, UK; 3 Alliance Bioversity International and International Center for Tropical Agriculture (CIAT), Cali, Colombia; 4 Institute of Integrative Genome Biology, University of California, Riverside, CA 92521 USA; 5 Present Address: International Center of Biosaline Agriculture, Dubai, United Arab Emirates

**Keywords:** Abscisic acid, Cassava (*Manihot esculenta*), Defense, Ethylene, Hemiptera, Hormone, Jasmonic acid, Salicylic acid, Transcriptome, Whitefly (*Aleurotrachelus socialis*)

## Abstract

**Background:**

Whiteflies are a global threat to crop yields, including the African subsistence crop cassava (*Manihot esculenta*). Outbreaks of superabundant whitefly populations throughout Eastern and Central Africa in recent years have dramatically increased the pressures of whitefly feeding and virus transmission on cassava. Whitefly-transmitted viral diseases threaten the food security of hundreds of millions of African farmers, highlighting the need for developing and deploying whitefly-resistant cassava. However, plant resistance to whiteflies remains largely poorly characterized at the genetic and molecular levels. Knowledge of cassava-defense programs also remains incomplete, limiting characterization of whitefly-resistance mechanisms. To better understand the genetic basis of whitefly resistance in cassava, we define the defense hormone- and *Aleurotrachelus socialis* (whitefly)-responsive transcriptome of whitefly-susceptible (COL2246) and whitefly-resistant (ECU72) cassava using RNA-seq. For broader comparison, hormone-responsive transcriptomes of *Arabidopsis thaliana* were also generated.

**Results:**

Whitefly infestation, salicylic acid (SA), jasmonic acid (JA), ethylene (ET), and abscisic acid (ABA) transcriptome responses of ECU72 and COL2246 were defined and analyzed. Strikingly, SA responses were largely reciprocal between the two cassava genotypes and we suggest candidate regulators. While susceptibility was associated with SA in COL2246, resistance to whitefly in ECU72 was associated with ABA, with SA-ABA antagonism observed. This was evidenced by expression of genes within the SA and ABA pathways and hormone levels during *A. socialis* infestation. Gene-enrichment analyses of whitefly- and hormone-responsive genes suggest the importance of fast-acting cell wall defenses (e.g., elicitor recognition, lignin biosynthesis) during early infestation stages in whitefly-resistant ECU72. A surge of ineffective immune and SA responses characterized the whitefly-susceptible COL2246’s response to late-stage nymphs. Lastly, in comparison with the model plant Arabidopsis, cassava’s hormone-responsive genes showed striking divergence in expression.

**Conclusions:**

This study provides the first characterization of cassava’s global transcriptome responses to whitefly infestation and defense hormone treatment. Our analyses of ECU72 and COL2246 uncovered possible whitefly resistance/susceptibility mechanisms in cassava. Comparative analysis of cassava and Arabidopsis demonstrated that defense programs in Arabidopsis may not always mirror those in crop species. More broadly, our hormone-responsive transcriptomes will also provide a baseline for the cassava community to better understand global responses to other yield-limiting pests/pathogens.

**Supplementary Information:**

The online version contains supplementary material available at 10.1186/s12870-023-04607-y.

## Background

Cassava (*Manihot esculenta*) is a hardy tuber crop that feeds 800 million people in over 100 countries worldwide, including small shareholder farmers of sub-Saharan Africa, who rely on the crop for subsistence [1]. However, since the 1990’s, superabundant whitefly (*Bemisia tabaci*) populations have devastated cassava yields in Africa [2, 3]. As phloem-feeders, whiteflies deplete photosynthates, deposit mold growth-promoting honeydew and transmit viral diseases, together slowing cassava growth and root production [4, 5]. In South America, the whitefly *Aleurotrachelus socialis* also significantly reduces cassava yields (60–80%) [6, 7]. A potent whitefly resistance was discovered in the Ecuadorian genotype ECU72, manifesting as nymph mortality, which prevents adult emergence and blocks population expansion [8, 9]. ECU72’s resistance also extends to four other whitefly species, providing a promising control strategy [10–12]. A better understanding of the genetic basis of this resistance will inform breeding efforts to select for identified resistance traits in African cassava.

At the genetic/molecular level, plant defense against biotic stressors begins with recognition of the attacker [13]. Generic molecular signatures derived from pathogens (pathogen-associated molecular patterns, PAMPs), insect herbivores (herbivore-associated molecular patterns, HAMPs), or from ‘debris’ from damaged host cells (damage-associated molecular patterns, DAMPs) are recognized by extracellular receptors to elicit a defense response via defense signals [14, 15]. Such signals are numerous and include the two major defense hormones SA (salicylic acid) and JA (jasmonic acid), as well as emerging defense signals ethylene (ET), abscisic acid (ABA) and reactive oxygen species (ROS) among others. Multiple signals are often necessary for basal immunity and resistance [16].

While whiteflies affect the yields of numerous crops [17], strong, fast-acting resistance specific to whiteflies has only been identified in cassava and alfalfa resulting in reduced egg deposition or mortality of early-stage nymphs [8, 18, 19]. Broad spectrum to moderate resistance to whiteflies has also been found in Solanaceous crops and their wild relatives among others [20–22]. Previous studies have provided some insight into the plant-defense signals elicited during responses to whitefly infestation. In whitefly-resistant lines of tomato, cotton, and cabbage, SA, JA/ET and ABA responses, respectively, are primarily elicited by whiteflies [23–25]. In whitefly-susceptible plant species, SA levels increase and SA or JA/ET signaling can increase [26–28]. In addition, basal resistance to whiteflies can also be promoted by JA or ABA [29–31]. To date, the molecular mechanisms underlying resistance remain largely uncharacterized. Our previous work showed that during infestation, whitefly-susceptible cassava genotypes elicit *Pathogenesis-related* (*PR*) genes involved in cell wall processes [32]. A complementary metabolomics study, which assessed cell wall phenolics in cassava genotypes ECU72 and COL2246 suggested constitutive whitefly-resistance in ECU72 to involve cell wall reinforcement [33].

To obtain a more global understanding of resistant and susceptible responses of cassava to whiteflies at the transcript level, we define the transcriptomes of whitefly-resistant ECU72 and whitefly-susceptible COL2246 in response to *A. socialis* infestation, SA, JA, ET, and ABA. Hormone-responsive transcriptomes in cassava were analyzed at the genome-scale and as subsets of defense-hormone-pathway genes. These data sets were compared to Arabidopsis and also integrated with whitefly-responsive transcriptomes to reveal the genotype-dependent biological processes occurring during infestation. Together, our integrative transcriptomics approach, supported by metabolomics analyses, identified an association of higher ABA levels and induction of ABA- and lignin-pathway and cell-wall-related genes with whitefly-resistance in ECU72. Additionally, higher SA levels and induction of SA-pathway genes were associated with whitefly-susceptibility in COL2246.

## Results

### A fast transcript-level response to SA revealed in whitefly-resistant cassava

**Fig. 1:**
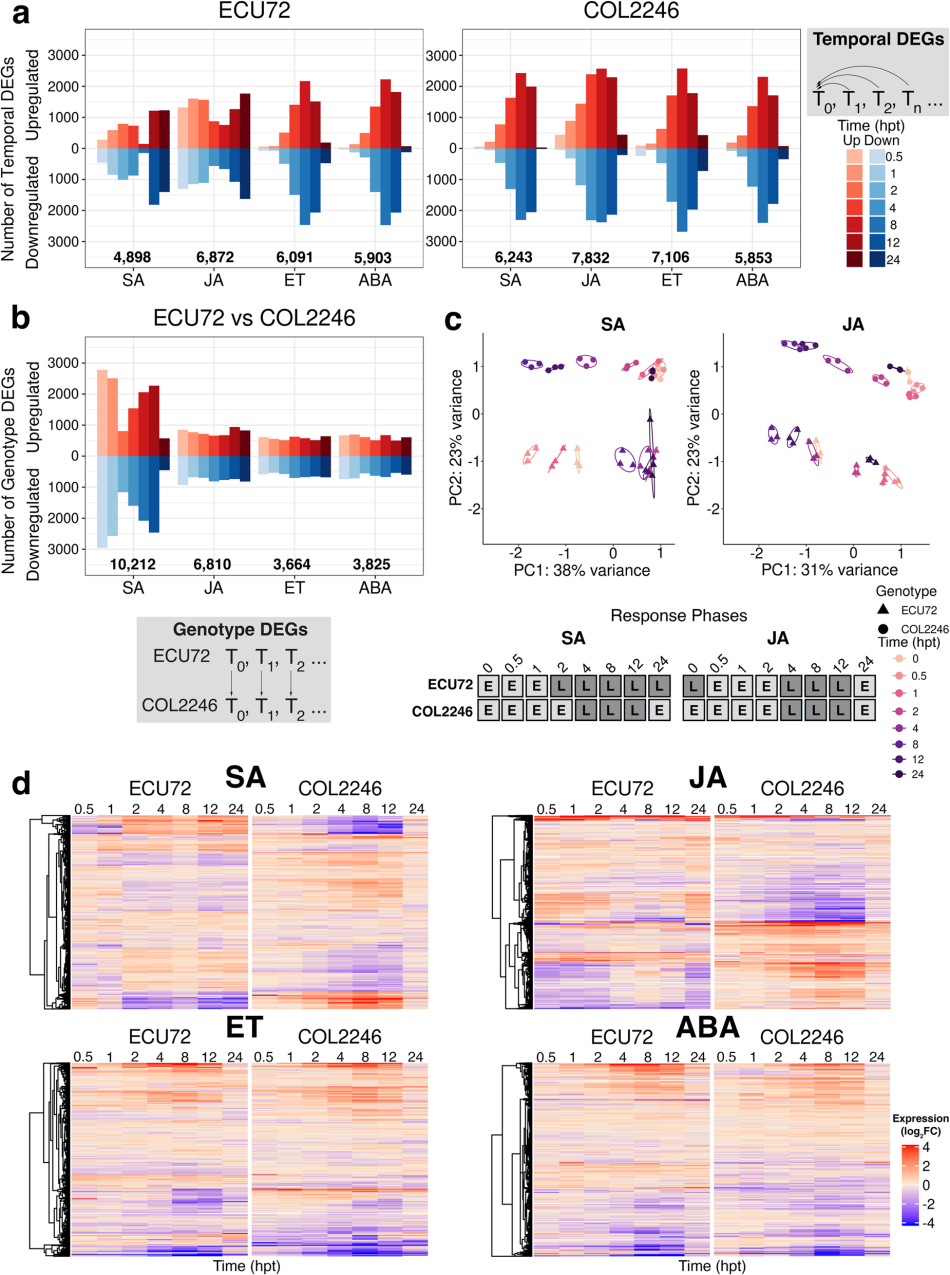
Cassava transcriptome profiles in response to defense hormone treatments. **a**, **b** tDEG and gDEG counts in ECU72 and COL2246 during SA and JA treatments (0.5, 1, 2, 4, 8, 12, and 24 hpt). A visual definition of tDEGs and gDEGs is provided. Number of up- and down-regulated genes (red and blue, respectively) are displayed. Total number of DEGs for each treatment are provided. **c** PCAs of detected gene reads prior to and after SA and JA treatments (0, 0.5, 1, 2, 4, 8, 12, 24 hpt) in ECU72 and COL2246. Clustering of time points defined distinct early (E) and late (L) response phases in ECU72 versus COL2246 for which a visualization is provided. ECU72’s early phase (0–1 hpt) samples were more similar to COL2246’s late phase (4–12 hpt) and vice versa. Normalized read count values for three biological replicates are shown per time point. Time points and genotypes are labeled by color and shape, respectively. **d** Heatmaps displaying gDEGs in ECU72 and COL2246 in response to SA, JA, ET, and ABA treatments. Expression is displayed as log_2_FC values relative to 0 hpt. DEG expression values are provided in Additional file 1 and DEG counts in Additional file 2. DEGs had |log_2_FC|≥ 1 and FDR ≤ 5%

To define the defense hormone-responsive transcriptomes of cassava, we profiled the response of cassava genotypes ECU72 (whitefly-resistant) and COL2246 (whitefly-susceptible) to SA, JA, ET, and ABA at seven times post treatment (hpt) (Fig. 1). Differentially expressed genes (DEGs) were identified by temporal comparisons within a genotype (“temporal” DEGs, tDEGs) (Fig. 1a). The number of tDEGs varied substantially by hormone (Fig. 1a; Additional file 1: Tables S1-S8). While the responses to ET or ABA were similar, ECU72 and COL2246 had distinct temporal responses to SA and JA. Notably, as early as 0.5 to 1 h post SA or JA treatment, 5- to 65-fold more tDEGs were induced and repressed in ECU72 versus COL2246 (Fig. 1a; Additional file 2: Table S13). Comparison of ECU72 and COL2246 transcriptomes at each time point identified “genotype” DEGs (gDEGs) (Fig. 1b; Additional file 1: Tables S9-S12). SA elicited 1.5–2.8-fold more gDEGs than any other hormone treatment and had a bimodal temporal program of gene expression with gDEGs peaking at 0.5 and 12 hpt (Fig. 1b; Additional file 2: Table S14).

Principal component analyses (PCAs) performed with reads detected in the SA, JA, ET, and ABA time courses revealed that while all responses had a temporal component, only SA and JA responses showed a clear distinction between genotypes (Fig. 1c; Additional file 3: Figure S1). PCAs resolved the two temporal phases, here called early and late. All COL2246 hormone responses followed the same early (0–2 hpt) and late (4–12 hpt) phases with a return to basal expression at 24 hpt. In contrast, ECU72’s late phase of SA responses initiated earlier and lasted longer (2–24 hpt). Furthermore, PCA and heatmaps of temporal gene expression showed reciprocity in ECU72’s and COL2246’s responses to SA (Fig. 1c,d). Additionally ECU72’s late phase clustered with the 0-h time point indicating a significant difference in constitutive expression of JA-responsive genes between the genotypes (Fig. 1c).

Pearson correlation analyses using genes detected during hormone treatments highlighted additional interactions between the hormone pathways in ECU72 and COL2246 (Additional file 4: Figure S2; Additional file 5: Table S15). Most striking, negative correlation values suggested antagonism between ECU72’s late SA and early JA responses with ECU72’s responses to ET and ABA. In contrast, most all other hormone responses were positively correlated between genotypes, suggesting gene co-regulation (Additional file 4).

### Reciprocal transcriptome responses to SA in cassava genotypes

The temporal patterns of gDEG expression in ECU72 and COL2246 following SA, JA, ET, and ABA treatments were visualized using heatmaps (Fig. 1d). Strikingly, most SA- or JA-responsive gDEGs were reciprocally regulated. In contrast, most ET- and ABA-responsive gDEGs had similar expression trends in ECU72 and COL2246 (Fig. 1d). To uncover the biological processes associated with hormone responses between the genotypes, k-means clustering and functional enrichment analyses of gDEGs were performed (Additional file 6: Figures S3-S6; Additional file 7: Tables S16-S19). In response to all hormone treatments, the GO term categories response to hormone, stimulus or biotic stimulus were enriched, confirming the effectiveness of hormone treatments in eliciting hormone responses (Additional file 6: Figures S3-S6). Clustering also revealed that differential response between the genotypes was attributed to reciprocal regulation for SA gDEGs, but was due to differing 0-h transcript levels for JA gDEGs (Additional file 6: Figures S3-S4).

### Cassava and Arabidopsis hormone responses have diverged

To place cassava’s hormone responses within a broader context, the SA- and JA-dependent transcriptomes of cassava were compared to those of *Arabidopsis thaliana*. Previous microarray or RNA-seq studies profiling SA and JA responses in Arabidopsis utilized various hormone concentrations and plants of different ages resulting in marked differences in DEGs identified in each study (Additional file 8: Figure S7a,b; Additional file 9: Tables S20 and S21). Therefore, RNA-seq analyses of SA- and JA- treatment time courses (0, 0.5, 1, 2, 4, 8, 12, and 24 hpt) in Arabidopsis were performed for comparison to cassava time courses (Additional file 10: Figure S8a; Additional file 11: Tables S22 and S23; Additional file 12: Table S24). Of the Arabidopsis SA and JA tDEGs we identified, over 20% were identified by previous transcriptome studies (Additional file 8: Figure S7c,d).

**Fig. 2:**
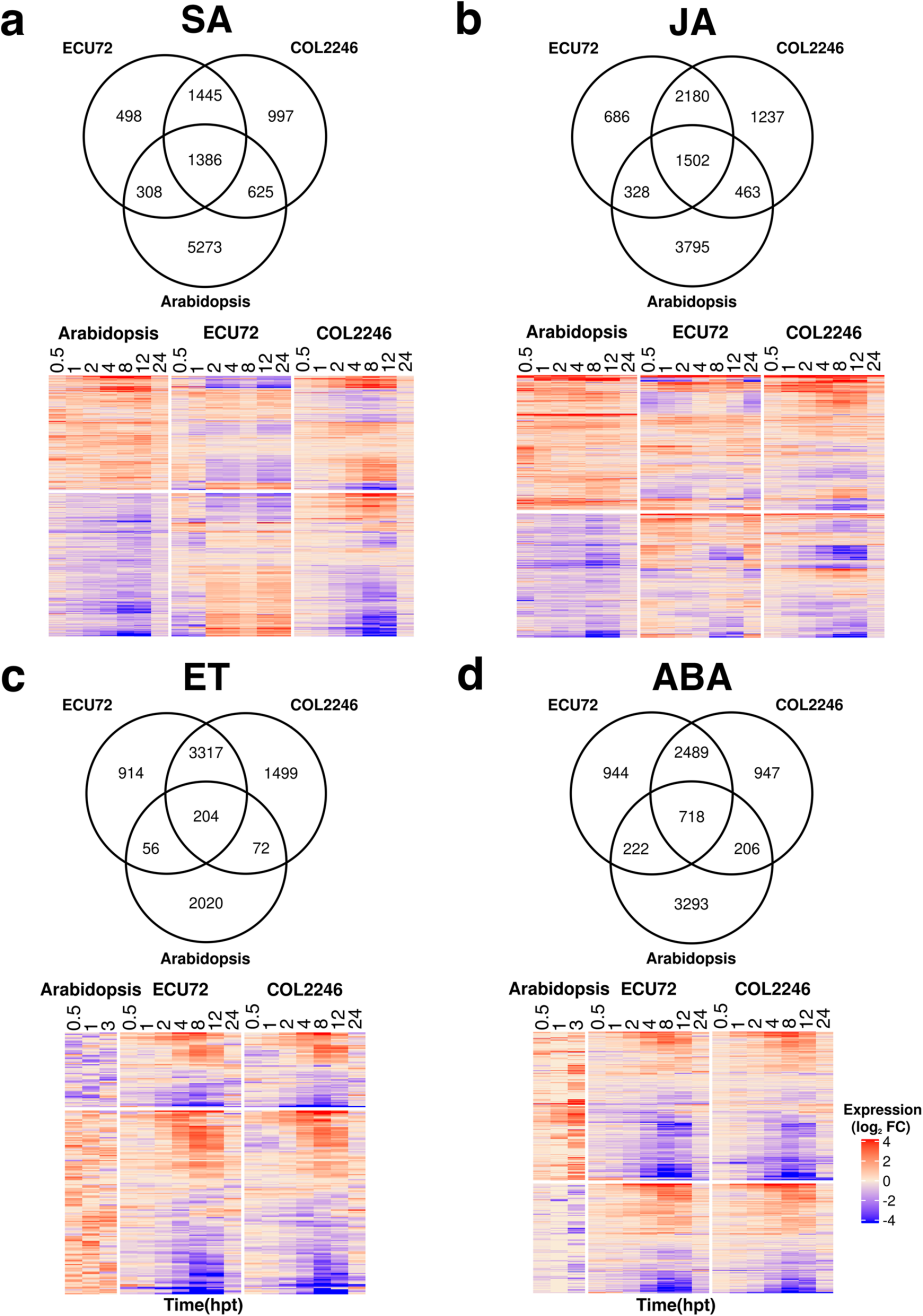
Comparison of Arabidopsis and cassava hormone responses. **a**-**d** Venn diagrams and heatmaps comparing tDEGs in Arabidopsis and cassava (COL2246 and ECU72) identified during SA, JA, ET, and ABA treatments, respectively. Core genes shared between Arabidopsis and both cassava genotypes are visualized in heatmaps. Arabidopsis SA and JA tDEGs were identified in this study. Arabidopsis ET- and ABA-responsive tDEGs at 0.5, 1 and 3 hpt were retrieved from Goda et al. [34]. Expression values are provided in Additional files 11 and 13

PCA analyses indicated that the Arabidopsis response to SA and JA was separated into two phases similar to cassava. However, Arabidopsis’ response timing was more similar to COL2246, as ECU72’s biphasic response to SA and JA was not observed (Fig. 1c; Additional file 10: Figure S8b). In comparing SA-, JA-, ET-, or ABA-responsive genes in Arabidopsis and cassava, strikingly different regulatory programs between the species were revealed [34] (Fig. 2; Additional file 11; Additional file 13: Tables S25 and S26). Of the phytohormone-responsive genes in Arabidopsis, only a small core were responsive to the same phytohormone in both cassava genotypes. For these core genes, Arabidopsis’ regulatory programs did not always align with cassava responses. For example, many hormone up-regulated genes in Arabidopsis were down-regulated in ECU72 or COL2246 (Fig. 2).

Given the marked differences in the transcriptome responses of Arabidopsis and cassava to the defense hormones, we determined the expression programs of 162 Arabidopsis genes central to SA, JA, ET, and ABA biosynthesis, modification, transport, and signaling (encompassing perception, signaling and response) and their orthologs in cassava [35–38] (Additional file 14: Figures S9-S12; Additional file 15: Tables S27-S30). Over 40% of Arabidopsis hormone-pathway genes had multiple orthologs in cassava, with many displaying diverged expression programs (Additional file 16: Table S31).

The species differences were most apparent in the SA pathway (Additional file 14: Figure S9). Many SA-pathway genes in Arabidopsis had regulatory trends similar to one cassava genotype and displayed reciprocal regulation between the cassava genotypes. Several of these genes (*MeNPR1*, *MeWRKY70a-b*, *MeGRX480c*, *MeCBP60a1*, and *MeSARD1a*) shift from up- to down-regulation or vice versa from the early to late SA response phases in ECU72, suggesting they may act as transcriptional regulators for this phase shift (Additional file 14: Figure S9). Similarly, the early responses of ET- and ABA-pathway genes were substantially different between the two species. For instance, the ET-induced, single-copy gene *AtPR3* had 15 orthologs in cassava, 13 of which were undetected or repressed after ET treatments [32] (Additional file 14: Figures S10 and S11). In contrast, the JA-pathway genes of Arabidopsis, ECU72 and COL2246 had overall similar responses to JA. A notably lower or no induction of *MeVSP* and several *MeLOX2* genes was however observed in COL2246 (Additional file 14: Figure S12).

### Genotypic differences in cassava’s responses to whitefly infestation

**Fig. 3:**
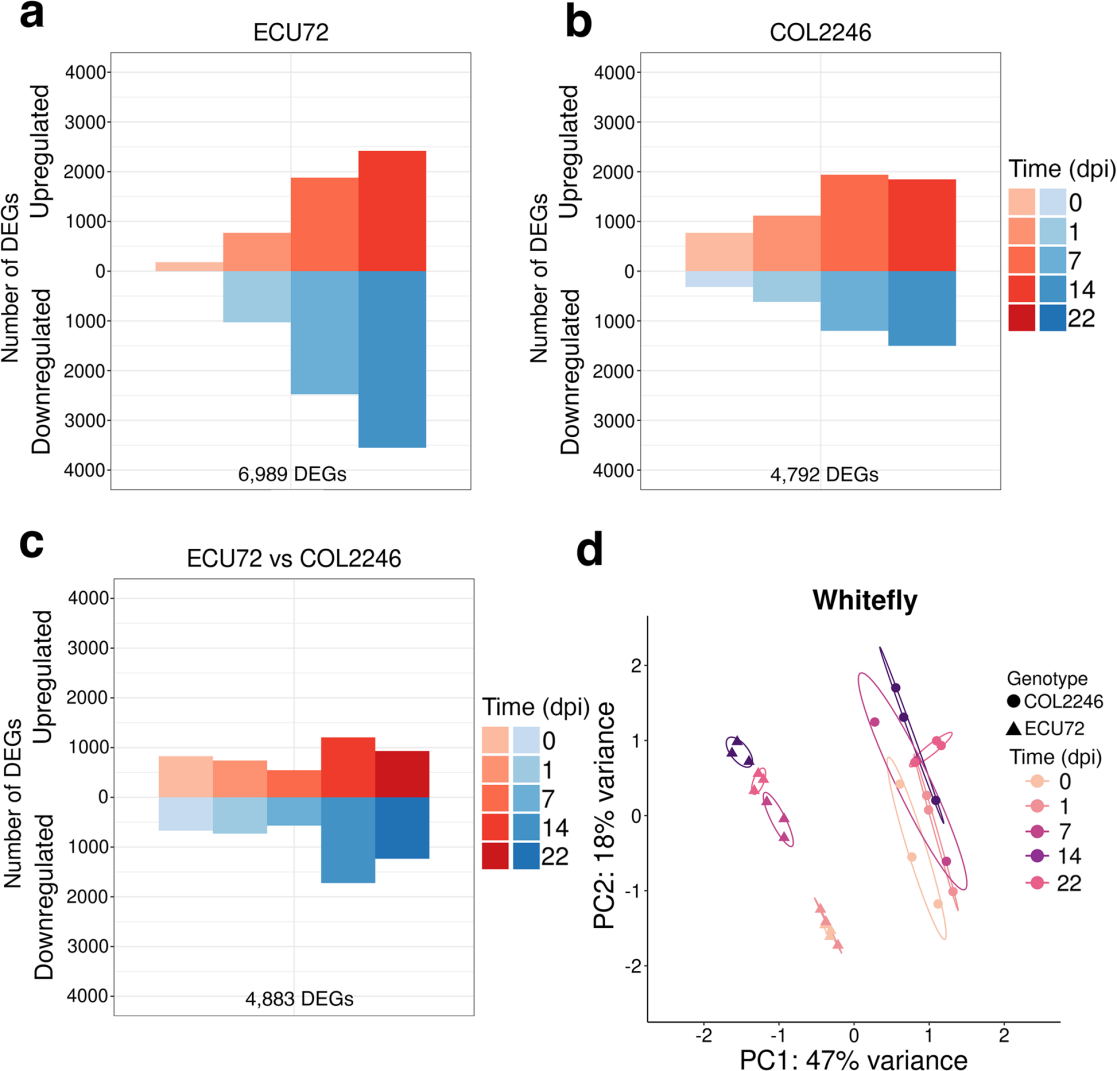
Transcriptome profiles and PCA of cassava’s whitefly infestation response. **a**-**c** Numbers of tDEGs in ECU72 and COL2246 and gDEGs during whitefly infestation. Number of up- and down-regulated genes (red and blue, respectively) are displayed and total number of DEGs for each treatment are provided. DEG expression values are provided in Additional file 17 and DEG counts in Additional file 18. DEGs had |log_2_FC|≥ 1 and FDR ≤ 5%. **d** PCA analyses of detected genes prior to and during whitefly infestation (1, 7, 14 and 22 dpi) in ECU72 and COL2246. More variation among biological replicates was observed in COL2246 than in ECU72. Normalized RNA-seq read count values for three biological replicates are shown per time point. Time points and genotypes are labeled by color and shape, respectively

To define cassava’s response to whiteflies, we analyzed the transcriptomes of whitefly-resistant ECU72 and whitefly-susceptible COL2246 at 0, 1 (adults/eggs), 7 (eggs), 14 (1^st^ instars), and 22 (2^nd^-3^rd^ instars) days post infestation (dpi) with the Latin American whitefly *A. socialis* (Fig. 3; Additional file 17: Tables S32 and S33)*.* The temporal expression of whitefly tDEGs in ECU72 and COL2246 were distinct. At 1 dpi, when adults are feeding and eggs are deposited, COL2246 had over fivefold more tDEGs than ECU72. However, by 14 to 22 dpi, when nymphs are feeding, the magnitude of ECU72’s response exceeded that of COL2246; in particular, ECU72 repressed over twofold more genes than COL2246 at these later times (Fig. 3a,b; Additional file 18: Table S35). Analysis of gDEGs also indicated that ECU72 and COL2246 had different transcriptomes prior to and during adult feeding and oviposition (0–1 dpi), with more pronounced differences after initiation of nymph feeding 14–22 dpi (Fig. 3c; Additional file 17: Table S34; Additional file 18: Table S36). PCA analyses further revealed that while ECU72 displayed a distinct constitutive/early (0 and 1 dpi) and late (7–22 dpi) infestation response phase, COL2246 had no clear delineation of phases (Fig. 3d).

To identify biological processes associated with whitefly infestation gDEGs, k-means clustering and GO-term enrichment analyses were performed (Additional file 19: Figure S13; Additional file 20: Table S37). Of note, Cluster 2 gDEGs were enriched for GO-term categories related to cell wall processes, and displayed higher transcript levels in ECU72 versus COL2246 at 0–1 dpi followed by a steep decline by 7 dpi. All other clusters were enriched for GO-term categories related to defense (Additional file 19).

### Whitefly infestation of cassava triggers phytohormone-dependent and -independent gene expression

**Fig. 4:**
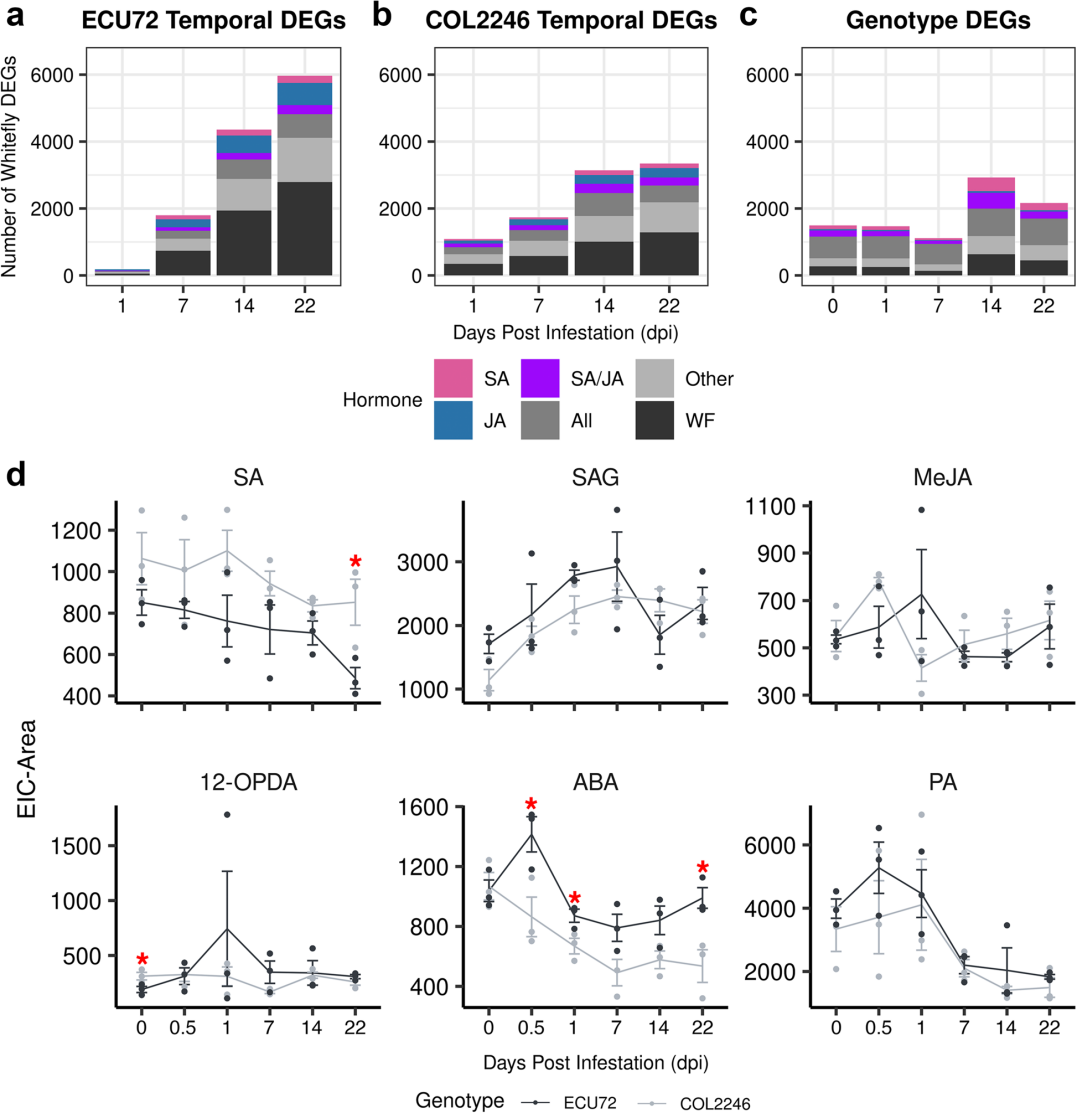
Hormone regulation of whitefly-regulated DEGs and hormone levels in whitefly-infested cassava. **a**-**c** Stacked bar graphs displaying number of whitefly-regulated tDEGs in ECU72 (**a**), tDEGs in COL2246 (**b**) and gDEGs (**c**) categorized by their hormone-response class. Hormone-response categories included responses to single or multiple defense hormones. Hormone categories that contributed to more than 10% of the whitefly-infestation response are shown. The hormone-response category “All” reflects the ability of a DEG to independently respond to SA, JA, ET, and ABA. The “WF” category indicates that DEGs responded to whitefly infestation but none of the defense hormones tested. The hormone category “Other” includes all single or multiple hormone-response categories that constitute less than 10% of whitefly-responsive genes at a time point. Gene counts by hormone category are provided in Additional file 21. **d** Defense hormones detected during infestation in ECU72 and COL2246. JA and JA-Ile were not detected and no significant changes in MeJA levels were observed during whitefly infestation of ECU72 or COL2246. Asterisks (red) indicate significant difference in hormone level between genotypes as identified by Student’s t-test (* = *p* ≤ 0.05). Complete lists of detected hormones and *p*-values is provided in Additional file 22 including SA, SAG (SA glucoside), MeJA (methyl jasmonate), 12-OPDA(12-oxo-phytodienoic acid), ABA, and PA (phaseic acid)

To gain a global understanding of the association of hormone-responsive genes with whitefly infestation, we classified ECU72’s and COL2246’s whitefly-regulated DEGs by their responsiveness to a single or multiple defense hormones (Fig. 4a-c; Additional file 21: Tables S38 and S39). Most ECU72 and COL2246 tDEGs (29–47%) responded to signals independent of SA, JA, ET, and/or ABA.The tDEGs regulated by all four hormones (12–22%) made up the next largest hormone-response class. Many of the remaining whitefly-regulated tDEGs were responsive to SA, JA or both SA and JA (Fig. 4a,b). In addition, ABA appears to be an important regulator in ECU72’s response to whitefly infestation at 14 to 22 dpi, as the number of ABA-responsive genes (in the “Other” category) was over twofold higher in ECU72 than in COL2246 (Fig. 4a,b; Additional file 21: Table S38). In contrast to tDEGs, most whitefly-responsive gDEGs were responsive to SA (5.2–14.0%) or all hormones (28.1–54.6%) at all infestation times (Fig. 4c; Additional file 21: Table S39).

The levels of SA, JA, ABA, and their derivatives during whitefly infestation (0, 0.5, 1, 7, 14, and 22 dpi) in ECU72 and COL2246 were extracted from an untargeted metabolomics data set [33] (Fig. 4d; Additional file 22: Tables S40 and S41). The JA precursor 12-OPDA (12-oxo-phytodienoic acid) was significantly higher in COL2246 at 0 dpi. As OPDA regulates defense genes in a JA-independent and -dependent manner [39–41], 12-OPDA may mediate constitutive differences in gene expression between the genotypes. SA levels were higher at all times in COL2246 versus ECU72, reaching statistical significance at 22 dpi. Reciprocally, the levels of SAG (SA glucoside, SA’s storage form) demonstrated a trend of higher levels in ECU72 from 0–7 dpi. In contrast, ABA levels were higher in ECU72 with statistical significance at 0.5, 1, and 22 dpi. The highest ABA levels were reached at 0.5 dpi, preceding transcripts changes by 12 h. In addition, the levels of the oxidation metabolite of ABA phaseic acid (PA) followed trends similar to ABA (Fig. 4d). Correlation analyses additionally showed that changes in hormone levels were associated with changes in transcript levels in ECU72 and COL2246 during whitefly infestation (Additional file 23: Figure S14c,d).

To determine the possible involvement of hormone-pathway genes in infestation, the hormone and whitefly responses of defense-hormone pathway genes that were whitefly gDEGs were visualized by heatmaps (Additional file 24: Figure S15; Additional file 15). Many genes that were whitefly-upregulated in both genotypes and more highly expressed in COL2246 at 14–22 dpi (Cluster 1) were involved in positive regulation of SA signaling (*MeNPR1*, *MeSARD1a* and *MeWRKY70a-b*) or SA responses (*MePR1a* and *b*). In contrast, genes that were induced in ECU72 but repressed in COL2246 during infestation (Cluster 3) were predominantly involved in ABA signaling, ET signaling or SA inactivation. These ABA signaling genes are known co-receptors involved in negative feedback loops in Arabidopsis [42], suggesting their whitefly induction may be involved in fine-tuning ECU72’s ABA response.

### Resistant cassava deploy multiple biochemical defenses in response to whitefly adult feeding and egg deposition

**Fig. 5:**
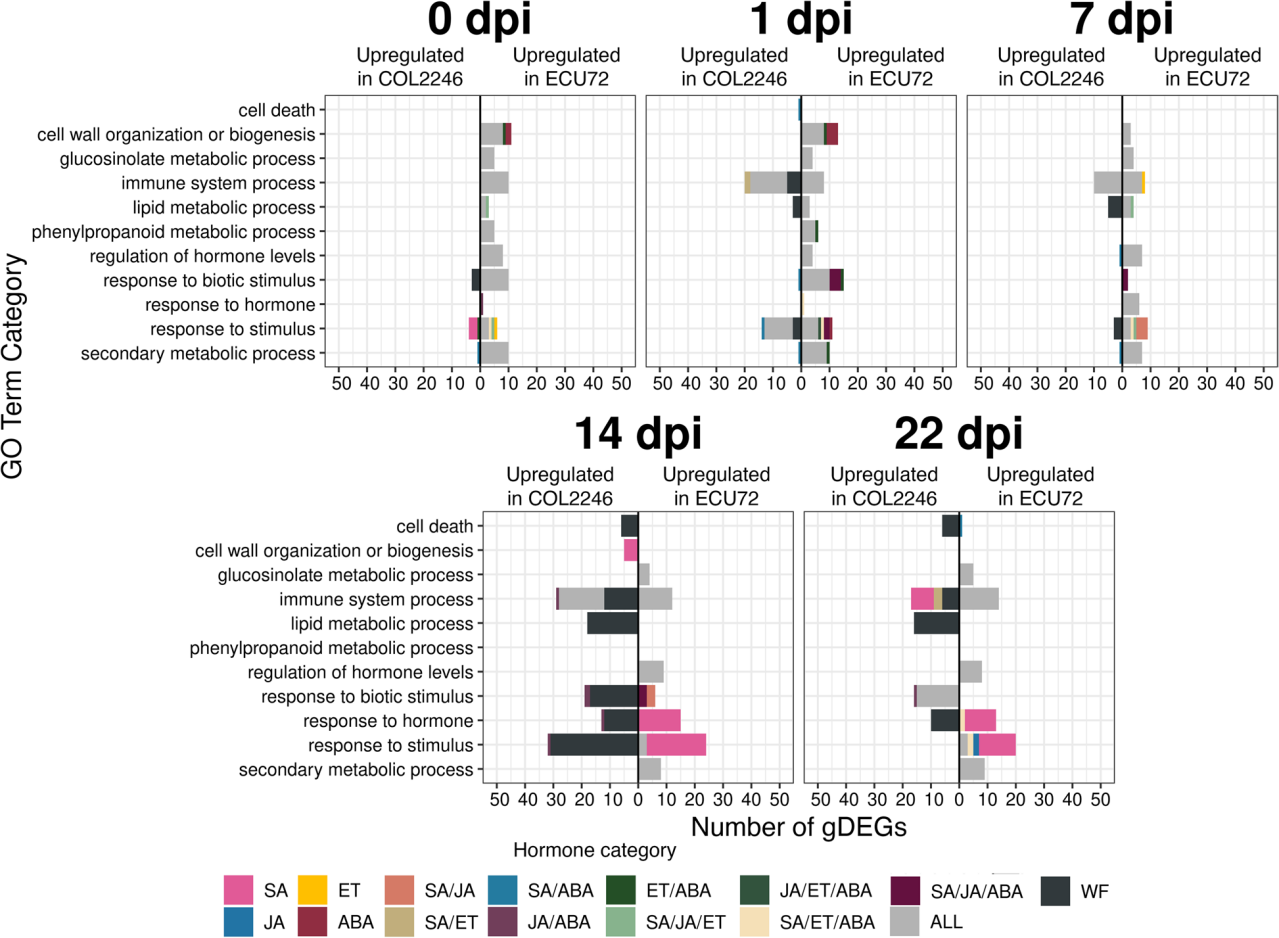
Functional enrichment of cassava gDEGs in response to whitefly and hormone treatments. GO-term enrichment was performed on whitefly gDEGs. Numbers of genes enriched for terms linked to defense are shown. gDEGs responsive to one-three hormones, all hormones, or that are hormone-nonresponsive (WF) are shown. Genes upregulated in ECU72 or COL2246 are displayed on the right and left sides of the x-axis, respectively. Counts and identities of genes within each GO term category are provided in Additional file 25: Table S42 and Additional file 26: Table S44

To assign biological functions to the gDEGs identified in both whitefly and hormone treatments, GO term-enrichment analyses were performed (Additional file 25: Tables S42 and S43; Additional file 26: Tables S44 and S45). gDEGs were grouped by infestation time point, hormone responses and up- or down-regulation in ECU72 versus COL2246 during whitefly infestation. Enrichment results are shown as categories in defense (Fig. 5) or other processes (Additional file 27: Figure S16). Among the defense-associated gDEGs that were up-regulated in ECU72 prior to (0 dpi) or early after infestation (1–7 dpi), 81% responded to all four hormones and were associated with GO terms including cell wall-related and immune system processes, glucosinolate, phenylpropanoid and lignin metabolism, and hormone/stimulus responses (Fig. 5).

Visualizing the RPKM expression trends for 23 genes within these enriched GO-term categories showcased associations between infestation and hormone regulation (Additional file 28: Figure S17). Genes involved in cell wall remodeling (*MeXTH23*, *MeGUX1* and *MePME31*), cell wall biosynthesis (*MeCESA4*), response to fungal cell wall elicitors (*MeCAD8i*) [43], and lignin biosynthesis (*MeCOMTf*, *MeCCOAMTa*, *MeMYB63*, *MeLAC4*, and *MeLAC5*) were more highly expressed in ECU72 versus COL2246 prior to infestation (0 dpi) and at most times during infestation and hormone treatments. Seven immunity genes displayed similar expression trends (Additional file 28).

As four glucosinolate metabolism genes (*MeSOT17*, *MeCYP83B1a*-*c*) [44] were more highly expressed in ECU72 in all treatments (Additional file 28), we speculated that glucosinolate levels may be altered in ECU72. Previous publications report the ability of exogenously expressed cassava enzymes to produce glucosinolates [45, 46]. Therefore, given that *MeCYP83B1a*-*c* were induced in whitefly-infested ECU72, and Arabidopsis AtCYP83A1 and AtCYP83B1 catalyze the conversion of valine, isoleucine and phenylalanine aldoximes into their corresponding glucosinolates, we measured their levels in cassava leaves [44]. Desulfonated glucosinolates, the thioglucose moieties characteristic of glucosinolate structures, or molecular ions corresponding to glucosinolates derived from either valine, isoleucine or phenylalanine were not detected in cassava leaves (Additional file 29: Figure S18). Our results suggest that glucosinolates may be at undetectable levels or that cassava’s *MeCYP83B1* genes are acting in alterative pathways in infested leaves.

**Fig. 6:**
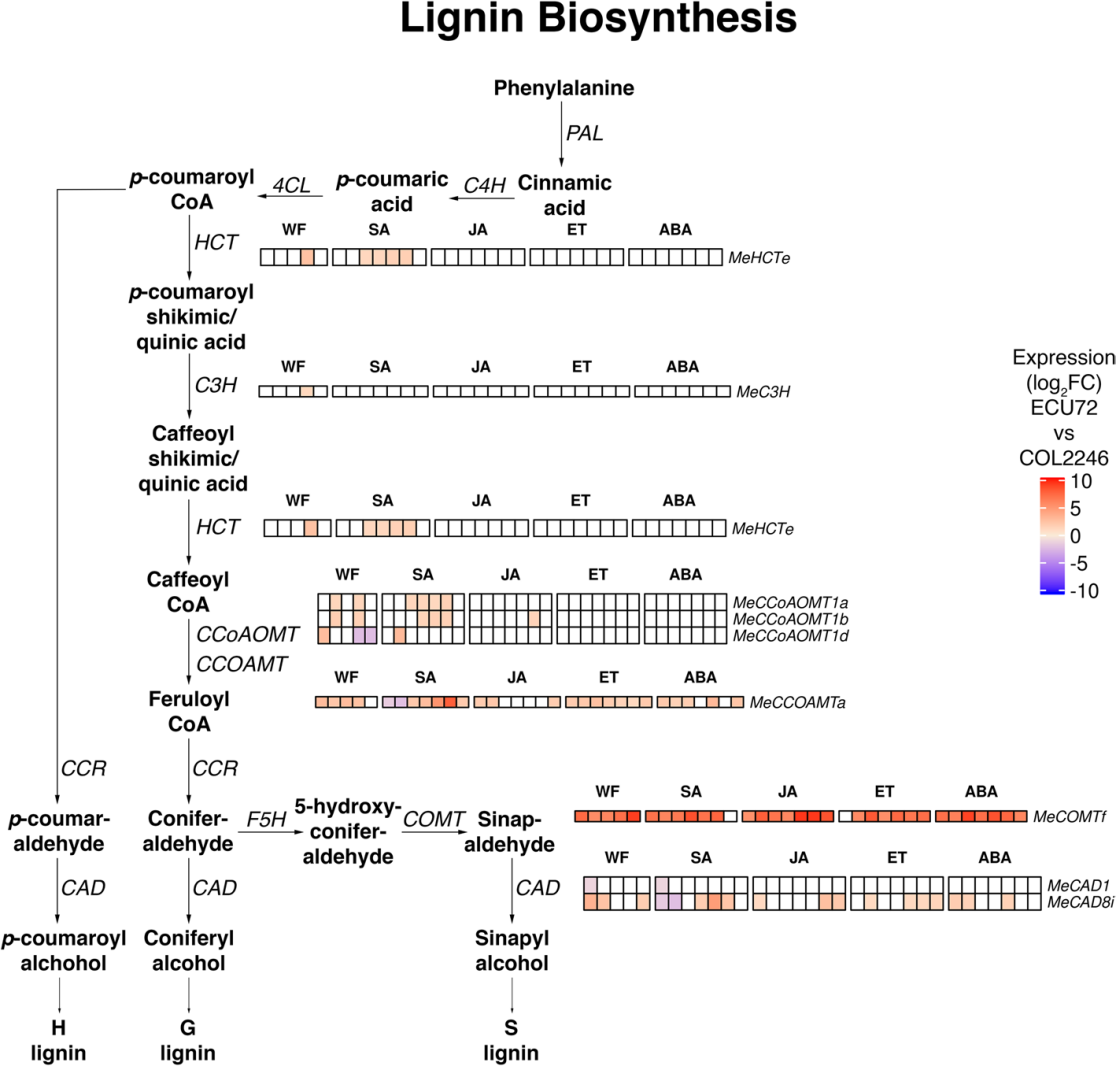
Cassava’s lignin biosynthetic pathway. Expression of whitefly-infestation gDEGs during whitefly and SA, JA, ABA, and ET treatments are shown beside lignin-pathway genes. Of note, *MeCOMTf* and *MeCCOAMTa* showed strong upregulation in ECU72 versus COL2246 at most infestation and hormone-treatment time points. Expression is displayed as log_2_FC values in ECU72 as compared to COL2246 with ECU72 upregulated (red) and downregulated (blue) gDEGs displayed. Time points where gene expression was not significantly different between the genotypes are shown in white. Time points from left to right during infestation are 0, 1, 7, 14, and 22 dpi and during hormone treatments are 0.5, 1, 2, 4, 8, 12, and 24 hpt. Gene loci are provided in Additional file 30

As several genes involved in lignin biosynthesis were identified as up-regulated gDEGs in the whitefly-resistant ECU72 (Additional file 28), we identified the set of cassava orthologs associated with this metabolic pathway that were whitefly gDEGs (Fig. 6; Additional file 30: Table S46). Among these ten genes, eight were more highly expressed in ECU72 during infestation (Fig. 6).

### Whitefly nymph feeding induces a surge in SA and immune signaling in susceptible cassava

A major shift occurred at the time of nymph feeding (14–22 dpi) when many defense-associated GO terms were enriched among a surge in genes upregulated by infestation in COL2246 versus ECU72. Genes within these GO-term categories were mainly regulated by all hormones or were defense-hormone independent (Fig. 5). Visualization of 24 individual gene transcript levels exemplified such expression trends (Additional file 31: Figure S19). For instance, within the immune system process category, *MePR3a*, *MeFMO1*, NLR *MeRPP8*, and *MePERK1a-c* (receptors that perceive cell wall perturbations) were more highly expressed in COL2246 than in ECU72 during infestation and all hormone treatments. Four starch catabolism and three sesquiterpenoid biosynthetic genes also had higher expression in COL2246 versus ECU72 by late infestation. Although sesquiterpenoid production is known to be JA induced in plants, such as maize and rice [47, 48], the cassava sesquiterpenoid biosynthetic geneswere surprisingly not regulated by SA, JA, ABA, or ET (Additional file 31). Interestingly, for genes enriched in processes other than defense (i.e., metabolism, carbohydrate metabolism, transport), a similar shift in enriched terms from 0–7 versus 14–22 dpi was observed (Additional file 27; Additional file 26: Table S45).

**Fig. 7:**
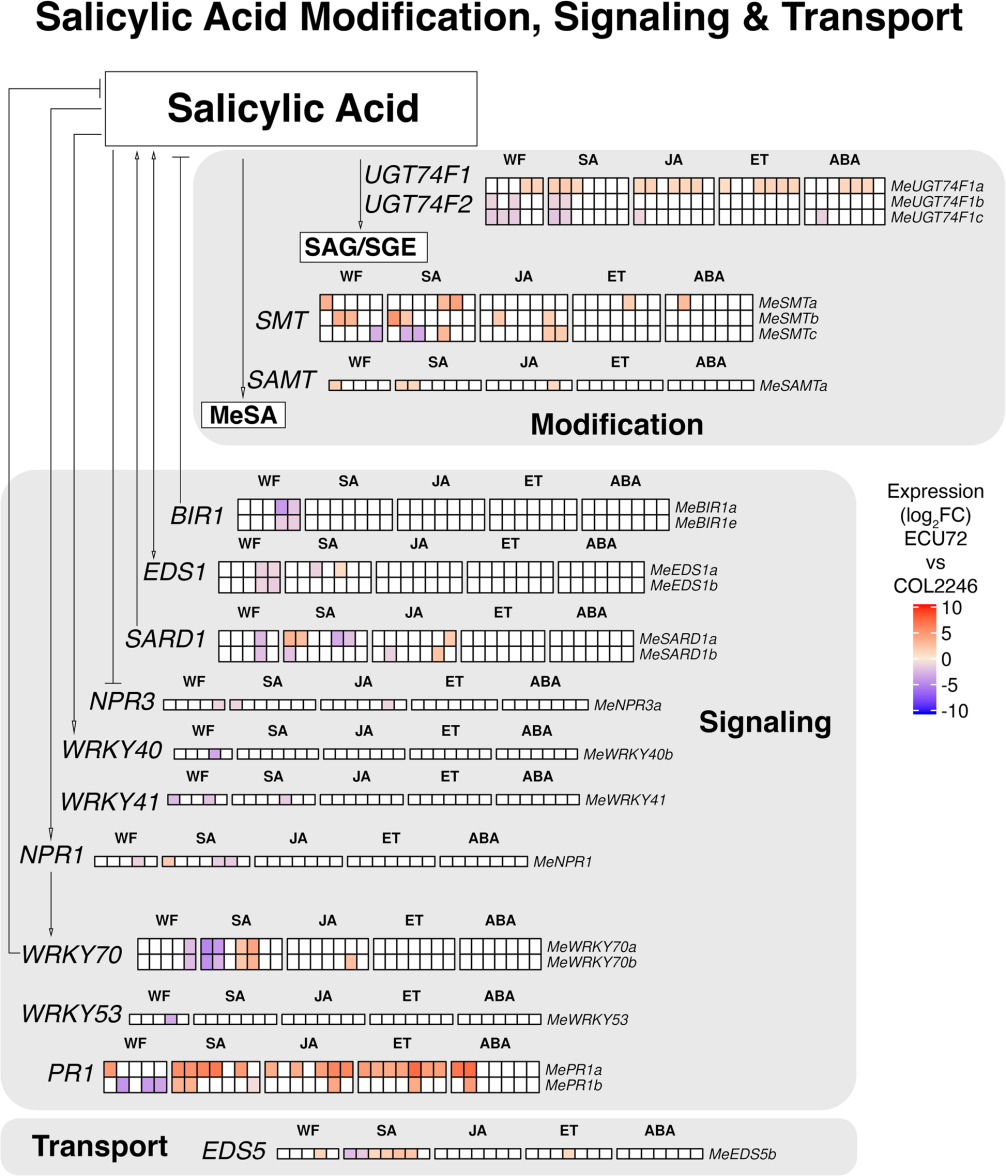
Cassava’s SA modification, signaling and transport pathway. Expression of whitefly-infestation gDEGs during whitefly and SA, JA, ABA and ET treatments are shown beside SA-pathway genes. Expression is displayed as log_2_FC values in ECU72 as compared to COL2246 with ECU72 upregulated (red) and downregulated (blue) gDEGs displayed. Time points where gene expression was not significantly different between the genotypes are shown in white. Time points from left to right during infestation are 0, 1, 7, 14, and 22 dpi and during hormone treatments are 0.5, 1, 2, 4, 8, 12, and 24 hpt. Gene loci are provided in Additional file 15: Table S27

Given the reciprocal regulation of SA-responsive genes in ECU72 and COL2246 (Fig. 1d) and detection of many SA-pathway genes in the enriched GO-term categories (Fig. 5), we examined 23 whitefly gDEGs identified in our annotation of the cassava SA pathway and/or identified in our enrichment analyses (Fig. 7; Additional file 15: Table S27). Of these genes, 15 were signaling genes, and all but one, were more highly expressed in COL2246 versus ECU72 at 14–22 dpi. Notably, four were SA-responsive gDEGs (*MeEDS1a*, *MeWRKY41*, *MeNPR1*, and *MeWRKY70a*) and four were gDEGs that responded to both SA and JA (*MeSARD1a* and *b*, *MeNPR3*, and *MeWRKY70b*). At one or more early infestation times (0-7dpi), *MeSMTa, MeSMTb* and *MeSAMTa* were more highly expressed in ECU72*;* these enzymes convert SA into the mobile signal of systemic resistance, MeSA (methyl salicylate). In contrast, two of the three *MeUGT74F1* genes, which encode for enzymes that convert SA into its storage forms SAG (SA glucoside) or SGE (SA glucose ester), were more highly expressed in COL2246 at 0–7 dpi (Fig. 7). Higher expression of these SA-modifying genes may reflect an attempt of COL2246 to modulate its high levels of free SA that occur early after infestation.

## Discussion

### Divergent hormone responses in cassava versus Arabidopsis

In comparing the hormone-responsive transcriptomes of cassava and Arabidopsis, we found significant differences in gene expression both globally and within hormone pathways (Fig. 2; Additional file 14). Additionally, the numbers of hormone-pathway genes present were often expanded or contracted between species (Additional file 16). As neofunctionalization is often observed in polyploid species [49], such expansions may suggest adapted functions in defense. For example, the *PR3* chitinase gene family is expanded in cassava, tomato and Eucalyptus relative to Arabidopsis [32, 50, 51], suggesting an adaptive response to insect/pathogen-derived chitin [52]. Finally, we found that many canonical markers of SA, JA, ET, or ABA signaling in Arabidopsis are not sentinels of phytohormone pathways in cassava, similar to some tomato *PR* genes [53]. This includes the classical Arabidopsis *PR* genes [32], as well as the JA-induced Arabidopsis marker *AtVSP1*.

### Hormone responses of whitefly-resistant and -susceptible cassava

Our comparison of ECU72’s and COL2246’s hormone levels and hormone-responsive transcriptomes revealed several key findings. While COL2246 responded similarly to all hormones, ECU72 showed evidence of antagonism between its SA and JA versus ET and ABA responses (Additional file 4). Most striking, while ECU72 and COL2246 displayed similar temporal responses to JA, ET and ABA, they showed largely reciprocal regulation by SA. In cacao, a similar reciprocity of some SA-responsive genes was seen in fungus-resistant versus -susceptible genotypes [54]. Also similar to our findings, van Leeuwen et al. [55] reported that Arabidopsis ecotypes showed variable responses to SA and Proietti et al. [56] showed variation in the degree of SA antagonism with JA in Arabidopsis accessions. These findings suggest hormone responses may vary considerably between and even within plant species.

Our finding that ECU72 displays a faster-acting and more prolonged SA response than COL2246 also suggests that a master transcriptional regulator may facilitate this phase shift. The SA-signaling genes *MeNPR1*, *MeWRKY70a-b*, *MeGRX480c*, *MeCBP60a1*, and *MeSARD1a* are possible candidates as their expression profiles change during this early to late phase shift in ECU72 (Additional file 14: Figure S9). In Arabidopsis, *AtNPR1*, *AtWRKY70* and *AtGRX480* promote SA responses important for resistance to biotrophic pathogens, while suppressing JA/ET responses important for resistance to insect herbivores (like whiteflies and caterpillars) and necrotrophic pathogens [28, 57–59]. *AtWRKY70* is also known to suppress ABA-induced stomatal closure [60]. As *MeNPR1*, *MeWRKY70* and *MeSARD1a* were more highly induced at 14 dpi in COL2246 (Fig. 7), it is tempting to speculate that one or more of these transcription factors enact an ineffectual SA response in response to whitefly infestation in COL2246. Conversely, their fine-tuned regulation in ECU72 may allow for an ABA-mediated resistance response to whitefly in ECU72.

### Resistance to whitefly adults and eggs is associated with ABA and cell wall defenses

Our integration of whitefly and hormone transcriptome and metabolite data sets highlighted the association of ABA and SA with whitefly resistance and susceptibility in ECU72 and COL2246, respectively (Fig. 4; Additional file 24). The observed association of high ABA and low SA levels in ECU72 could possibly have been achieved through SA-ABA antagonism, something that has been previously described in Arabidopsis-pathogen interactions [16]. Furthermore, ECU72’s lower levels of free SA and higher levels of SAG (SA’s vacuolar storage form of SA) at 0.5–7 dpi [61] also suggests SA modification mechanisms may be important for quelling ineffective SA responses in ECU72 during early infestation.

The role of ABA in ECU72’s fast-acting resistance phenotype is intriguing as increases in ABA is an early response to PAMPs that promotes stomatal closure to interfere with pathogen access to a leaf’s interior spaces [62]. With limited evidence for hemipteran-plant interactions, in whitefly-resistant ECU72, ABA-mediated stomatal closure could slow phloem feeding by decreasing transpiration rate, or, it could decrease the release of whitefly-attracting plant volatiles. Alternatively, ABA may promote stomatal opening to improve gas exchange for resumption of basal photosynthesis rates after initial responses to whitefly attack [63]. Several studies have shown either ABA or osmotic stress responses to be important for plant resistance to whiteflies [24, 29, 30] and other insect pests [64], however the overall role of ABA in defense against insects remains unclear.

Enrichment analyses additionally showed that during early infestation (0–7 dpi), few defenses were mounted in COL2246. In contrast, cell wall defenses like lignin biosynthesis were already active in ECU72 (Figs. 5 and 6). The importance of cell-wall based defenses against whiteflies discovered here is corroborated by our findings of higher basal and whitefly-induced leaf lignin levels in ECU72 versus COL2246 by Perez-Fons et al. [33], as well as observed cell wall-based defenses in cotton responses to whiteflies [25, 65, 66]. Our analyses additionally pointed to the importance of cell wall fortification and the sensing of cell wall damage during ECU72’s early infestation response (Additional file 28). Strengthening the cell wall by enhancing lignin biosynthesis to deter the probing of whitefly stylets and the perception of damage through cell wall elicitors/DAMPs may be important components of ECU72’s defense during early stages of infestation [67–69]. It is also interesting to note that by late infestation (14–22 dpi) even though fewer nymphs are present on ECU72, it has a higher magnitude response than COL2246. This suggests that ECU72 is able to prompt prolonged whitefly defenses even with minimal insect signals.

### Susceptibility to whitefly nymphs is associated with a rise in SA and immune signaling

A marked shift in the cassava transcriptome was observed at the times of voracious phloem feeding by first to third instar nymphs (14–22 dpi) when a surge in SA and immune responses in COL2246 was initiated (Fig. 5; Additional files 24 and 31). Per our direct observations, at this time, the susceptible plant has a heavy load of developing 2^nd^- and 3^rd^- instar nymphs. In contrast, on the resistant plant, the majority of nymphs have ceased development or perished thereby reducing the quantity of effectors/elicitors being delivered to the resistant plant; this may explain the absence of a burst in SA responses in ECU72, as was observed in COL2246. Indeed, it is known that an insect’s developmental stage influences plant responses at the transcriptome and metabolome level [32, 33, 70]. The role of SA in plant-whitefly interactions has been shown to vary based on the plant and pest species [71]. However, like COL2246, SA responses mounted during whitefly infestation do not interfere with but they promote whitefly performance on whitefly-susceptible Arabidopsis, tobacco and lima bean [26, 28, 72].

Starch catabolism and sesquiterpenoid biosynthetic genes were additionally more highly expressed in COL2246 at 14–22 dpi (Additional file 31). As observed in other plants, the breakdown of starch may be a strategy to mobilize stored energy to compensate for photosynthate depletion due to insect infestation [73]. Emission of terpenoid volatiles has also been previously found in response to aphids, whiteflies and other insects for varied purposes including attracting or deterring insects or their natural predators or signaling responses in nearby plants [63, 74–76].

Lastly, it is important to note that a large portion of whitefly-responsive genes were responsive to all or none of the hormones tested indicating that they may be controlled by other defense signals. One possibility is that reactive oxygen species (ROS) underlie the different SA levels and responses seen in ECU72 and COL2246, as ROS can regulate SA pathway crosstalk via redox-regulated transcription factors like *GRX480* and *NPR1* [77–79].

## Conclusions

Here, we provide the first global analysis of cassava’s response to whitefly infestation, as well as to the defense hormones SA, JA, ET, and ABA, in the whitefly-resistant and whitefly-susceptible cassava genotypes ECU72 and COL2246. Comparisons of hormone-dependent transcriptomes in cassava and the model plant Arabidopsis revealed a striking divergence in expression programs. We suggest that such interspecies divergence may be more common than currently understood, and caution the assumptive adoption of commonly used Arabidopsis defense sentinel genes in other plant species.

Comparisons of hormone-responsive transcriptomes and findings in the Arabidopsis literature suggested several possible SA-signaling genes that may facilitate the observed SA-ABA antagonism in ECU72, ECU72’s fast-acting SA response involving a phase shift, and the resulting largely reciprocal response of ECU72 and COL2246 to SA treatment. However, additional genetic testing in gene-edited or transgenic cassava is necessary to determine a possible role of these genes in defense against whiteflies. Such observed intraspecies variation in phytohormone responses, while understudied, may reflect fine tuning to the suites of pests, pathogens or conditions to which each genotype has adapted.

Our integrative analyses together suggest that COL2246’s late infestation response to large numbers of actively feeding nymphs occurs due to the absence of effective early control strategies and ineffective SA-mediated defenses. In contrast, ECU72’s faster response to whitefly adults and eggs via ABA-mediated responses and lignin-based cell wall defenses may underlie its resistance to whitefly infestation. A possible link between ABA and stomatal responses to whitefly in ECU72 remains unclear and requires further investigation. Additionally, as many whitefly-regulated genes were unresponsive to tested hormones, further studies are required to identify remaining unknown signals important for regulating cassava’s response to whitefly infestation.

Hormone-responsive transcriptomes generated in this study will serve as a valuable resource to the cassava defense community. This genetic material could also be used for the construction of introgression populations, elucidating QTLs with the potential to contribute to the reduction in yield-loss resulting from whitefly and other vector-borne disease states.

## Materials and methods

### Plant growth, insect rearing, and infestation experiments

In vitro-grown cassava (*M. esculenta*) genotypes ECU72 and COL2246 [80, 81] from the CIAT collection were grown for 3 months before use in whitefly-infestation and hormone-treatment experiments as described in Irigoyen et al. [32]. The *Aleurotrachelus socialis* Bondar colony used for cassava infestation experiments were maintained at CIAT as described by Bellotti et al. [8].

*Arabidopsis thaliana* Col-0 seeds (sterilized with chlorine gas and cold-treated for 2 d) were sown on ½ MS 1% sucrose agar plates [82] and kept at room temperature under constant light. One week after plating, seedlings were moved to soil (autoclaved Sunshine Mix (Sun Gro Horticulture, Agawam, MA)) supplemented with 2% Osmocote (w/w) (The Scotts Company, Marysville, OH). Plants were grown in a growth chamber under incandescent and fluorescent lights (180 μE m^−2^ s^−1^) under a short-day light cycle (6-h light/18-h dark) at 24 °C for 27 days, then adjusted to a long-day light cycle (16-h light/8-h dark) for one day before use in hormone-treatment experiments.

All of the plant propagation and experiments performed with plants, hormones and/or insects complied with relevant institutional, national, and international guidelines and legislation.

### Plant hormone treatments

Hormone treatments of ECU72 and COL2246 with SA and MeJA were performed at CIAT as described in Irigoyen et al. [32]. 1-aminocyclopropane-1-carboxylic acid (200 μM ACC, 0.1% EtOH, 0.01% Tween 20) and abscisic acid (200 μM ABA, 0.1% EtOH, 0.01% Tween 20) treatments were performed at CIAT using the same methodology.

SA and JA treatments of 5-week-old Arabidopsis plants were performed in separate rooms at 22–27 °C under incandescent lights (180 μE m^−2^ s^−1^) under a long-day light cycle (16-h light/8-h dark). Rosettes were sprayed until saturation with SA (100 μM SA, 0.1% EtOH, 0.01% Tween 20) or MeJA (100 μM MeJA, 0.1% EtOH, 0.01% Tween 20), with treatments beginning at 6 AM. Leaf tissue was collected at 0, 0.5, 1, 2, 4, 8, 12, and 24 h post treatment and stored at -80 °C until use. All experiments had three biological replicates.

### RNA extraction, cDNA library preparation, sequencing, and data processing

Cassava RNA extraction was performed as described by Behnam et al. [83]. For Arabidopsis leaves, finely-ground frozen tissue was used for RNA extraction, which followed a standard phenol–chloroform phase separation via centrifugation and LiCl precipitation of RNAs [53]. RNA quality assessment, strand-specific cDNA library preparation and RNA-sequencing for cassava and Arabidopsis samples were performed as according to Irigoyen et al. [32]. Libraries were prepared for the three biological replicates of each time point. For libraries from whitefly-infestation experiments, the Illumina NextSeq500 and Illumina HiSeq2500 platforms were used to sequence single-end 75-bp reads (trimmed to 50-bp) and 50-bp reads, respectively. For libraries from Arabidopsis and cassava hormone-treatment experiments, the Illumina NextSeq500 platform was used to sequence single-end 75-bp reads. Twelve to fifteen libraries were multiplexed per lane. An average of ~ 30–50, ~ 13–52 and ~ 20–38 million reads among the three biological replicates were obtained with reproducibility confirmed by Pearson correlation coefficient values of 0.85–1.00, 0.73–0.99 and 0.69–0.98 for Arabidopsis hormone treatments, cassava hormone treatments and cassava whitefly infestations, respectively (Additional file 32: Figures S20-S23). Sequencing and read adaptor removal was carried out at the UCR Institute for Integrative Genome Biology Genomics Core.

Following read adaptor removal, quality filtering was performed. Reads with a Phred quality score below 20 for more than 10 bases were removed. For cassava samples, reads were aligned to the reference *Manihot esculenta* genome version 6.1 with associated annotation information available through Phytozome [84]. For Arabidopsis samples, reads were aligned to the reference *Arabidopsis thaliana* genome version 10 with associated annotation information available through TAIR [85]. Read alignment was performed using HISAT2 [86] and DEG calling using DESeq2 [87]. Read filtering, alignment, and DEG calling were performed using systemPipeR [88]. Detected genes were defined as genes with an average of ≥ 20 reads across a genotype’s treatment time course. Temporal and genotype DEGs (tDEGs and gDEGs, respectively) were identified by comparisons of 0.5–24 hpt to 0 hpt and transcript levels in ECU72 versus COL2246 during treatment, respectively. DEGs were defined as having |log_2_ fold change (FC)|> 1 and false discovery rate (FDR) ≤ 5%. Expression values obtained by RNA-seq were validated by qRT-PCR using seven tDEGs including *PR* and hormone biosynthetic genes (Additional file 33: Figure S24; Additional file 34: Table S47) as in Irigoyen et al. [32].

### Ortholog identification

Hormone- and lignin-pathway genes (Additional files 15 and 30) in *Arabidopsis thaliana* (TAIR ver. 10) [85] were assigned orthologs in the *M. esculenta* genome version 6.1 obtained from Phytozome (JGI) [84] using the online program eggNOG-mapper version 4.5 [89, 90]. Protein sequence queries were matched with the most similar sequence (termed a “seed ortholog”, supported by an e-value ≤ 0.001 and a score ≥ 60) within the eggNOG database. eggNOG orthologous groups (OGs) based on precomputed phylogenies were also assigned. The locations of cassava seed orthologs relative to Arabidopsis genes within an OG phylogenetic tree was used to inform cassava ortholog nomenclature. In the case of Arabidopsis genes equally distant from one or more cassava orthologs, the Arabidopsis gene with the lowest numeral was used for naming (Additional files 15 and 30). Plant-specific OGs (virNOGs) were used in naming cassava orthologs over database-wide OGs (NOGs). All other cassava gene names were obtained from Arabidopsis orthologs annotated for the *M. esculenta* genome (Phytozome ver. 6.1) [84].

### Principal component and correlation analyses

Principal component analyses were performed using the R packages ggplot2 and DESeq2 [87, 91] using count values for all detected genes. Pearson correlation analyses were performed used the R packages ggplot2 (Additional file 32) or corrplot (Additional file 4) [92]. The strength of correlation R-values was defined according to Evans [93] as very weak (|0.00–0.19|), weak (|0.20–0.39|), moderate (|0.40–0.59|), strong (|0.60–0.79|), or very strong (|0.80–1.00|).

### Gene expression clustering and visualization

Ordering of gene expression values by k-means or hierarchical clustering was performed using the base R stats package v3.6.2 and the R package ComplexHeatmap [94], respectively. Heatmaps displaying gene expression values (mean RPKM or log_2_FC) were constructed using the R package ComplexHeatmap. Boxplot or line graphs were constructed using the R package ggplot2. Boxplot whiskers represent values within 1.5 × IQR, and box values represent the first quartile, median, and third quartile values. Arabidopsis ET and ABA microarray data sets (0.5, 1, and 3 hpt) from Goda et al. [34] were compiled for comparison of Arabidopsis and cassava hormone-responsive DEG counts. The R package VennDiagram [95] was used to construct Venn diagrams. Gene expression data is available in Additional files 1, 11 and 17.

### Metabolite quantification and transcript correlation

Relative levels of hormones were obtained from an untargeted metabolomics data set previously prepared, analyzed and published by Perez-Fons et al. [33] and Drapal et al. [96]. The same cassava samples were used in our study. Significant differences (*p* ≤ 0.05) between genotypes ECU72 and COL2246 at each time point for each phytohormone detected were calculated by Student’s T-test (Additional file 22: Table S41).

The methods for detection and characterization of putative glucosinolates were established with a pool containing equal parts of infested ECU72 and COL2246 samples using the collection methods of Perez-Fons et al. [33] and the standard methodologies of Crocoll et al. [97] and Clark [98]. Dried leaf powder (30 mg) was dissolved in 1 mL of 85% methanol and shaken for 4 min at room temperature, centrifuged at 20,000 g for 5 min and the pellet was discarded. An aliquot of 100 μL was used as crude extract and the remaining solution used for in-column sulfatase treatment. Sulfatase (Sigma-Aldrich) solution and DEAE-Sephadex A25 column (Sigma-Aldrich) were prepared as detailed in Crocoll et al. [97]. Briefly, a 200-μL column solution in 20 mM potassium acetate (pH 5) was loaded into 1-mL glass pipette tip with glass wool and remaining crude extract (900 μL) was subsequently loaded onto the column. The column was washed twice with 70% ethanol and water (100 μL each). At this time, 20 μL of the sulfatase solution was added and the reaction incubated overnight at room temperature. Reaction products (desulfonated glucosinolates) were collected by eluting with 200 μL of water and stored at -20° C until LC–MS analysis. The procedure was repeated using a solution of sinigrin and glucotropaeolin (0.5 mg/mL, 200 μL) as references for aliphatic and phenyl derivatives of glucosinolates, respectively, as positive control, and a blank solution (70% ethanol) served as a negative control. In a separate experiment, the samples were prepared by extracting plant material at 100° C for 4 min to inactivate any residual myrosinase activity. Both experiments were compared and no difference in metabolite composition was observed.

For the analysis of the crude extracts and collected fractions, an Agilent’s 1290 UPLC and a 6560 Ion mobility Q-TOF mass spectrometer equipped with an Agilent Jet Stream (AJS) electrospray source was used in negative mode as detailed in Drapal et al. [99]. Compounds were separated in a Zorbax RRHD Eclipse Plus C18 2.1 × 50 mm, 1.8 μm using a two solvents gradient consisting of (A) 2.5% acetonitrile in water and (B) acetonitrile, both solvents containing 0.03% vol. formic acid. Gradient started at 2% B for 1 min, increase to 30% B over 5 min, stay isocratic for 1 min followed by an increase to 90% B in two minutes and stay isocratic for another two minutes. Initial conditions were restored and re-equilibration lasted 3 min. The total runtime per sample was 15 min and flowrate was set at 0.3 mL/min. Nebulizer and sheath gas temperatures were 325° and 275° C, respectively; flowrate of drying and sheath gas (nitrogen) were 5 and 12 L/min respectively and nebulizer pressure 35 psi. Capillary VCap, nozzle and fragmentor voltages were set at 4000, 500 and 400 V respectively. A reference mass solution was continuously infused to ensure mass accuracy calibration at 24–25 K resolution. Injection volume was 1 μL. A mix solution of commercial standards of glucosinolates (sinigrin and glucotropaeolin) were used as reference material and for methodology validation purposes (Additional file 29: Figure S18).

To identify hormone-pathway transcripts with expression levels strongly correlated (R ≥ 0.60) to levels of their corresponding metabolite to confirm metabolite classification, the in-house software Multi-Omics CoAnalysis (MOCA v0.9.9.1, UCR) was used [100–102] (Additional file 23).

### GO term enrichment

The R package ClusterProfiler [103] was used to perform GO-term enrichment analyses of cassava whitefly- and/or hormone-responsive gene sets. Results of enrichment analyses display only those terms within the “biological process (BP)” GO category. Terms were additionally grouped into categories based on shared ancestral GO terms. Significant GO terms are defined as those with adjusted *p*-values ≤ 0.05 and *q*-values ≤ 0.05.

### Supplementary Information

**Additional file 1.** Expression values for hormone-regulated cassava DEGs. **Table S1.** log_2_FC and FDR values of tDEGs in ECU72 after SA treatment. **Table S2.** log_2_FC and FDR values of tDEGs in COL2246 after SA treatment. **Table S3.** log_2_FC and FDR values of tDEGs in ECU72 after JA treatment. **Table S4.** log_2_FC and FDR values of tDEGs in COL2246 after JA treatment. **Table S5.** log_2_FC and FDR values of tDEGs in ECU72 after ET treatment. **Table S6.** log_2_FC and FDR values of tDEGs in COL2246 after ET treatment. **Table S7.** log_2_FC and FDR values of tDEGs in ECU72 after ABA treatment. **Table S8.** log_2_FC and FDR values of gDEGs in ECU72 versus COL2246 after ABA treatment. **Table S9.** log_2_FC and FDR values of gDEGs in ECU72 versus COL2246 after SA treatment. **Table S10.** log_2_FC and FDR values of gDEGs in ECU72 versus COL2246 after JA treatment. **Table S11.** log_2_FC and FDR values of gDEGs in ECU72 versus COL2246 after ET treatment. **Table S12.** log_2_FC and FDR values of gDEGs in ECU72 versus COL2246 after ABA treatment.

**Additional file 2.** Numbers of DEGs identified in cassava after hormone treatments. **Table S13.** Numbers of up- and down-regulated tDEGs in ECU72 and COL2246. **Table S14.** Numbers of up- and down-regulated gDEGs in ECU72 versus COL2246 during hormone treatments.

**Additional file 3: Figure S1.** ET and ABA responses in whitefly-resistant (ECU72) and -susceptible (COL2246) cassava. PCAs of detected gene expression prior to and after ET and ABA treatments (0, 0.5, 1, 2, 4, 8, 12, 24 hpt) in ECU72 and COL2246. Clustering of time points defining early (E) and late (L) response phases in ECU72 versus COL2246 are shown. Detected genes were defined as having an average of 20 RNA-seq reads or more across a hormone-treatment time course. Normalized read count values for three biological replicates per time point are shown. Time points and genotypes are labeled by color and shape, respectively.

**Additional file 4: Fig. S2.** Correlation of SA, JA, ET, and ABA responses in ECU72 and COL2246. (a-b) Correlation matrices of early and late SA, JA, ET, and ABA responses in ECU72 (a) and COL2246 (b). Response phases are defined in Fig. 1c and Additional file 3: Figure S1. Correlation values are based on average log_2_FC values of detected genes in ECU72 and COL2246 and are shaded according to the scale of R-values provided in (b). Non-significant correlation values (*p*>0.05) are not shaded (white). R- and *p*-values are provided in Additional file 5.

**Additional file 5: Table S15.** Pearson correlation R and *p*-values for ECU72 and COL2246 hormone treatments.

**Additional file 6.** Clustering and functional enrichment of gDEGs in ECU72 versus COL2246 during hormone treatment displayed as k-means clusters. **Figure S3.** SA gDEGs. **Figure S4.** JA gDEGs. **Figure S5.** ET gDEGs. **Fig. S6.** ABA gDEGs. SA gDEGs mainly differed due to reciprocal regulation, while reciprocity in JA responses was primarily due to differences in transcript levels at 0 hpt (i.e., Clusters 1, 5 and 6). 71% of gDEGs (4,856 of 6,810 genes) identified in the JA response were gDEGs at 0 h (Additional file 1: Table S10). Differential responses of ECU72 and COL2246 to JA, ET, or ABA was largely attributed to differences in the transcript levels of gDEGs. Categories of significantly enriched (*p* ≤ 0.05) GO terms ranked by adjusted *p*-value are provided for each cluster in Additional file 7. gDEGs were identified by comparisons of transcript levels in ECU72 versus COL2246 during SA treatments and had |log_2_FC| ≥ 1 and FDR ≤ 5%. Boxplot whiskers represent values within 1.5 x IQR, and box values represent the first quartile, median, and third quartile values. Outliers (points beyond whiskers) are not displayed. Lines display average expression values (RPKM) at 0 to 24 hpt.

**Additional file 7.** Enriched GO terms for cassava hormone expression clusters. **Table S16.** Enriched GO terms (biological process) in ECU72 versus COL2246 during SA treatment as displayed in Additional file 6: Figure S3. **Table S17.** Enriched GO terms (biological process) in ECU72 versus COL2246 during JA treatment as displayed in Additional file 6: Figure S4. **Table S18.** Enriched GO terms (biological process) in ECU72 versus COL2246 during ET treatment as displayed in Additional file 6: Figure S5. **Table S19.** Enriched GO terms (biological process) in ECU72 versus COL2246 during ABA treatment as displayed in Additional file 6: Figure S6.

**Additional file 8: Figure S7.** Overlap of Arabidopsis SA or JA tDEGs found in this and external studies. (a-b) Venn diagrams comparing Arabidopsis SA- and JA-responsive tDEGs identified by previous studies that used plants of different ages and different hormone concentrations  [103, 104, 105–110]. Identities of DEGs identified in previous studies are provided in Additional file 9. (c-d) Venn diagrams comparing Arabidopsis SA- and JA-responsive tDEGs identified by our current study and by previous studies. dag = days after germination. DEG identities are provided in Additional files 9 and 11.

**Additional file 9.** Expression values for Arabidopsis SA and JA hormone-treatment tDEGs derived from the literature. **Table S20.** SA tDEGs identified in Arabidopsis from the literature.** Table S21.** JA tDEGs identified in Arabidopsis from the literature.

**Additional file 10: Figure S8.** Temporal responses of Arabidopsis to SA and JA treatments. (a) Arabidopsis tDEG counts during SA and JA treatments (0.5, 1, 2, 4, 8, 12, and 24 hpt). Number of up- and down-regulated genes (red and blue, respectively) are displayed and total number of DEGs for each treatment are provided. Treatment DEGs were identified by comparisons of 0 hpt and 0.5-24 hpt and had |log_2_FC| ≥ 1 and FDR ≤ 5%. DEG expression values are provided in Additional file 11 and DEG counts in Additional file 12. (b) PCAs of detected gene expression prior to and after SA and JA treatments (0, 0.5, 1, 2, 4, 8, 12, 24 hpt) in Arabidopsis. Clustering identified distinct early (E)- and late (L)-response phases with both responses returning to the basal state (0 hpt) by 24 hpt. Detected genes were defined as having an average of 20 reads or more across a hormone-treatment time course. Normalized read count values for three biological replicates are shown per time point. Time points and genotypes are labeled by color and shape, respectively.

**Additional file 11.** Expression values for SA- and JA-regulated Arabidopsis DEGs. **Table S22.** log_2_FC and FDR values of tDEGs in Arabidopsis after SA treatment. **Table S23.** log_2_FC and FDR values of tDEGs in Arabidopsis after JA treatment.

**Additional file 12: Table S24.** Numbers of up- and down-regulated tDEGs in Arabidopsis during hormone treatments

**Additional file 13.** Expression values of ET- and ABA-regulated DEGs in Arabidopsis after ET or ABA treatment from external datasets. **Table S25.** log_2_FC and *p*-values or signal ratio values of tDEGs in Arabidopsis after ET treatment from the Goda et al. [34] microarray data sets. **Table S26.** log_2_FC and *p*-values or signal ratio values of tDEGs in Arabidopsis after ABA treatment from the Goda et al. [34] microarray data sets.

**Additional file 14.** Hormone-pathway gene expression in Arabidopsis and two cassava genotypes. **Figure S9.** SA-pathway genes. Many species differences were observed, including weaker induction of *MeICS1* than *AtICS1* and reciprocal regulation of genes between Arabidopsis and one of the two genotypes (biosynthesis gene *MePAL1a*-*c *and signaling genes* MeCBP60a1*, *MeCBP60b1-2*, *MeSMTb*-c, *MeGRX480a *and *c*, *MeNPR1*, *MeSARD1a*, *MeWRKY70a-b, and MeEDS5a-c*). **Figure S10.** ET-pathway genes. Many species differences were observed, including differing regulation of the *ACS* family biosynthesis genes and key JA/ET pathway signaling gene *AtERF1*. **Figure S11.** ABA-pathway genes. Many species differences were observed, including reciprocal regulation of genes between Arabidopsis and one of the two genotypes (biosynthesis genes: *MePDS*, *MeABA4*, *MeNCED2a*, *MeNCED3a* and *b*; modification genes: *CYP707A1a *and *b*; and signaling genes: *MeAGH3a, MeATHB7a *and* b, MeHAI1a *and* b*, and MeNAC019c). **Figure S12.** JA-pathway genes. Species generally responded similarly. Genes involved in biosynthesis, modification, transport and in transducing or responding to the hormone (signaling) were identified from the literature and cassava orthologs were identified (Additional file 15). Expression of hormone-pathway genes detected during hormone treatment in Arabidopsis, ECU72 and COL2246 are presented as log_2_FC values. Biosynthetic genes are ordered by their approximate step in the pathway, while other genes are ordered alphabetically. To enable comparison, orthologous genes in Arabidopsis and cassava are denoted by box color. Undetected genes are shown in grey.

**Additional file 15.** Hormone-pathway gene nomenclature for cassava. **Table S27.** Cassava’s SA-pathway gene nomenclature. **Table S28.** Cassava’s JA-pathway gene nomenclature. **Table S29.** Cassava’s ET-pathway gene nomenclature. **Table S30.** Cassava’s ABA-pathway gene nomenclature.

**Additional file 16: Table S31.** SA-, JA-, ET- and ABA-pathway gene numbers in Arabidopsis and cassava.

**Additional file 17.** Expression values for whitefly-regulated cassava DEGs. **Table S32.** log_2_FC and FDR values of tDEGs in ECU72 after whitefly infestation. **Table S33.** log_2_FC and FDR values of tDEGs in COL2246 after whitefly infestation. **Table S34.** log_2_FC and FDR values of gDEGs in ECU72 versus COL2246 after whitefly infestation.

**Additional file 18.** Numbers of DEGs identified in cassava after whitefly treatment. **Table S35.** Numbers of up- and down-regulated tDEGs in ECU72 and COL2246 during whitefly infestation. **Table S36.** Numbers of up- and down-regulated gDEGs in ECU72 versus COL2246 during whitefly infestation.

**Additional file 19: Figure S13.** Clustering and functional enrichment of genes differentially expressed in ECU72 versus COL2246 during whitefly infestation. Whitefly-responsive gDEGs were grouped into six k-means clusters. For each cluster, enriched GO term categories (*p* ≤ 0.05) were ranked by adjusted p-value (Additional file 20). Cluster 1 and 2 had similar temporal regulatory programs, while Clusters 3-6 were distinct. Cluster 2 genes were enriched for cell-wall-related processes while all other cluster were enriched for processes related to defense such as response to biotic stimulus, response to stimulus, or immune system process. gDEGs were identified by comparisons of transcript levels in ECU72 versus COL2246 during whitefly infestation and had |log_2_FC| ≥ 1 and FDR ≤ 5%. Boxplot whiskers represent values within 1.5 x IQR, and box values represent the first quartile, median, and third quartile values. Outliers (points beyond whiskers) are not displayed. Lines display average expression values (RPKM) at 0 to 22 dpi.

**Additional file 20: Table S37.** Enriched GO terms (biological process) in ECU72 versus COL2246 during whitefly infestation displayed in Additional file 19: Figure S13.

**Additional file 21.** Numbers and percentages of hormone-regulated whitefly-responsive cassava DEGs. **Table S38.** Numbers and percents of hormone-regulated whitefly-responsive tDEGs in ECU72 and COL2246.** Table S39.** Numbers and percents of hormone-regulated WF-responsive gDEGs in ECU72 vs COL2246.

**Additional file 22.** Metabolite measurements in whitefly-infested cassava leaves. **Table S40.** Identification of defense hormones and their derivatives by untargeted metabolomics in whitefly-infested cassava.** Table S41.** Student’s T-test *p*-values comparing identified hormone levels in ECU72 versus COL2246 during whitefly infestation.

**Additional file 23: Figure S14.** Metabolite-transcript correlations in the SA, JA and ABA pathways. Correlations of SA, SAG, MeJA, 12-OPDA, ABA, or PA levels with cassava hormone pathway transcripts expressed during whitefly infestation were identified using the in-house software Multi-Omics CoAnalysis (MOCA). All panels display relative abundance (measured as EIC area) of a hormone during whitefly infestation and expression levels (RPKM) of genes correlated to that hormone belonging to the associated hormone pathway. (a) SA and correlated SA-pathway genes. (b) SAG and correlated SA-pathway genes. (c) MeJA and correlated JA-pathway genes. (d) 12-OPDA and correlated JA-pathway genes. (e) ABA and correlated ABA-pathway genes. (f) PA and correlated ABA-pathway genes. SA, SAG, MeJA, 12-OPDA, ABA, and PA were moderately (0.40 ≤ R ≤ 0.59) to very strongly correlated (0.80 ≤ R ≤ 1.00) with 994, 1,138, 1,146, 1,401, 1,211, and 3,978 transcripts that were responsive to *A. socialis *infestation. In particular, transcript levels of several genes belonging to the SA, JA and ABA pathways were correlated with changes in the levels of SA/SAG, MeJA/OPDA, and ABA/PA, respectively, during infestation. ABA levels in ECU72 and COL2246 were moderately correlated (0.40 ≤ R ≤ 0.59) with transcript levels of the ABA biosynthetic gene *MeAAO1d* during infestation. Similarly, strong (0.60 ≤ R ≤ 0.79) to very strong correlations (0.80 ≤ R ≤ 1.00) were detected between jasmonates and their biosynthetic gene *MeLOX2a*. Only significant moderate to strong correlations with R ≥ 0.40 and *p*-value ≤ 0.05 are displayed. The mean and individual biological replicate values are displayed with error bars representing Standard error of the mean (S.E.M.). Asterisks (*) indicate significant difference in hormone level between genotypes as identified by Student’s t-test (*p* ≤ 0.05) (Additional file 22: Table S41).

**Additional file 24: Figure S15.** Cassava hormone-pathway gene expression during whitefly and hormone treatments. Hormone-pathway genes that were also gDEGs during whitefly infestation are displayed as log_2_FC values during whitefly infestation (1, 7, 14, and 22 dpi) and SA, JA, ET, and ABA treatments (0.5, 1, 2, 4, 8, 12, 24 hpt). Genes are clustered by whitefly expression then hierarchically. The number of gDEGs associated with biosynthesis (B), modification (M), signaling (S), and transport (T) for each hormone is provided on the right of each cluster. Genes that were induced in ECU72 but repressed in COL2246 during infestation (Cluster 3) included five ABA signaling genes (*MeAHG1*, *MeAHG3b,* *MeABI1a *and *MeHAI1a and b*), two ABA-response genes (*MeABF2a* and *MeABF3*), five ET-responsive *PR3 *chitinase genes, and two SA-modification genes, which convert SA to an inactive form (*UGT74F1b*-*c*).

**Additional file 25.** Enriched GO terms for whitefly and hormone gDEGs. **Table S42.** Enriched GO terms (biological process) in ECU72 versus COL2246 and in COL2246 versus ECU72 during whitefly infestation and hormone treatment displayed in Fig. 5. **Table S43.** Enriched GO terms (biological process) in ECU72 versus COL2246 and in COL2246 vs ECU72 during whitefly infestation and hormone treatment displayed in Additional file 27: Figure S16.

**Additional file 26.** Counts of enriched GO term IDs for whitefly and hormone gDEGs. **Table S44.** Numbers of genes associated with biological GO term categories associated with defense displayed in Fig. 5. **Table S45.** Numbers of genes associated with GO-term categories (not defense-related) displayed in Additional file 27: Figure S16.

**Additional file 27: Figure S16.** Functional enrichment of cassava gDEGs in response to whitefly and hormone treatments, non-defense categories. GO-term enrichment was performed on whitefly gDEGs. Numbers of genes enriched for terms not linked to defense are shown. gDEGs responsive to one-three hormones, all hormones, or that are hormone-nonresponsive (WF) are shown. Genes upregulated in ECU72 or COL2246 are displayed on the right and left sides of the x-axis, respectively. Counts and identities of genes within each GO term category are provided in Additional file 25: Table S43 and Additional file 26: Table S45.

**Additional file 28: Figure S17.** Expression of whitefly- and hormone-regulated gDEGs enriched in ECU72 versus COL2246 during infestation. The expression of a selection of gDEGs from five enriched GO-term categories (cell wall organization or biogenesis, glucosinolate metabolism, lipid metabolism, small molecule metabolism, and immune system process) from Fig. 5 are shown. Individual values (circles) and average RPKM values (lines) are shown. Among cell-wall-related genes, several lignin biosynthetic genes (*MeCOMTf*, *MeCCOAMTa*, *MeMYB63*, *MeLAC4*, and *MeLAC5*) were identified. Genes are grouped based on the magnitude of their response (RPKM values) and denoted in different colors. Gene loci are listed in Additional file 15 or as follows: *MeGUX1* (Manes.12G153100), *MeCESA4* (Manes.13G038000), *MeLAC4* (Manes.08G089800), *MeLAC5* (Manes.07G135400), *MeXTH23* (Manes.05G108100), *MeMYB63* (Manes.06G175200), *MePME31* (Manes.02G048600), *MeSOT17* (Manes.10G085400), *MeCYP83B1a* (Manes.05G150100), *MeCYP83B1b* (Manes.05G139500), *MeCYP83B1c* (Manes.S058500), *MePEPCK* (Manes.18G054700), *MeOSM34a* (Manes.01G064200), *MeOSM34b* (Manes.01G064300), *MeRPM1* (Manes.18G117800), *MePIP5K1* (Manes.S096300), *MeGULLO3* (Manes.03G061900).

**Additional file 29: Figure S18.** Analysis of glucosinolates in cassava leaves. (a) Scheme of sulfatase reaction and its effect on glucosinolate structure. (b) LC-MS measurements of glucosinolates standard solutions treated with sulfatase. (c) Total ion chromatograms (TIC) of leaf extracts before (crude) and after (desulfonated) the sulfatase reaction and extracted ion chromatogram (EIC) of thioglucose fragment, as indicator of presence of glucosinolated structures.

**Additional file 30: Table S46. **Cassava’s lignin biosynthesis pathway gene nomenclature.

**Additional file 31: Figure S19.** Expression of whitefly- and hormone-regulated gDEGs enriched in COL2246 versus ECU72 during infestation. The expression of a selection of gDEGs from four enriched GO-term categories (immune system process, oxidative stress, starch metabolism, and terpenoid metabolism) from Fig. 5 are shown. Individual values (circles) and average RPKM values (lines) are shown. Genes are grouped based on the magnitude of their response (RPKM values) and denoted in different colors. Gene loci are listed in Additional file 15 or as follows: *MeBIR1a* (Manes.01G019000), *MeBIR1e* (Manes.13G056500), *MeFMO1* (Manes.16G091800), *MePEPR1* (Manes.16G045200), *MeRPP8* (Manes.10G023300), *MeCNL* (Manes.11G053000), *MePERK1a* (Manes.11G039800), *MePERK1b* (Manes.11G041400), *MePERK1c* (Manes.11G042500), *MeNDR1* (Manes.03G123200), *MeWRKY41* (Manes.02G011500), *MeWRKY53* (Manes.01G047200), *MePA2* (Manes.15G104300), *MeSBE2*.2 (Manes.09G059400), *MeISA3* (Manes.18G063500), *MeSEX1* (Manes.13G026800), *MeSEX4* (Manes.10G053500), *MeTPS21a* (Manes.02G086100), *MeTPS21b* (Manes.02G086300), *MeTPS21c* (Manes.18G101900).

**Additional file 32.** Replicate correlations for cassava and Arabidopsis treatment samples. **Figure S20.** Arabidopsis (a) SA and (b) JA treatments. **Figure S21.** (a) ECU72 and (b) COL2246 whitefly treatments. **Figure S22.** ECU72 and COL2246 (a,b) SA and (c,d) JA treatments. **Figure S23.** ECU72 and COL2246 (a,b) ET and (c,d) ABA treatments. Pearson correlation coefficient (R) and *p*-values between biological replicates (R1-R3) were calculated for genes detected during treatments. Detected genes had an average of 20 reads or more across a hormone-treatment or whitefly infestation time course. Normalized read count values for three biological replicates are shown per time point. RNA-seq read count values for each time point are labeled by color.

**Additional file 33: Figure S24.** qRT-PCR validation of RNA-seq expression values. (a) The *in silico* and qRT-PCR relative expression values of hormone-biosynthetic genes *MePAL1c*, *MeLOX3a*, *MeACS6b*, and *MeAAO1c* following SA, JA, ET, and ABA treatments of ECU72 and COL2246. qRT-PCR of 0.5, 1 and 2 hpt samples confirmed the RNA-seq data. (b) The *in* *silico* and qRT-PCR relative expression values of sentinel* PR *gene *MePR-9e* after whitefly infestation in ECU72 at 14 and 22 dpi. Expression of *MePR-9e* in COL2246 was previously determined [32]. (c) The *in silico* and qRT-PCR relative expression values of *AtPAL1* and *AtLOX3 *following SA and JA treatments, respectively, were confirmed in *Arabidopsis thaliana* at 0.5, 1 and 2 hpt *in vivo*. Bargraphs display qRT-PCR sample values (overlayed points) as well as the average and standard error (SE) of three biological replicates (error-bar graph). For *MeAAO1c* in COL2246 at 1 hpt (a), only two biological replicates are provided. Primers are provided in Additional file 34. (d) A scatter plot with Pearson correlations demonstrated the relative expression determined by qRT-PCR and RNA-seq showed a strong and significant positive correlation for all biological replicates displayed in panels a-c. All expression values were normalized to *MeUBQ* in cassava and *AtACT7* in Arabidopsis and are relative to the treatment’s 0-h time point.

**Additional file 34: Table S47.** qRT-PCR primers.

## Data Availability

RNA-seq reads are deposited at the NCBI Short Read Archive (SRA) (https://www.ncbi.nlm.nih.gov/sra) under the accession number: PRJNA814249. Metabolomics data sets of raw (areas) and processed data (normalized) are accessible in Mendeley Data and Digital Commons Data (https://data.mendeley.com/datasets/9y774gbp8v/1) [104] and are also made available by Drapal et al. [96]. All data analyzed during this study are included in this published article (and its supplementary information files).

## Abbreviations

ABA: Abscisic acid
BP: Biological process
DAMP: Damage-associated molecular pattern
DEG: Differentially expressed gene
dpi: Days post infestation
EIC: Extracted ion chromatogram
ET: Ethylene
FDR: False discovery rate
FC: Fold change
gDEG: Genotype DEG
HAMP: Herbivore-associated molecular pattern
hpt: Hours post treatment
JA: Jasmonic acid
MeJA: Methyl jasmonate
MeSA: Methyl salicylate
12-OPDA: 12-Oxo-phytodienoic acid
PAMP: Pathogen-associated molecular pattern
PA: Phaseic acid
PCA: Principal component analysis
PR: Pathogenesis-related
ROS: Reactive oxygen species
SA: Salicylic acid
SAG: SA glucoside
SGE: SA glucose ester
S.E.M: Standard error of the mean
tDEG: Temporal DEG
TIC: Total ion chromatogram
