# Supplementary figures and images for: Integrative transcriptomics reveals association of abscisic acid and lignin pathways with cassava whitefly resistance
Source: BMC Plant Biol. 2023 Dec 20;23:657. doi: 10.1186/s12870-023-04607-y (PMC10731783; doi:10.1186/s12870-023-04607-y)

**Figure S1**

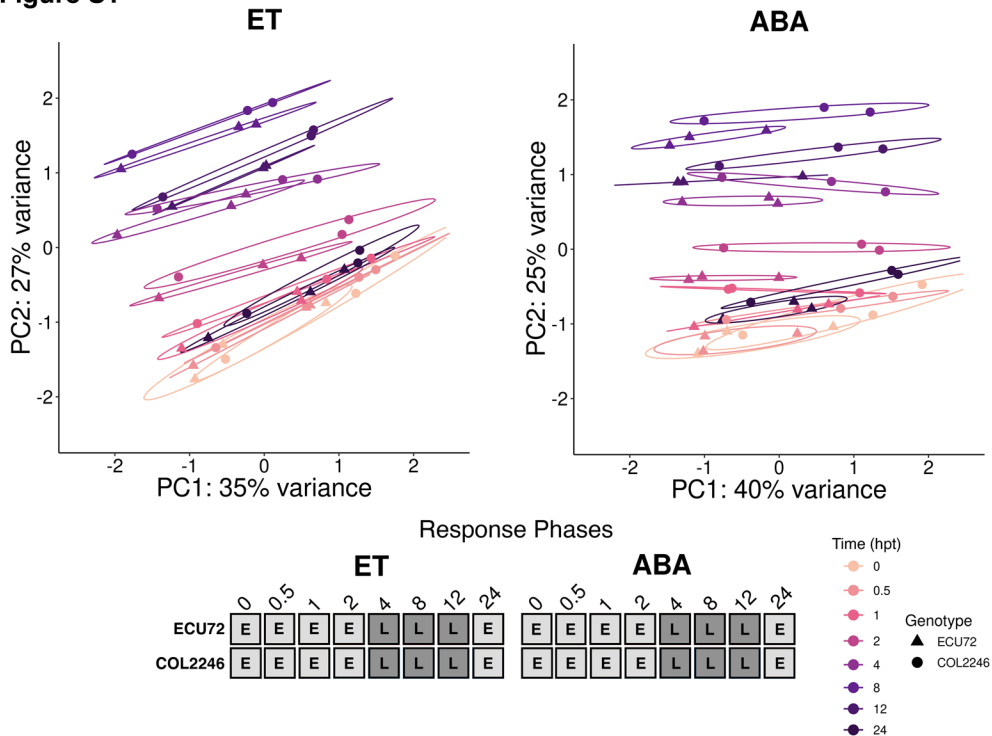

Supplement: Supplementary file 3 — Additional file 3: Figure S1. ET and ABA responses in whitefly-resistant (ECU72) and -susceptible (COL2246) cassava. PCAs of detected gene expression prior to and after ET and ABA treatments (0, 0.5, 1, 2, 4, 8, 12, 24 hpt) in ECU72 and COL2246. Clustering of time points defining early (E) and late (L) response phases in ECU72 versus COL2246 are shown. Detected genes were defined as having an average of 20 RNA-seq reads or more across a hormone-treatment time course. Normalized read count values for three biological replicates per time point are shown. Time points and genotypes are labeled by color and shape, respectively. [file 12870_2023_4607_MOESM3_ESM.pdf]

**Figure S2**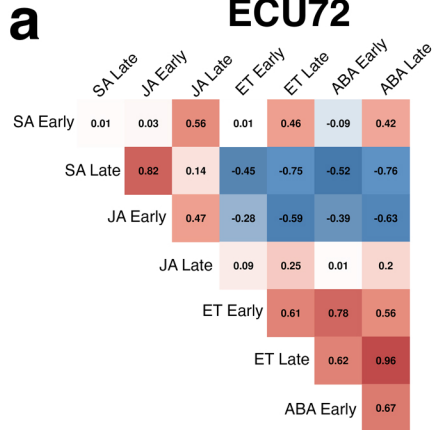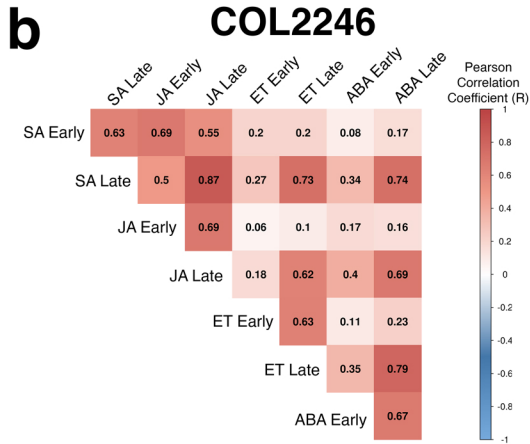

Supplement: Supplementary file 4 — Additional file 4: Fig. S2. Correlation of SA, JA, ET, and ABA responses in ECU72 and COL2246. (a-b) Correlation matrices of early and late SA, JA, ET, and ABA responses in ECU72 (a) and COL2246 (b). Response phases are defined in Fig. 1c and Additional file 3: Figure S1. Correlation values are based on average log2FC values of detected genes in ECU72 and COL2246 and are shaded according to the scale of R-values provided in (b). Non-significant correlation values (p>0.05) are not shaded (white). R- and p-values are provided in Additional file 5. [file 12870_2023_4607_MOESM4_ESM.pdf]

Figure S7

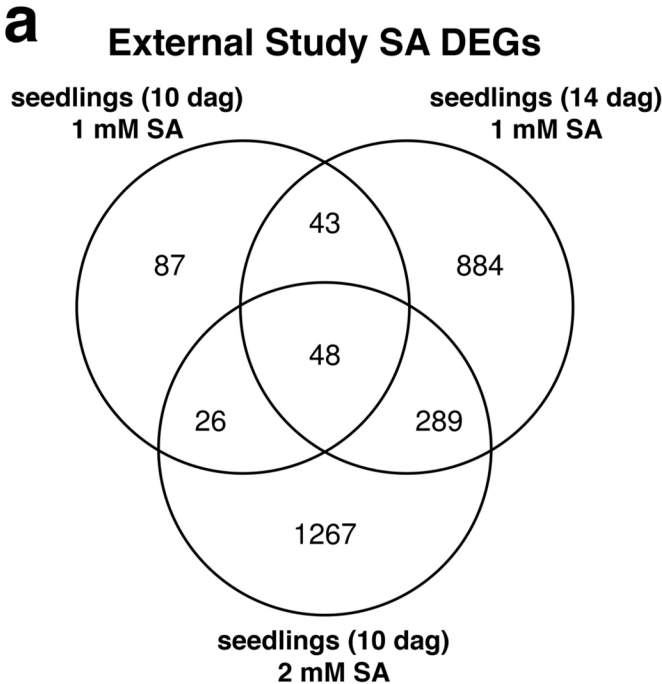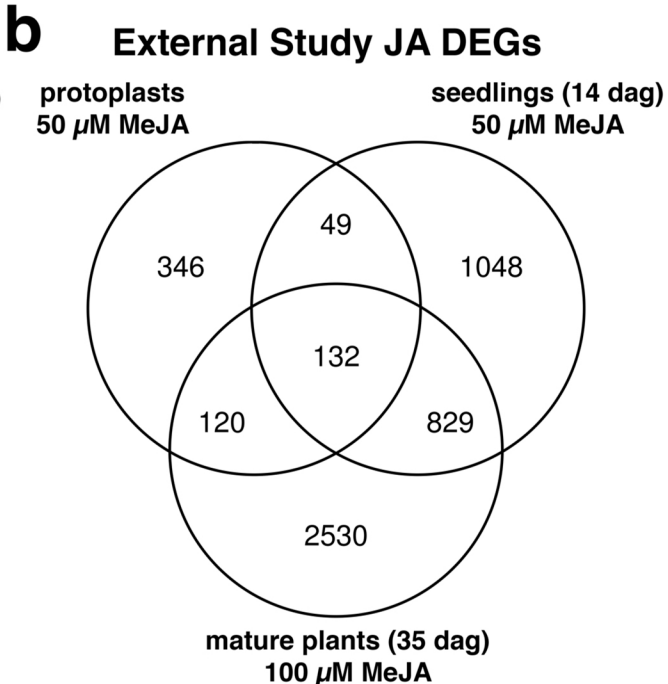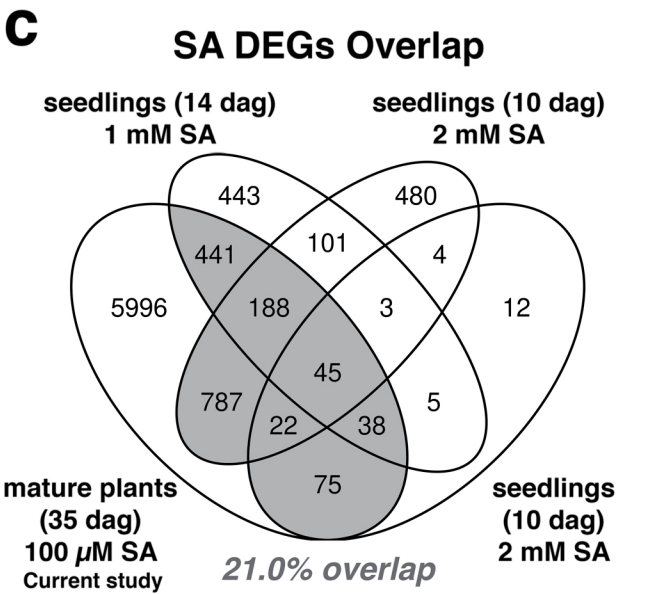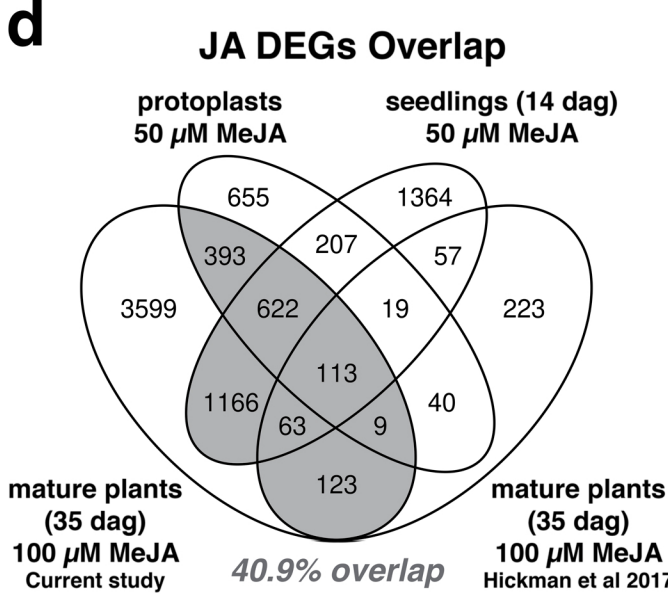

Supplement: Supplementary file 8 — Additional file 8: Figure S7. Overlap of Arabidopsis SA or JA tDEGs found in this and external studies. (a-b) Venn diagrams comparing Arabidopsis SA- and JA-responsive tDEGs identified by previous studies that used plants of different ages and different hormone concentrations [103, 104, 105–110]. Identities of DEGs identified in previous studies are provided in Additional file 9. (c-d) Venn diagrams comparing Arabidopsis SA- and JA-responsive tDEGs identified by our current study and by previous studies. dag = days after germination. DEG identities are provided in Additional files 9 and 11. [file 12870_2023_4607_MOESM8_ESM.pdf]

Figure S8

# a

## Arabidopsis

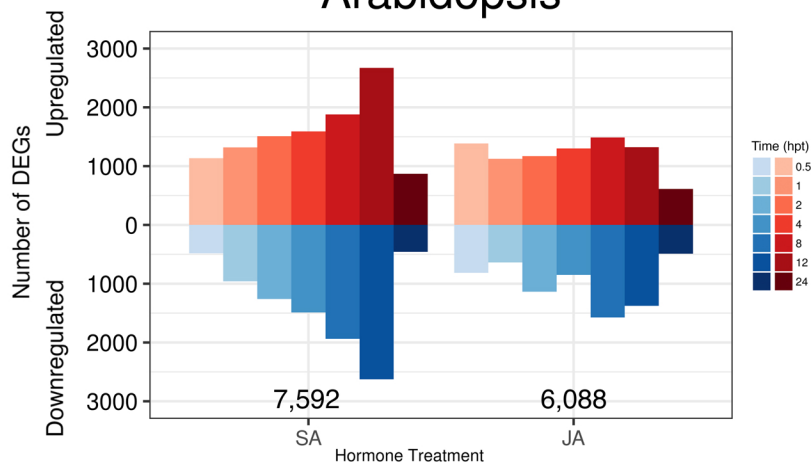

# b

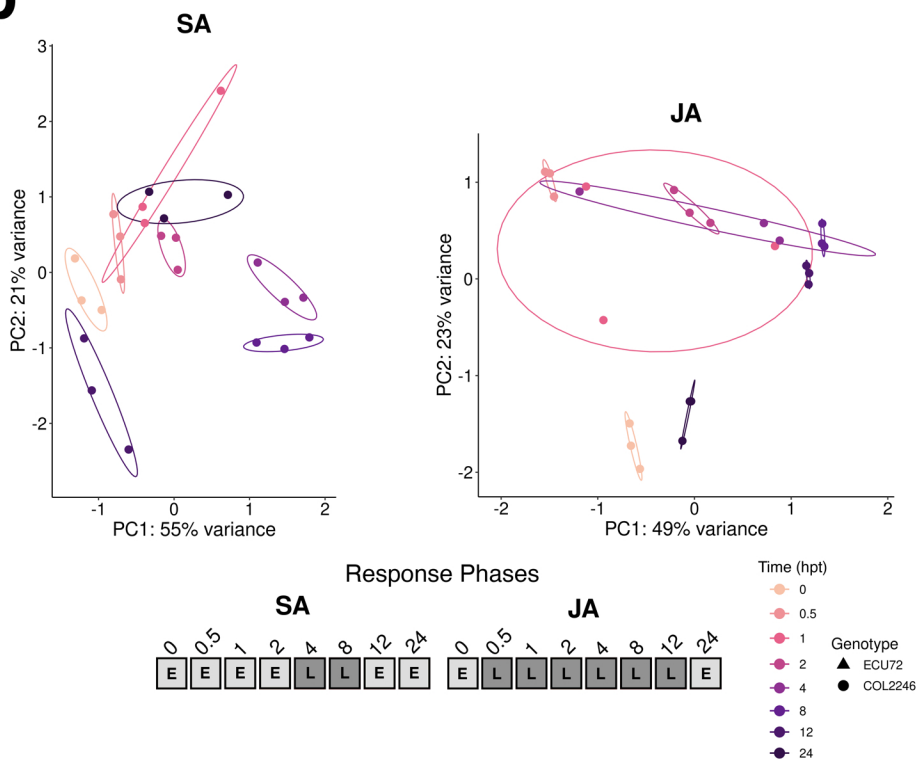

Supplement: Supplementary file 10 — Additional file 10: Figure S8. Temporal responses of Arabidopsis to SA and JA treatments. (a) Arabidopsis tDEG counts during SA and JA treatments (0.5, 1, 2, 4, 8, 12, and 24 hpt). Number of up- and down-regulated genes (red and blue, respectively) are displayed and total number of DEGs for each treatment are provided. Treatment DEGs were identified by comparisons of 0 hpt and 0.5-24 hpt and had |log2FC| ≥ 1 and FDR ≤ 5%. DEG expression values are provided in Additional file 11 and DEG counts in Additional file 12. (b) PCAs of detected gene expression prior to and after SA and JA treatments (0, 0.5, 1, 2, 4, 8, 12, 24 hpt) in Arabidopsis. Clustering identified distinct early (E)- and late (L)-response phases with both responses returning to the basal state (0 hpt) by 24 hpt. Detected genes were defined as having an average of 20 reads or more across a hormone-treatment time course. Normalized read count values for three biological replicates are shown per time point. Time points and genotypes are labeled by color and shape, respectively. [file 12870_2023_4607_MOESM10_ESM.pdf]

Figure S9

SA

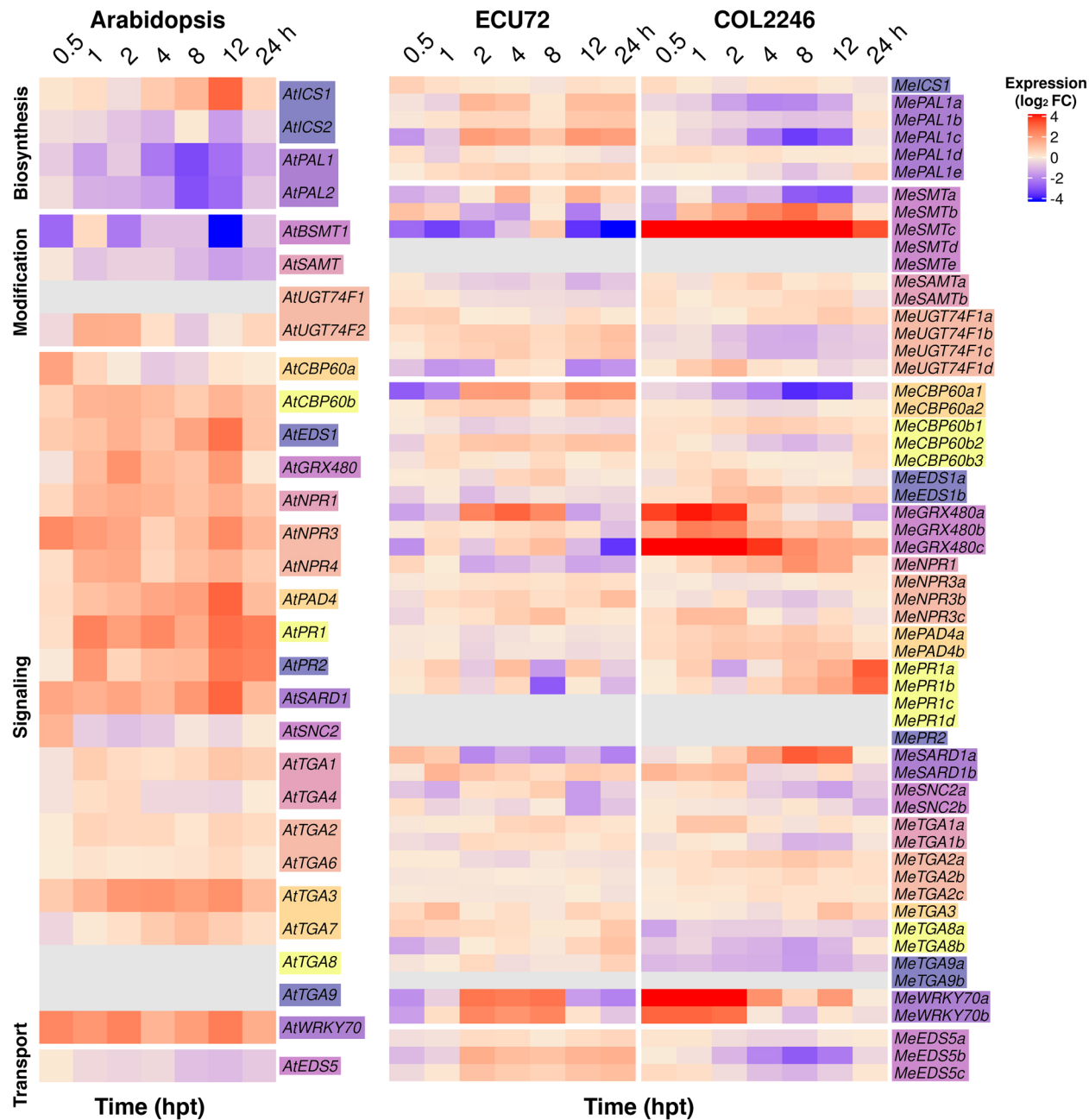

Figure S10

ET

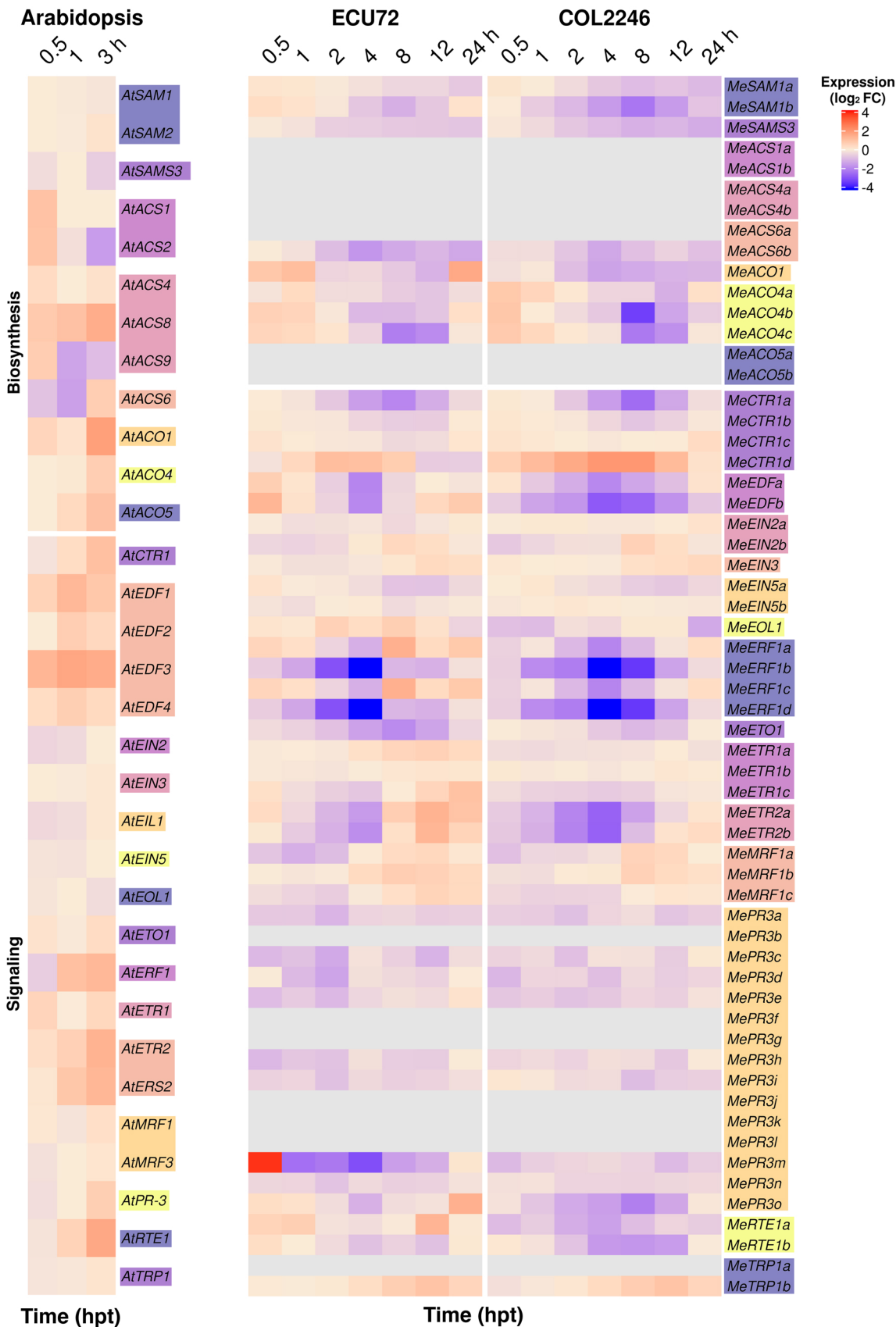

Figure S11

ABA

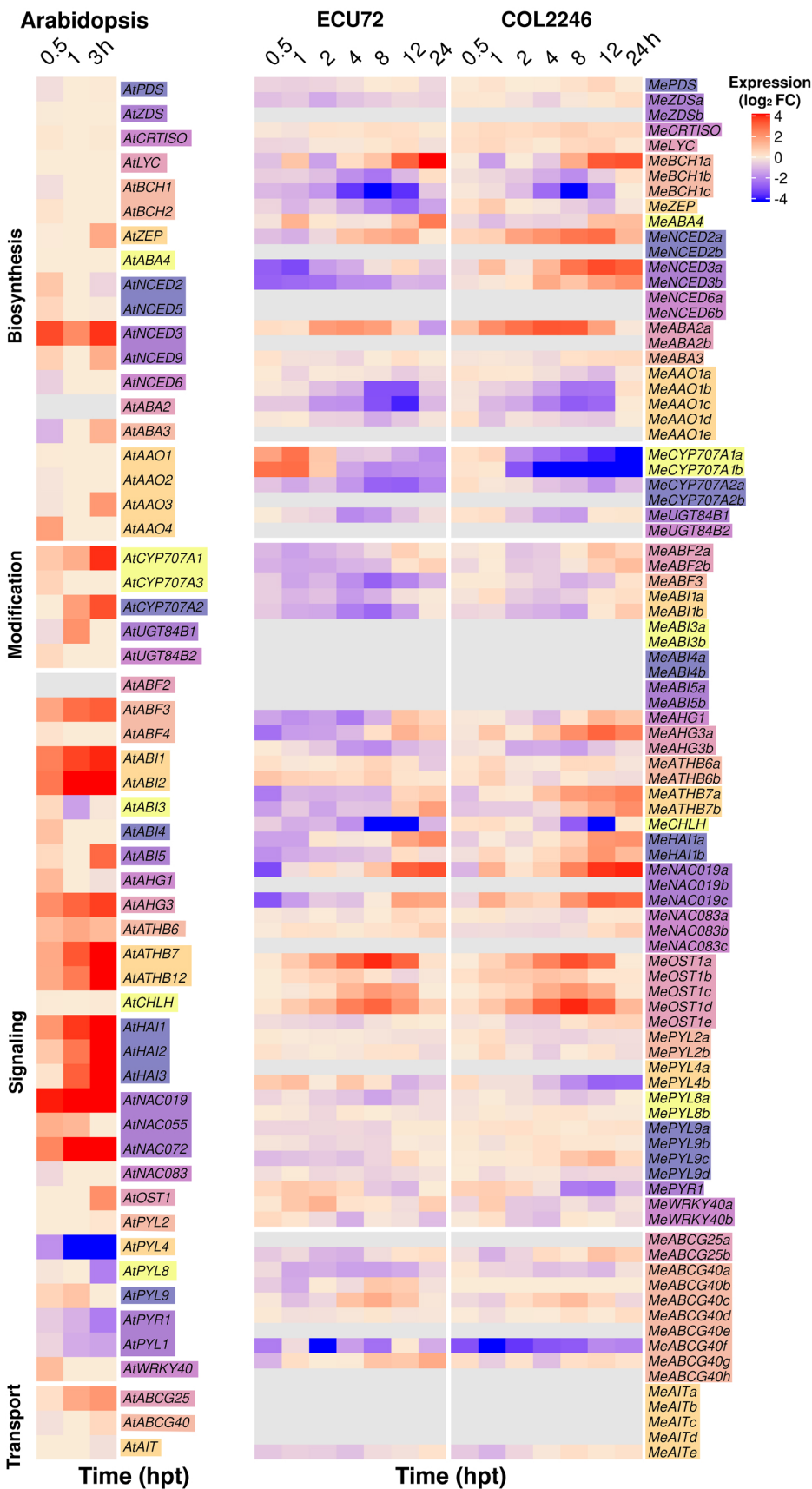

Figure S12

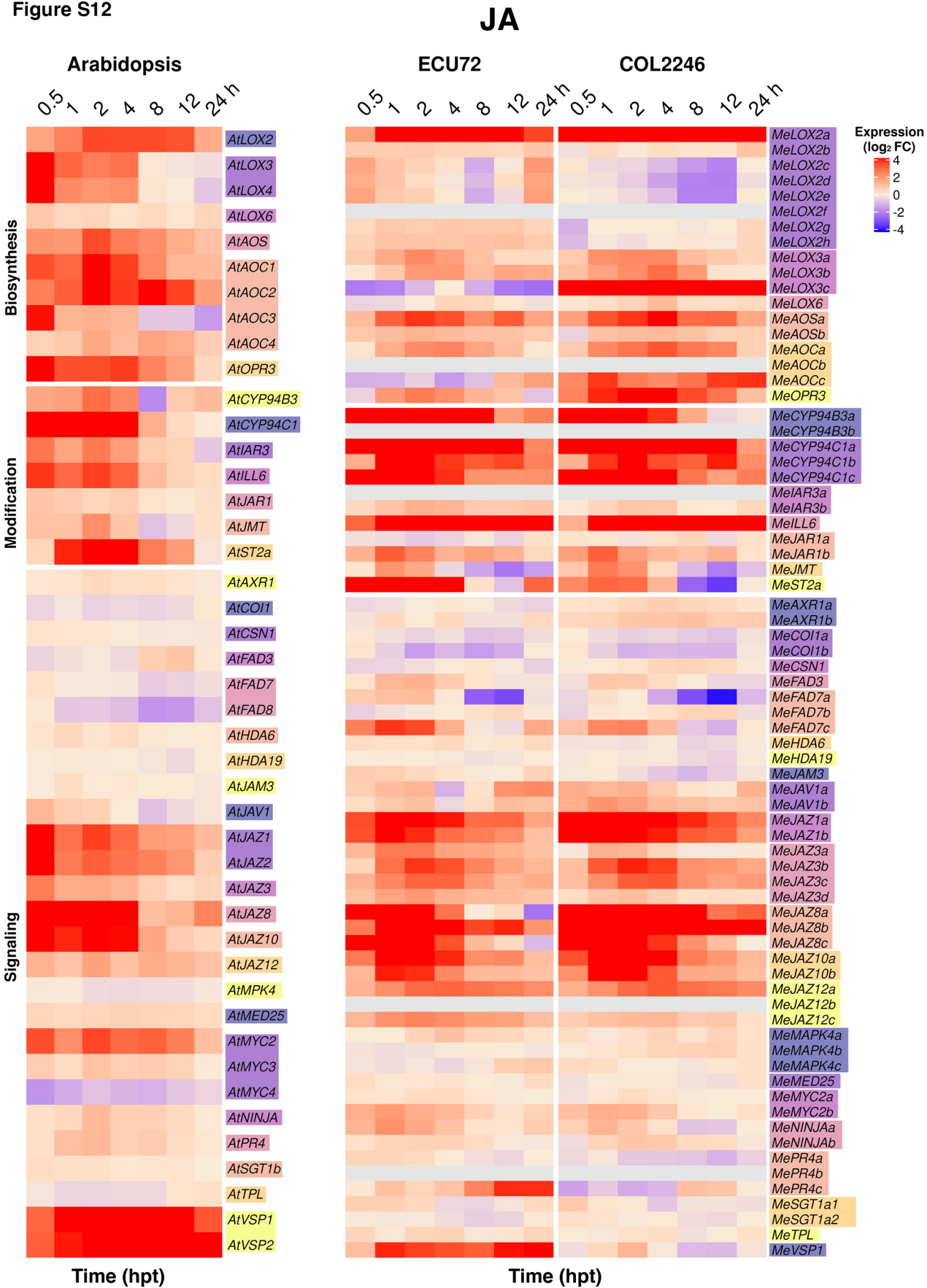

Supplement: Supplementary file 14 — Additional file 14. Hormone-pathway gene expression in Arabidopsis and two cassava genotypes. Figure S9. SA-pathway genes. Many species differences were observed, including weaker induction of MeICS1 than AtICS1 and reciprocal regulation of genes between Arabidopsis and one of the two genotypes (biosynthesis gene MePAL1a-c and signaling genes MeCBP60a1, MeCBP60b1-2, MeSMTb-c, MeGRX480a and c, MeNPR1, MeSARD1a, MeWRKY70a-b, and MeEDS5a-c). Figure S10. ET-pathway genes. Many species differences were observed, including differing regulation of the ACS family biosynthesis genes and key JA/ET pathway signaling gene AtERF1. Figure S11. ABA-pathway genes. Many species differences were observed, including reciprocal regulation of genes between Arabidopsis and one of the two genotypes (biosynthesis genes: MePDS, MeABA4, MeNCED2a, MeNCED3a and b; modification genes: CYP707A1a and b; and signaling genes: MeAGH3a, MeATHB7a and b, MeHAI1a and b, and MeNAC019c). Figure S12. JA-pathway genes. Species generally responded similarly. Genes involved in biosynthesis, modification, transport and in transducing or responding to the hormone (signaling) were identified from the literature and cassava orthologs were identified (Additional file 15). Expression of hormone-pathway genes detected during hormone treatment in Arabidopsis, ECU72 and COL2246 are presented as log2FC values. Biosynthetic genes are ordered by their approximate step in the pathway, while other genes are ordered alphabetically. To enable comparison, orthologous genes in Arabidopsis and cassava are denoted by box color. Undetected genes are shown in grey. [file 12870_2023_4607_MOESM14_ESM.pdf]

**Figure S14**

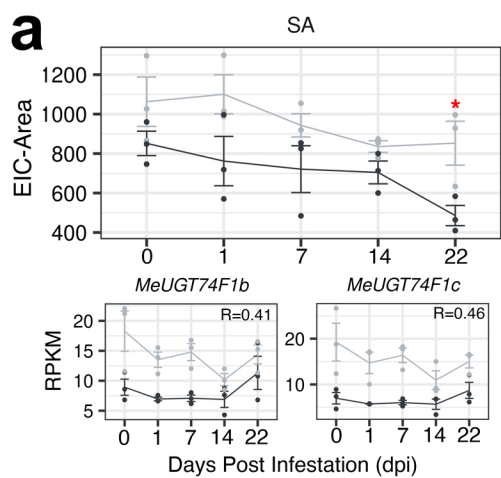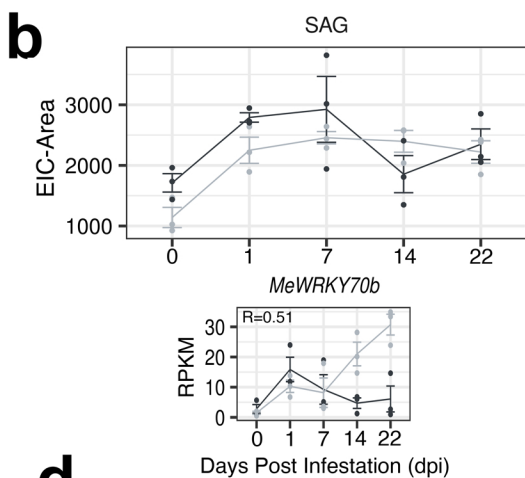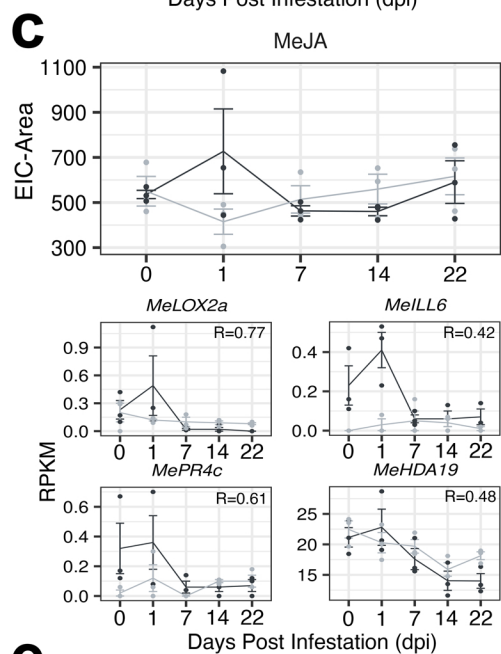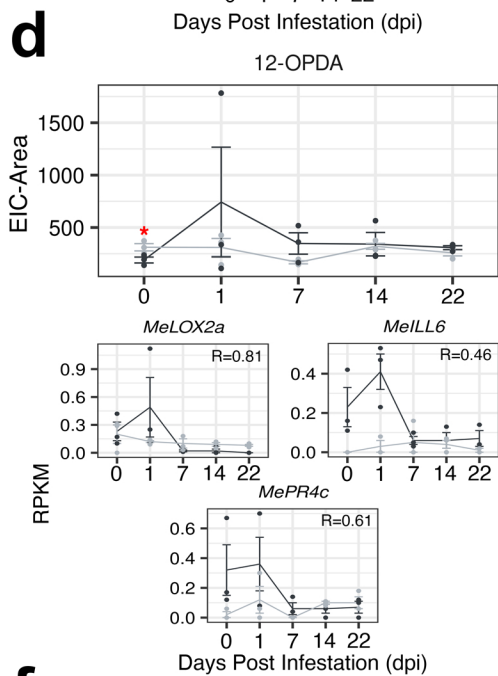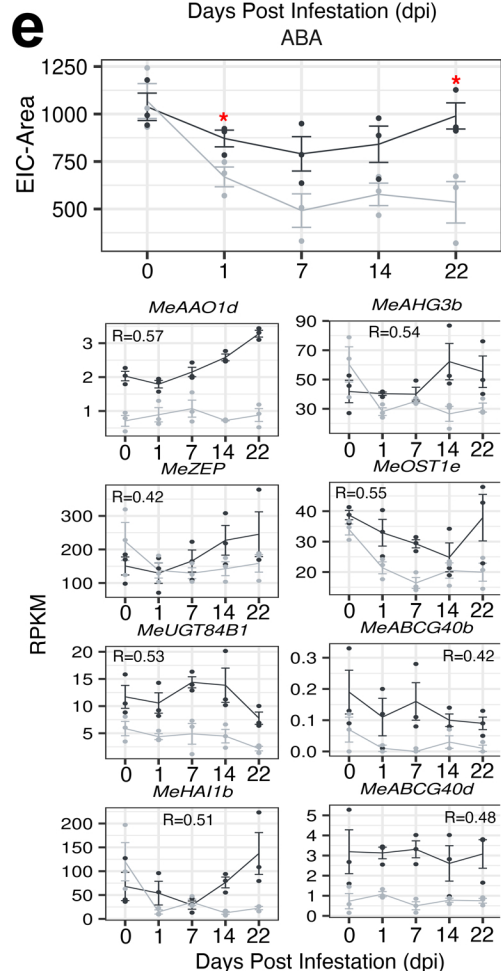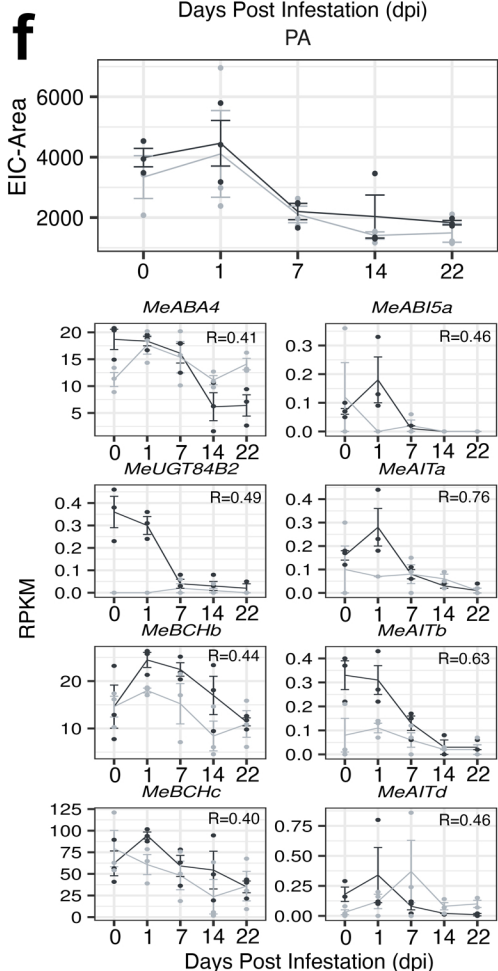

Genotype — ECU72 — COL2246

Supplement: Supplementary file 23 — Additional file 23: Figure S14. Metabolite-transcript correlations in the SA, JA and ABA pathways. Correlations of SA, SAG, MeJA, 12-OPDA, ABA, or PA levels with cassava hormone pathway transcripts expressed during whitefly infestation were identified using the in-house software Multi-Omics CoAnalysis (MOCA). All panels display relative abundance (measured as EIC area) of a hormone during whitefly infestation and expression levels (RPKM) of genes correlated to that hormone belonging to the associated hormone pathway. (a) SA and correlated SA-pathway genes. (b) SAG and correlated SA-pathway genes. (c) MeJA and correlated JA-pathway genes. (d) 12-OPDA and correlated JA-pathway genes. (e) ABA and correlated ABA-pathway genes. (f) PA and correlated ABA-pathway genes. SA, SAG, MeJA, 12-OPDA, ABA, and PA were moderately (0.40 ≤ R ≤ 0.59) to very strongly correlated (0.80 ≤ R ≤ 1.00) with 994, 1,138, 1,146, 1,401, 1,211, and 3,978 transcripts that were responsive to A. socialis infestation. In particular, transcript levels of several genes belonging to the SA, JA and ABA pathways were correlated with changes in the levels of SA/SAG, MeJA/OPDA, and ABA/PA, respectively, during infestation. ABA levels in ECU72 and COL2246 were moderately correlated (0.40 ≤ R ≤ 0.59) with transcript levels of the ABA biosynthetic gene MeAAO1d during infestation. Similarly, strong (0.60 ≤ R ≤ 0.79) to very strong correlations (0.80 ≤ R ≤ 1.00) were detected between jasmonates and their biosynthetic gene MeLOX2a. Only significant moderate to strong correlations with R ≥ 0.40 and p-value ≤ 0.05 are displayed. The mean and individual biological replicate values are displayed with error bars representing Standard error of the mean (S.E.M.). Asterisks (*) indicate significant difference in hormone level between genotypes as identified by Student’s t-test (p ≤ 0.05) (Additional file 22: Table S41). [file 12870_2023_4607_MOESM23_ESM.pdf]

Figure S15

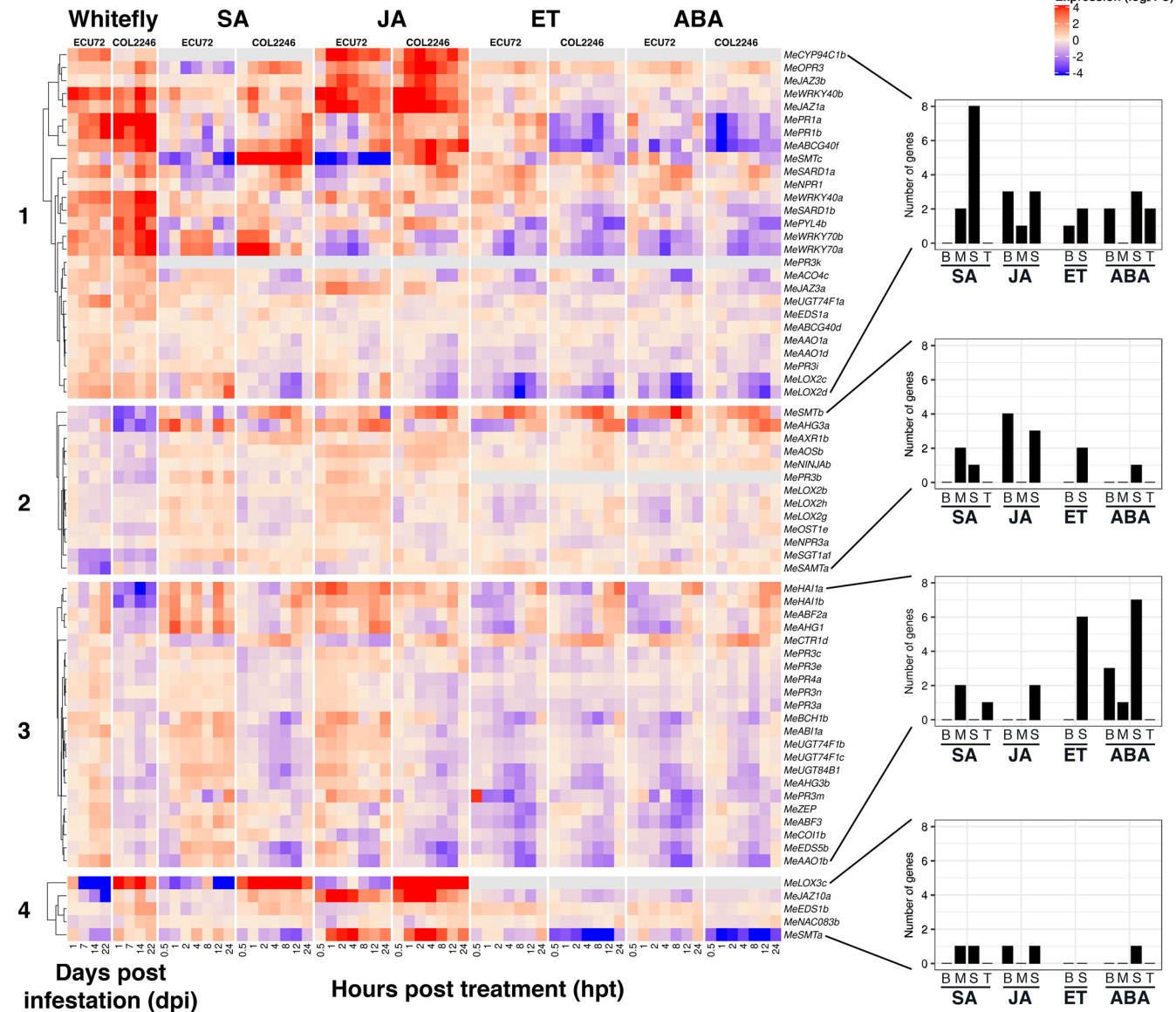

Supplement: Supplementary file 24 — Additional file 24: Figure S15. Cassava hormone-pathway gene expression during whitefly and hormone treatments. Hormone-pathway genes that were also gDEGs during whitefly infestation are displayed as log2FC values during whitefly infestation (1, 7, 14, and 22 dpi) and SA, JA, ET, and ABA treatments (0.5, 1, 2, 4, 8, 12, 24 hpt). Genes are clustered by whitefly expression then hierarchically. The number of gDEGs associated with biosynthesis (B), modification (M), signaling (S), and transport (T) for each hormone is provided on the right of each cluster. Genes that were induced in ECU72 but repressed in COL2246 during infestation (Cluster 3) included five ABA signaling genes (MeAHG1, MeAHG3b, MeABI1a and MeHAI1a and b), two ABA-response genes (MeABF2a and MeABF3), five ET-responsive PR3 chitinase genes, and two SA-modification genes, which convert SA to an inactive form (UGT74F1b-c). [file 12870_2023_4607_MOESM24_ESM.pdf]

Figure S16

GO Term Categories (BP)

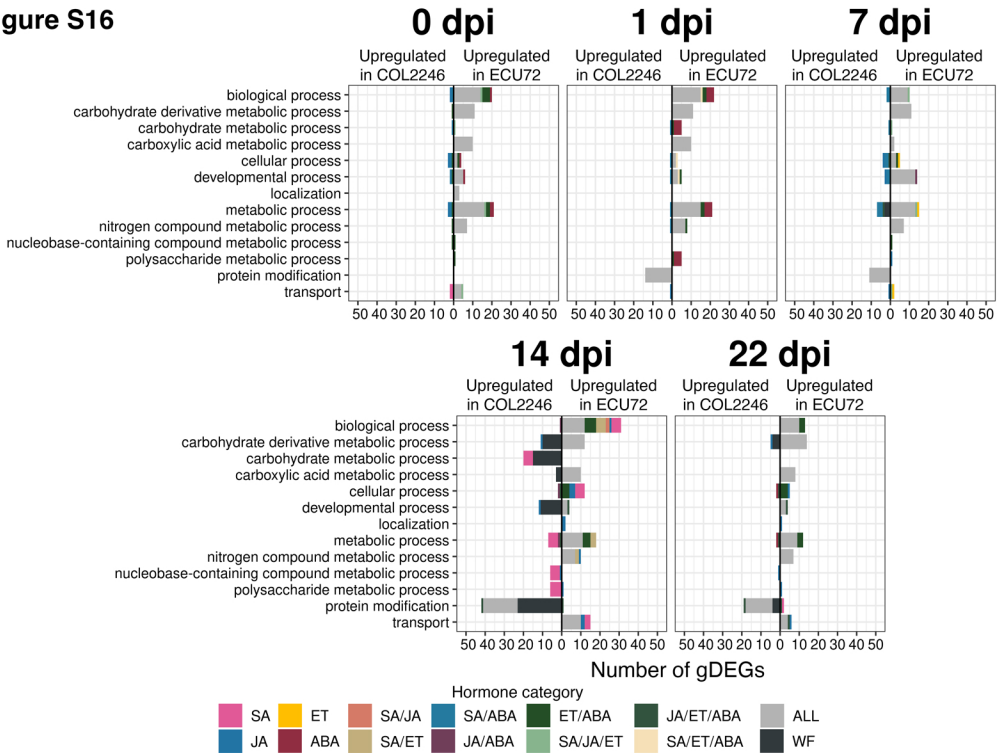

Supplement: Supplementary file 27 — Additional file 27: Figure S16. Functional enrichment of cassava gDEGs in response to whitefly and hormone treatments, non-defense categories. GO-term enrichment was performed on whitefly gDEGs. Numbers of genes enriched for terms not linked to defense are shown. gDEGs responsive to one-three hormones, all hormones, or that are hormone-nonresponsive (WF) are shown. Genes upregulated in ECU72 or COL2246 are displayed on the right and left sides of the x-axis, respectively. Counts and identities of genes within each GO term category are provided in Additional file 25: Table S43 and Additional file 26: Table S45. [file 12870_2023_4607_MOESM27_ESM.pdf]

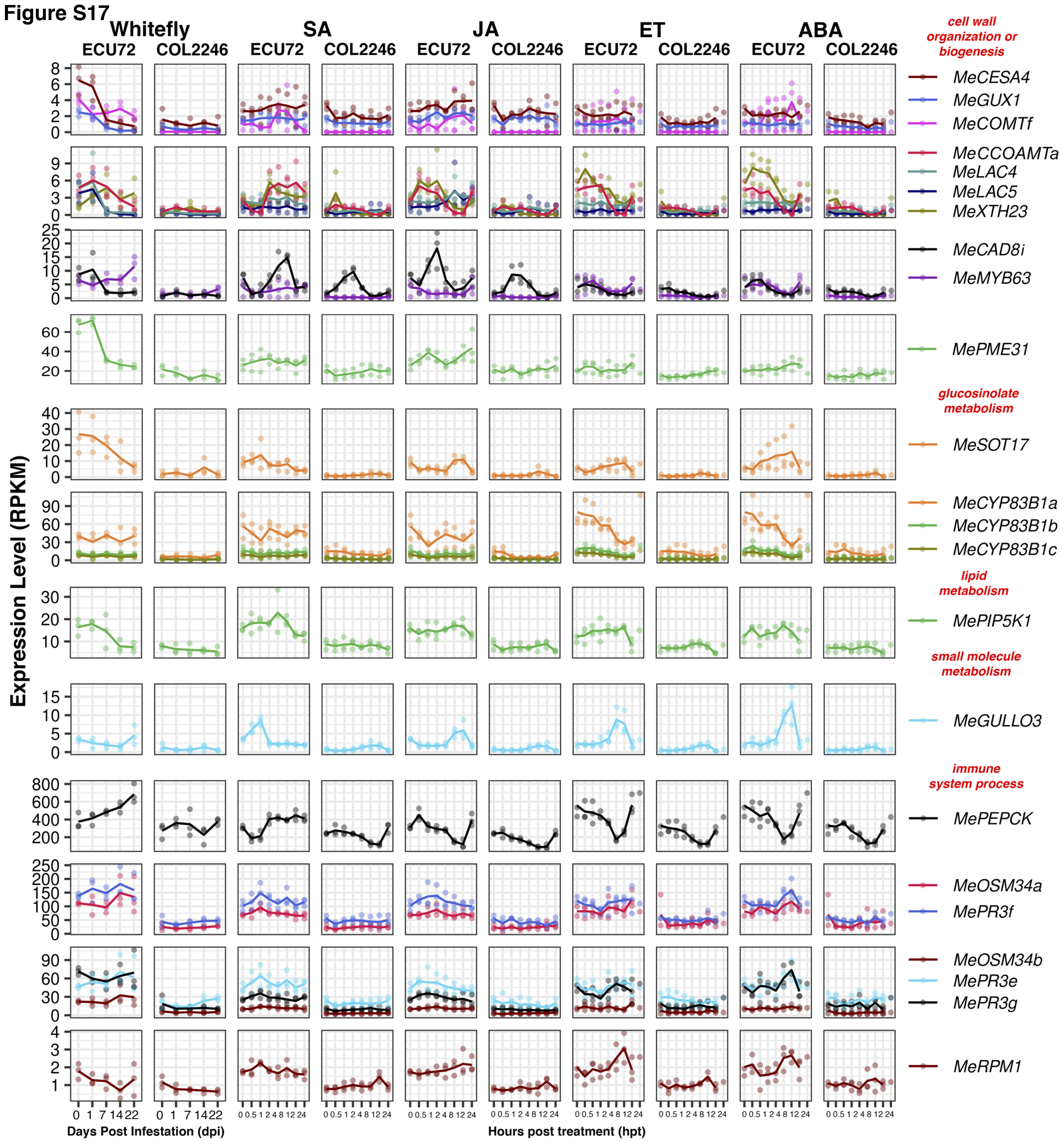

Supplement: Supplementary file 28 — Additional file 28: Figure S17. Expression of whitefly- and hormone-regulated gDEGs enriched in ECU72 versus COL2246 during infestation. The expression of a selection of gDEGs from five enriched GO-term categories (cell wall organization or biogenesis, glucosinolate metabolism, lipid metabolism, small molecule metabolism, and immune system process) from Fig. 5 are shown. Individual values (circles) and average RPKM values (lines) are shown. Among cell-wall-related genes, several lignin biosynthetic genes (MeCOMTf, MeCCOAMTa, MeMYB63, MeLAC4, and MeLAC5) were identified. Genes are grouped based on the magnitude of their response (RPKM values) and denoted in different colors. Gene loci are listed in Additional file 15 or as follows: MeGUX1 (Manes.12G153100), MeCESA4 (Manes.13G038000), MeLAC4 (Manes.08G089800), MeLAC5 (Manes.07G135400), MeXTH23 (Manes.05G108100), MeMYB63 (Manes.06G175200), MePME31 (Manes.02G048600), MeSOT17 (Manes.10G085400), MeCYP83B1a (Manes.05G150100), MeCYP83B1b (Manes.05G139500), MeCYP83B1c (Manes.S058500), MePEPCK (Manes.18G054700), MeOSM34a (Manes.01G064200), MeOSM34b (Manes.01G064300), MeRPM1 (Manes.18G117800), MePIP5K1 (Manes.S096300), MeGULLO3 (Manes.03G061900). [file 12870_2023_4607_MOESM28_ESM.png]

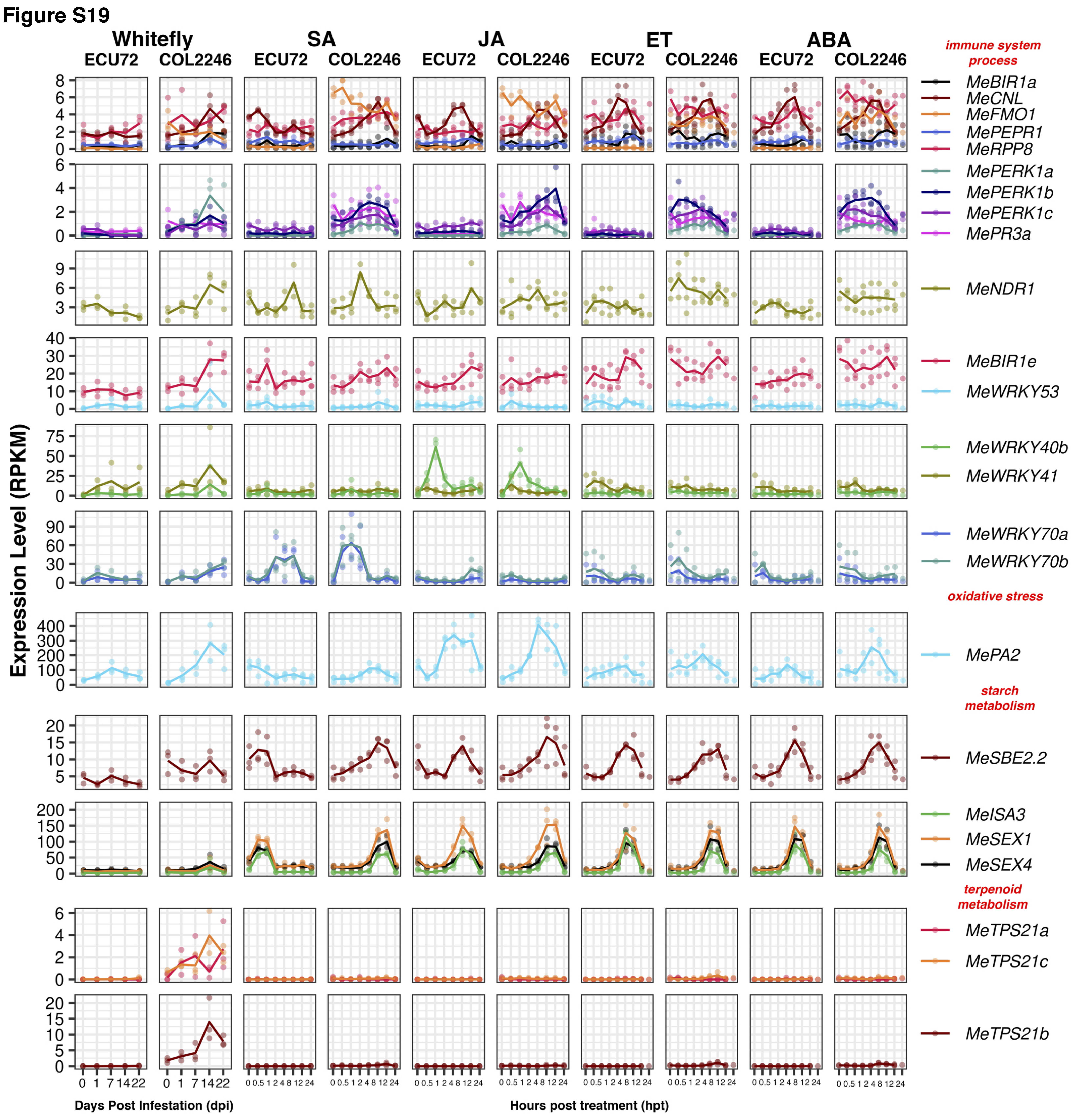

Supplement: Supplementary file 31 — Additional file 31: Figure S19. Expression of whitefly- and hormone-regulated gDEGs enriched in COL2246 versus ECU72 during infestation. The expression of a selection of gDEGs from four enriched GO-term categories (immune system process, oxidative stress, starch metabolism, and terpenoid metabolism) from Fig. 5 are shown. Individual values (circles) and average RPKM values (lines) are shown. Genes are grouped based on the magnitude of their response (RPKM values) and denoted in different colors. Gene loci are listed in Additional file 15 or as follows: MeBIR1a (Manes.01G019000), MeBIR1e (Manes.13G056500), MeFMO1 (Manes.16G091800), MePEPR1 (Manes.16G045200), MeRPP8 (Manes.10G023300), MeCNL (Manes.11G053000), MePERK1a (Manes.11G039800), MePERK1b (Manes.11G041400), MePERK1c (Manes.11G042500), MeNDR1 (Manes.03G123200), MeWRKY41 (Manes.02G011500), MeWRKY53 (Manes.01G047200), MePA2 (Manes.15G104300), MeSBE2.2 (Manes.09G059400), MeISA3 (Manes.18G063500), MeSEX1 (Manes.13G026800), MeSEX4 (Manes.10G053500), MeTPS21a (Manes.02G086100), MeTPS21b (Manes.02G086300), MeTPS21c (Manes.18G101900). [file 12870_2023_4607_MOESM31_ESM.png]

Figure S20

a

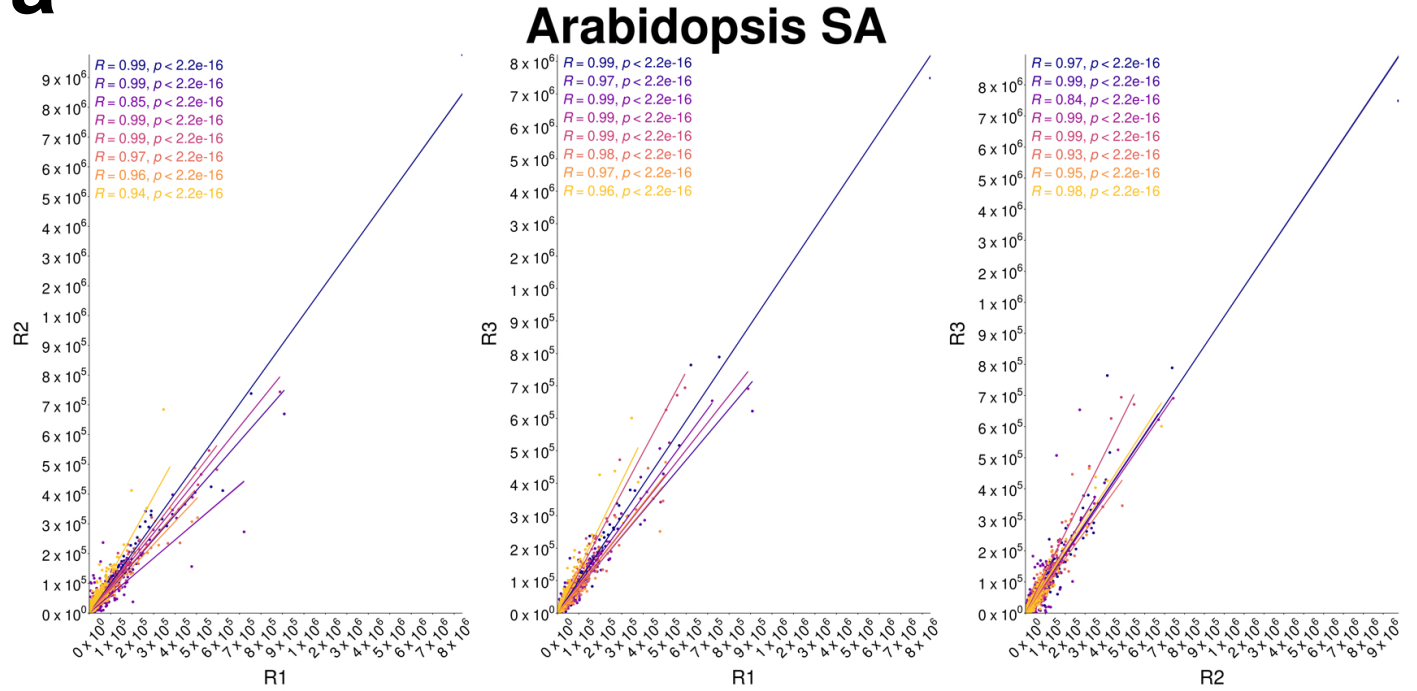

b

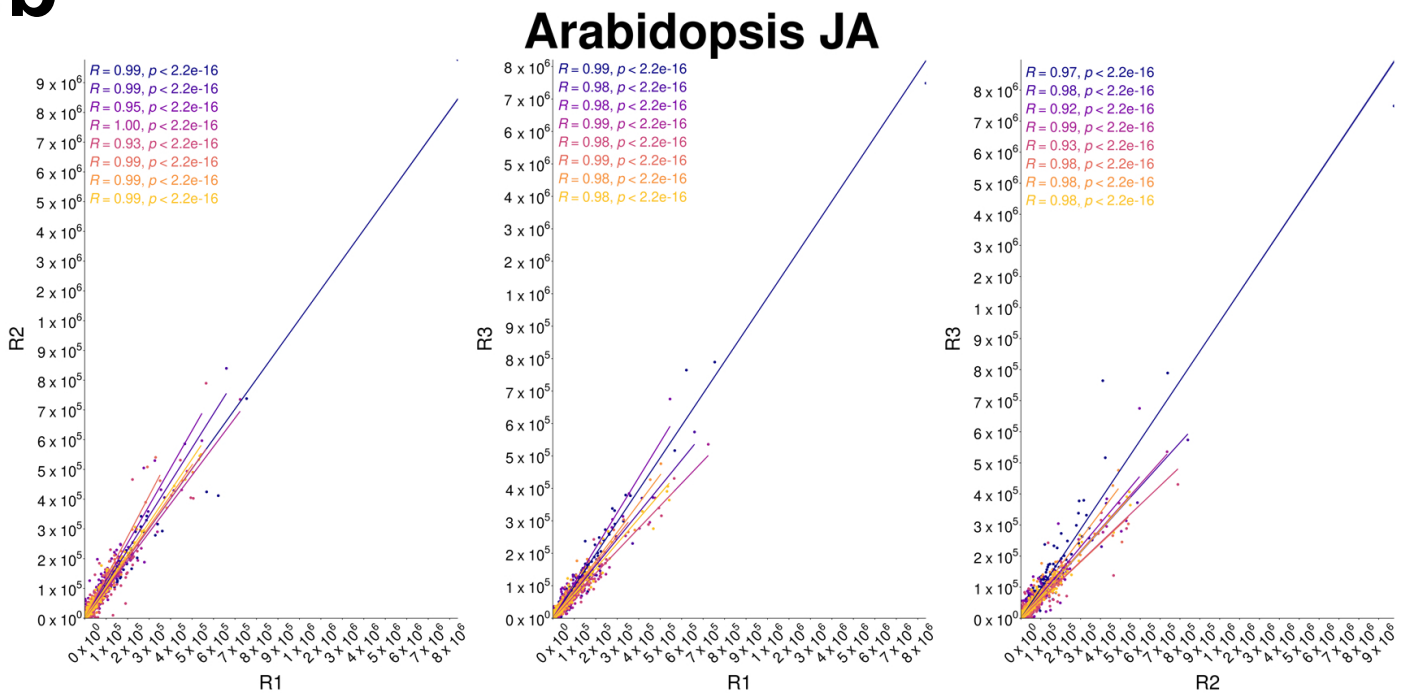

Figure S21

a

ECU72 WF

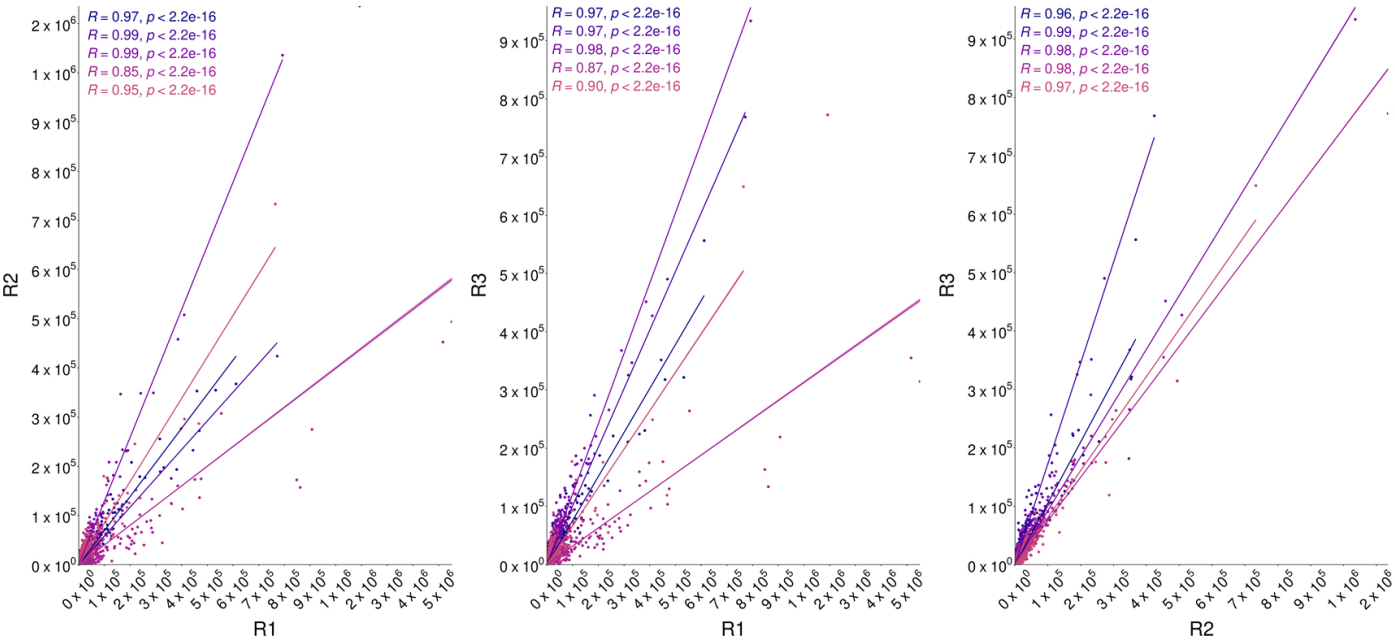

b

COL2246 WF

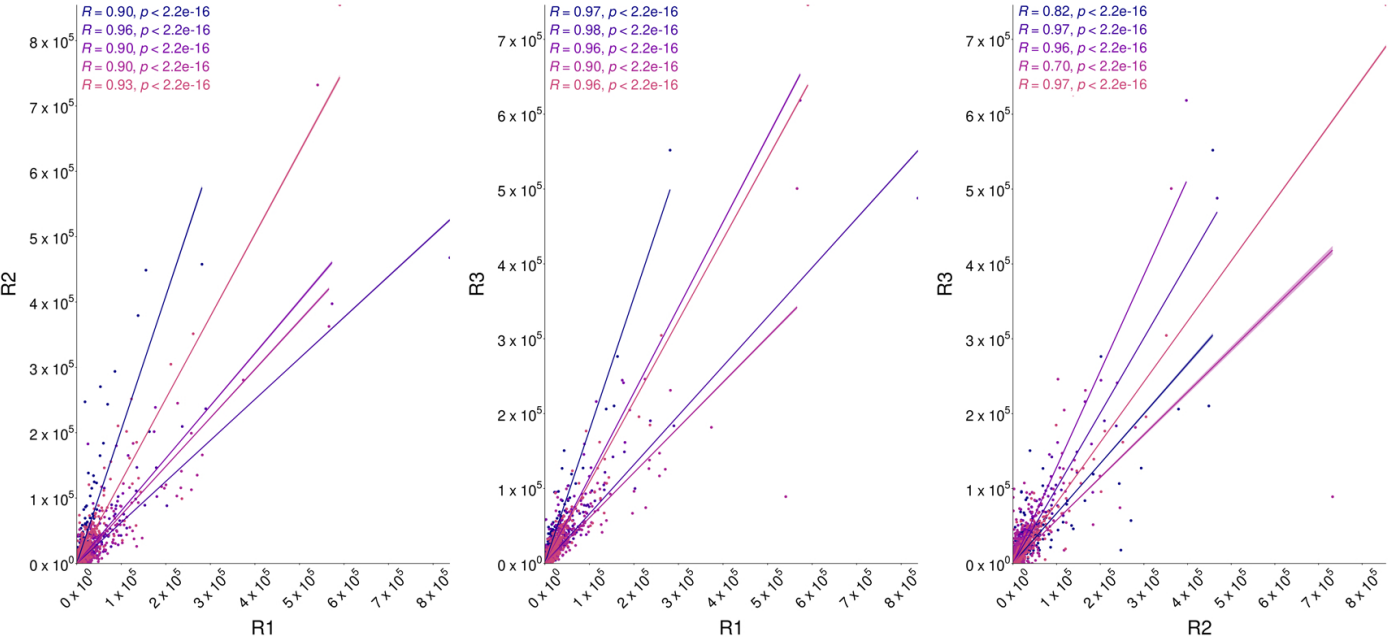

Figure S22

a

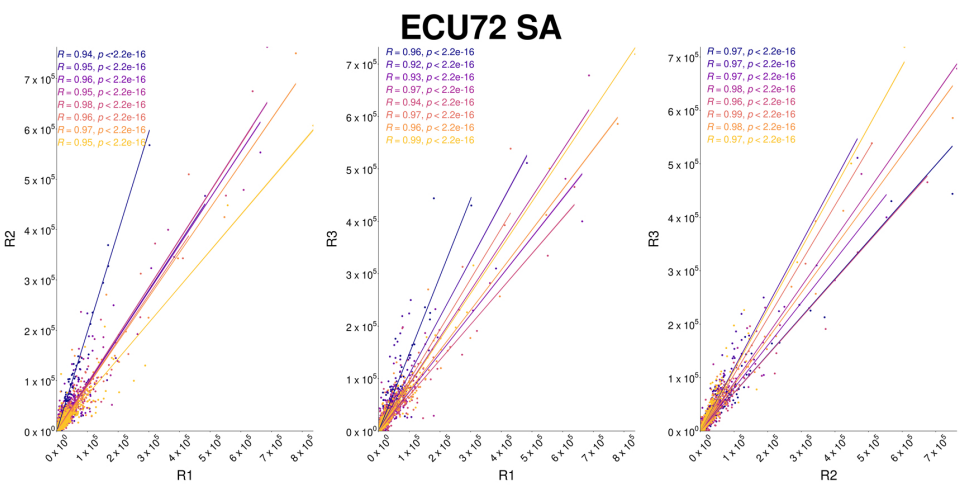

b

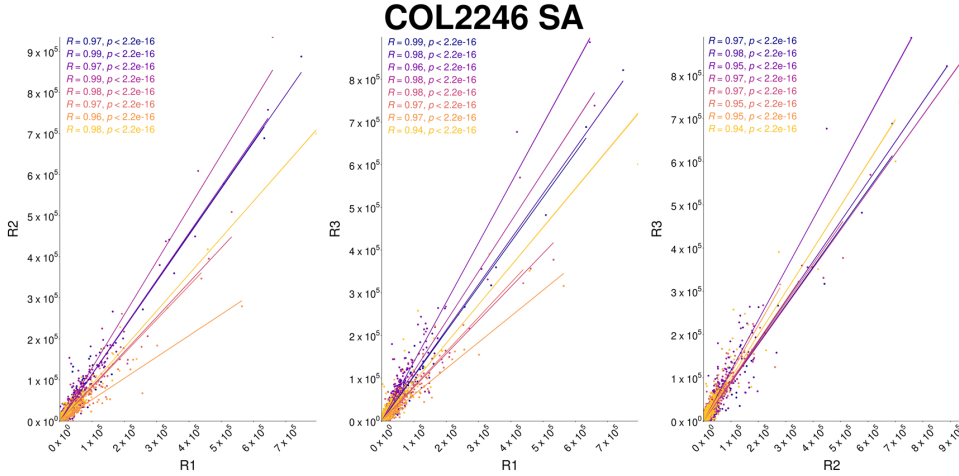

c

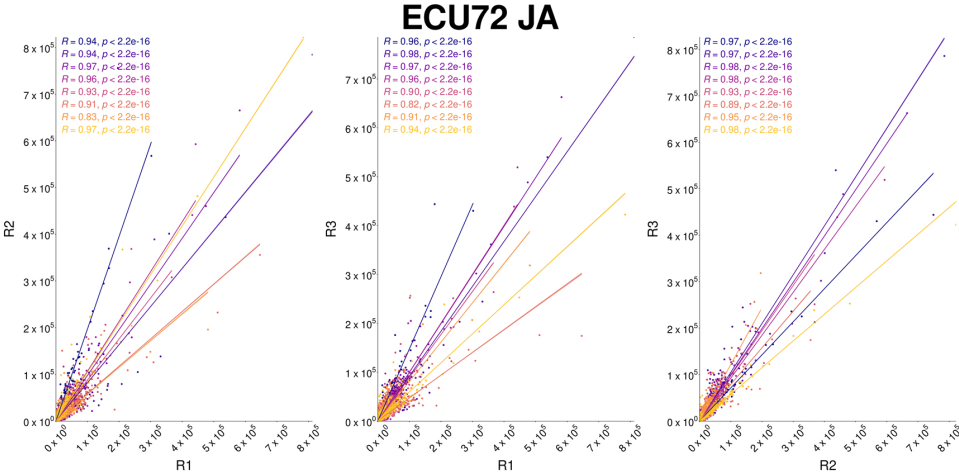

d

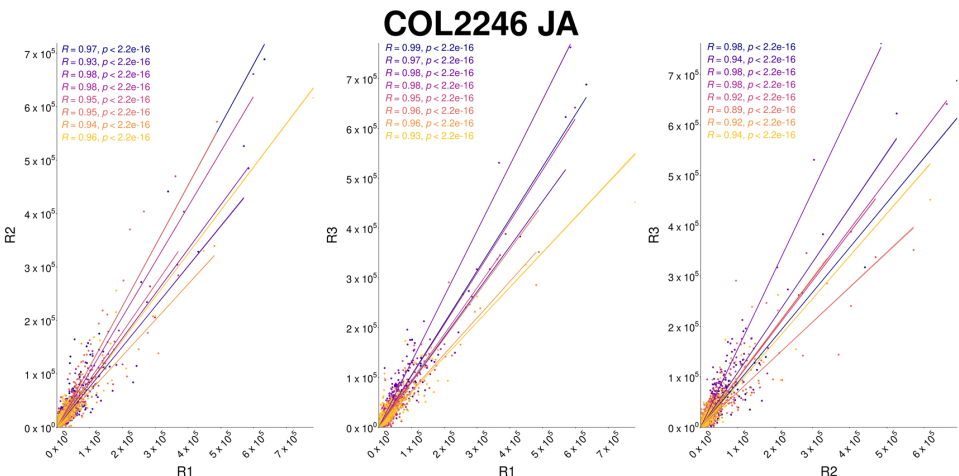

Figure S23

a

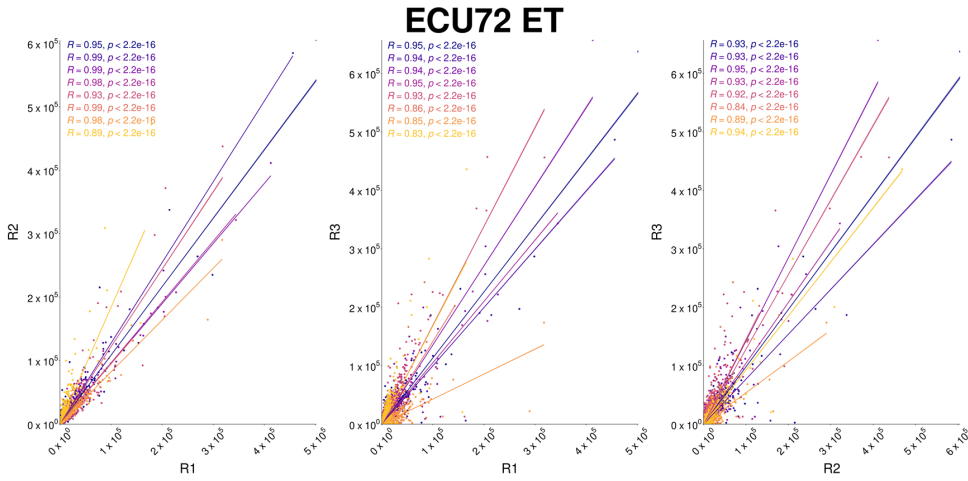

b

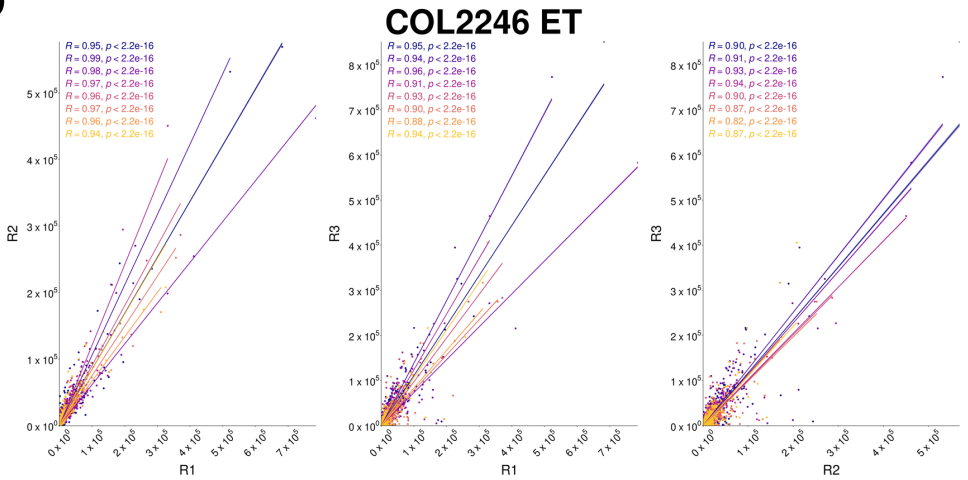

c

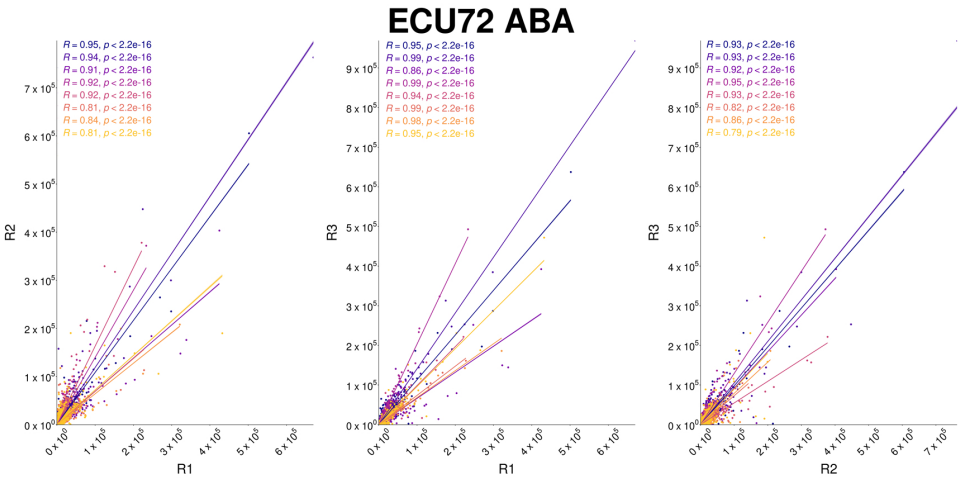

d

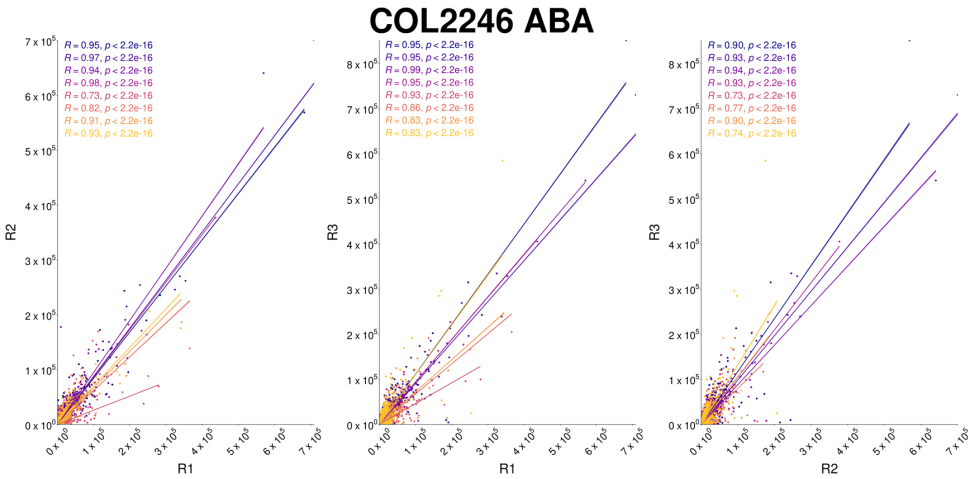

Supplement: Supplementary file 32 — Additional file 32. Replicate correlations for cassava and Arabidopsis treatment samples. Figure S20. Arabidopsis (a) SA and (b) JA treatments. Figure S21. (a) ECU72 and (b) COL2246 whitefly treatments. Figure S22. ECU72 and COL2246 (a,b) SA and (c,d) JA treatments. Figure S23. ECU72 and COL2246 (a,b) ET and (c,d) ABA treatments. Pearson correlation coefficient (R) and p-values between biological replicates (R1-R3) were calculated for genes detected during treatments. Detected genes had an average of 20 reads or more across a hormone-treatment or whitefly infestation time course. Normalized read count values for three biological replicates are shown per time point. RNA-seq read count values for each time point are labeled by color. [file 12870_2023_4607_MOESM32_ESM.pdf]

Figure S24

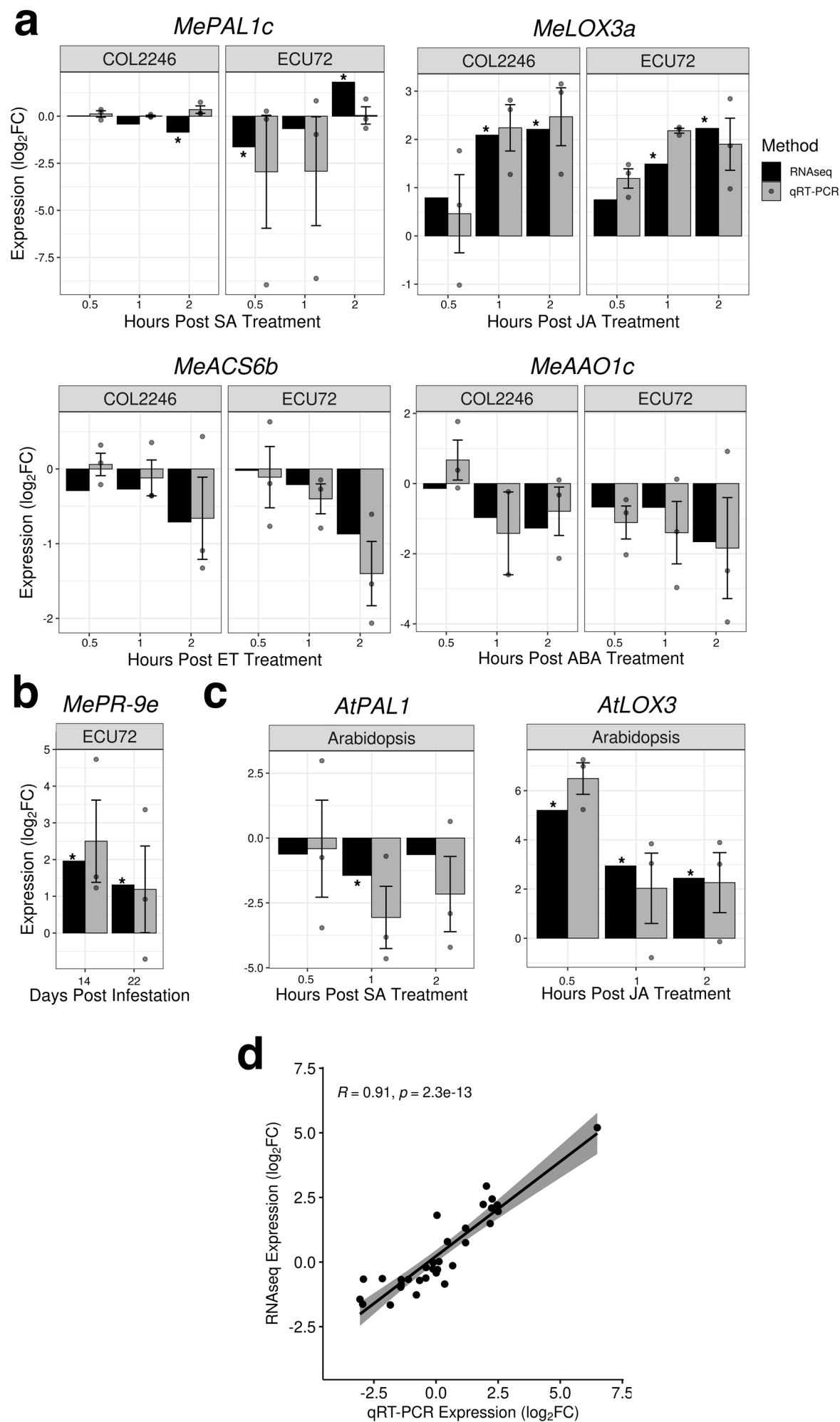

Supplement: Supplementary file 33 — Additional file 33: Figure S24. qRT-PCR validation of RNA-seq expression values. (a) The in silico and qRT-PCR relative expression values of hormone-biosynthetic genes MePAL1c, MeLOX3a, MeACS6b, and MeAAO1c following SA, JA, ET, and ABA treatments of ECU72 and COL2246. qRT-PCR of 0.5, 1 and 2 hpt samples confirmed the RNA-seq data. (b) The in silico and qRT-PCR relative expression values of sentinel PR gene MePR-9e after whitefly infestation in ECU72 at 14 and 22 dpi. Expression of MePR-9e in COL2246 was previously determined [32]. (c) The in silico and qRT-PCR relative expression values of AtPAL1 and AtLOX3 following SA and JA treatments, respectively, were confirmed in Arabidopsis thaliana at 0.5, 1 and 2 hpt in vivo. Bargraphs display qRT-PCR sample values (overlayed points) as well as the average and standard error (SE) of three biological replicates (error-bar graph). For MeAAO1c in COL2246 at 1 hpt (a), only two biological replicates are provided. Primers are provided in Additional file 34. (d) A scatter plot with Pearson correlations demonstrated the relative expression determined by qRT-PCR and RNA-seq showed a strong and significant positive correlation for all biological replicates displayed in panels a-c. All expression values were normalized to MeUBQ in cassava and AtACT7 in Arabidopsis and are relative to the treatment’s 0-h time point. [file 12870_2023_4607_MOESM33_ESM.pdf]
