# Supplementary material for: Integrative transcriptomics reveals association of abscisic acid and lignin pathways with cassava whitefly resistance
Source: BMC Plant Biol. 2023 Dec 20;23:657. doi: 10.1186/s12870-023-04607-y (PMC10731783; doi:10.1186/s12870-023-04607-y)

Figure S3

SA

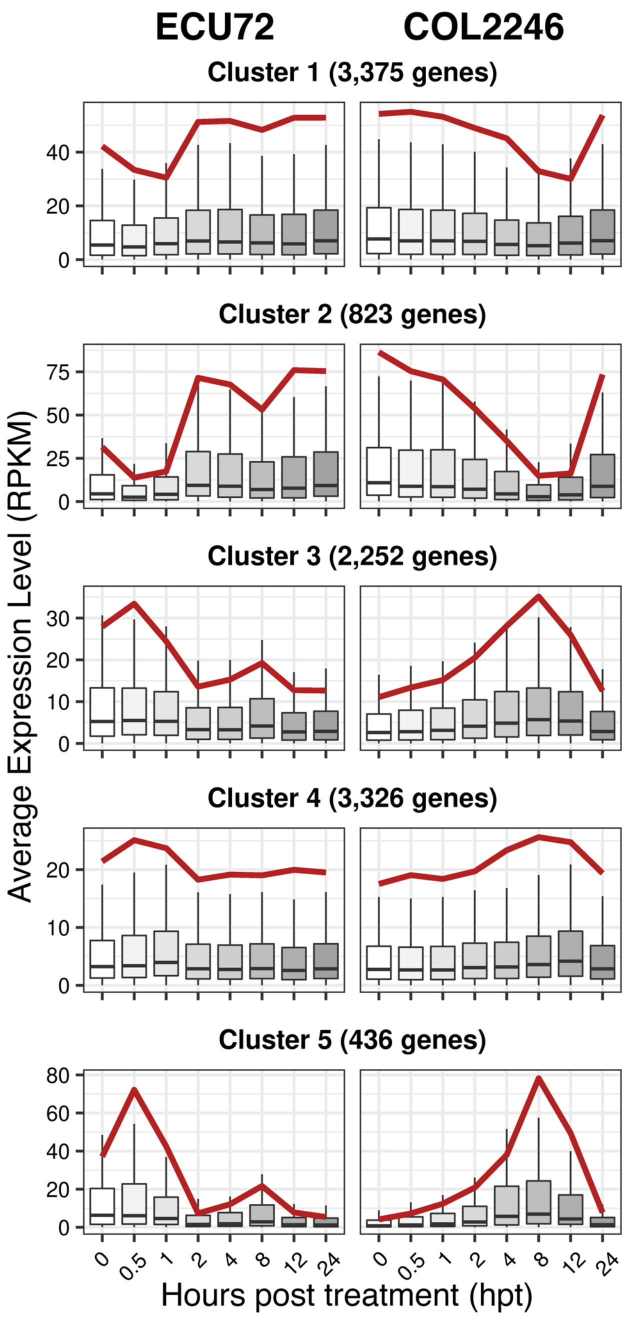

GO Term Categories (BP)

Cluster 1

1. ion transport
2. phenylpropanoid metabolic process
3. response to hormone
4. response to stimulus
5. pigment metabolic process
6. metabolic process
7. developmental process
8. ion homeostasis
9. flavonoid metabolic process
10. secondary metabolic process

Cluster 2

1. ion transport
2. response to stimulus
3. nitrogen compound metabolic process
4. response to hormone
5. lipid metabolic process
6. response to biotic stimulus
7. gibberellin metabolic process
8. transport
9. carboxylic acid metabolic process
10. cell wall organization or biogenesis

Cluster 3

1. nucleobase-containing compound metabolic process

Cluster 4

1. RNA processing
2. immune system process
3. response to hormone

Cluster 5

1. immune system process
2. metabolic process
3. rhythmic process
4. response to stimulus

Figure S4

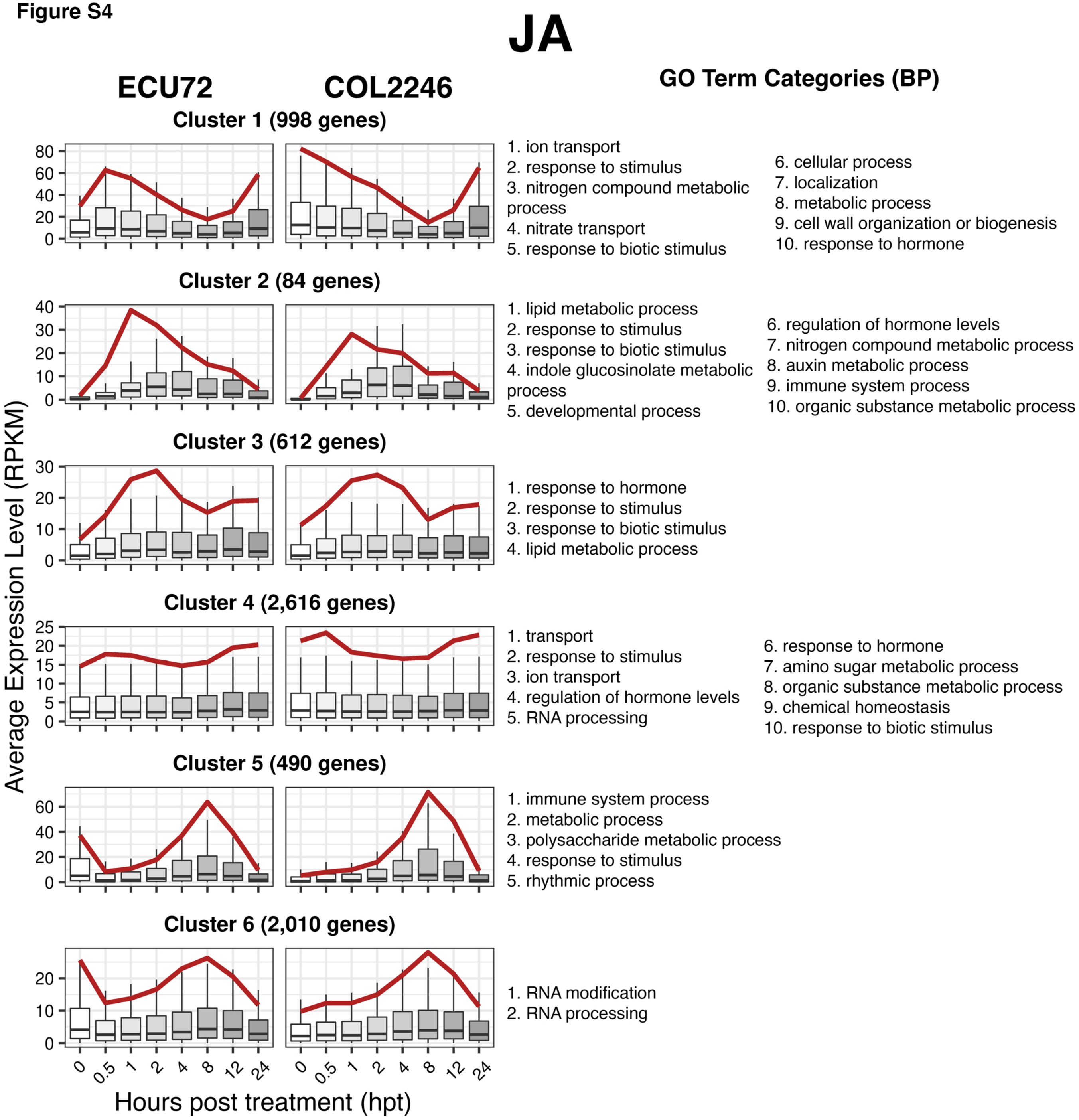
JA
ECU72
COL2246
GO Term Categories (BP)
Cluster 1 (998 genes)
1. ion transport
2. response to stimulus
3. nitrogen compound metabolic process
4. nitrate transport
5. response to biotic stimulus
6. cellular process
7. localization
8. metabolic process
9. cell wall organization or biogenesis
10. response to hormone
Cluster 2 (84 genes)
1. lipid metabolic process
2. response to stimulus
3. response to biotic stimulus
4. indole glucosinolate metabolic process
5. developmental process
6. regulation of hormone levels
7. nitrogen compound metabolic process
8. auxin metabolic process
9. immune system process
10. organic substance metabolic process
Cluster 3 (612 genes)
1. response to hormone
2. response to stimulus
3. response to biotic stimulus
4. lipid metabolic process
Cluster 4 (2,616 genes)
1. transport
2. response to stimulus
3. ion transport
4. regulation of hormone levels
5. RNA processing
6. response to hormone
7. amino sugar metabolic process
8. organic substance metabolic process
9. chemical homeostasis
10. response to biotic stimulus
Cluster 5 (490 genes)
1. immune system process
2. metabolic process
3. polysaccharide metabolic process
4. response to stimulus
5. rhythmic process
Cluster 6 (2,010 genes)
1. RNA modification
2. RNA processing
Average Expression Level (RPKM)
0 0.5 1 2 4 8 12 24
Hours post treatment (hpt)

Figure S5

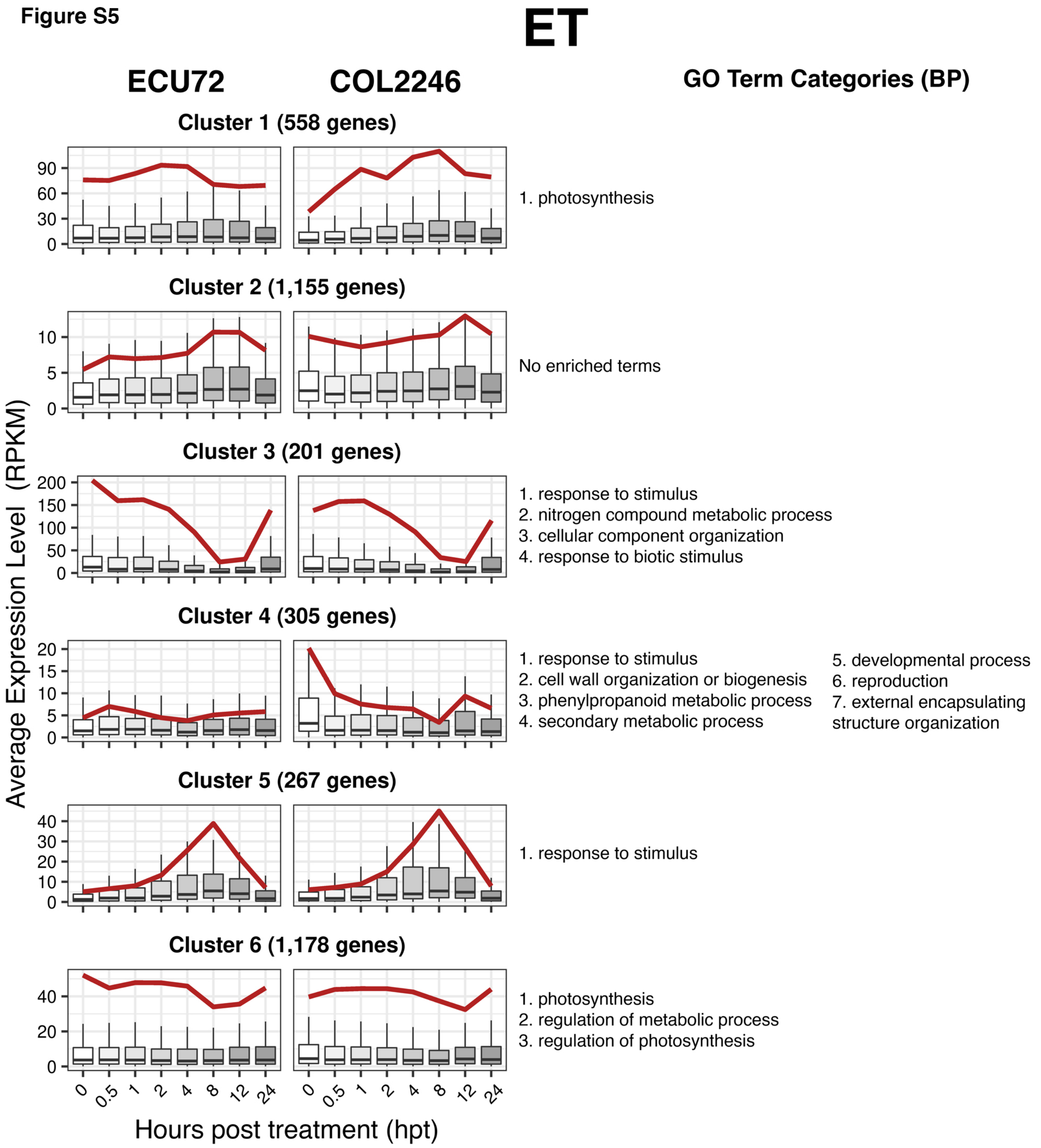
ET
ECU72
COL2246
GO Term Categories (BP)
Cluster 1 (558 genes)
1. photosynthesis
Cluster 2 (1,155 genes)
No enriched terms
Cluster 3 (201 genes)
1. response to stimulus
2. nitrogen compound metabolic process
3. cellular component organization
4. response to biotic stimulus
Cluster 4 (305 genes)
1. response to stimulus
2. cell wall organization or biogenesis
3. phenylpropanoid metabolic process
4. secondary metabolic process
5. developmental process
6. reproduction
7. external encapsulating structure organization
Cluster 5 (267 genes)
1. response to stimulus
Cluster 6 (1,178 genes)
1. photosynthesis
2. regulation of metabolic process
3. regulation of photosynthesis
Average Expression Level (RPKM)
0
0.5
1
2
4
8
12
24
Hours post treatment (hpt)

Figure S6

ABA

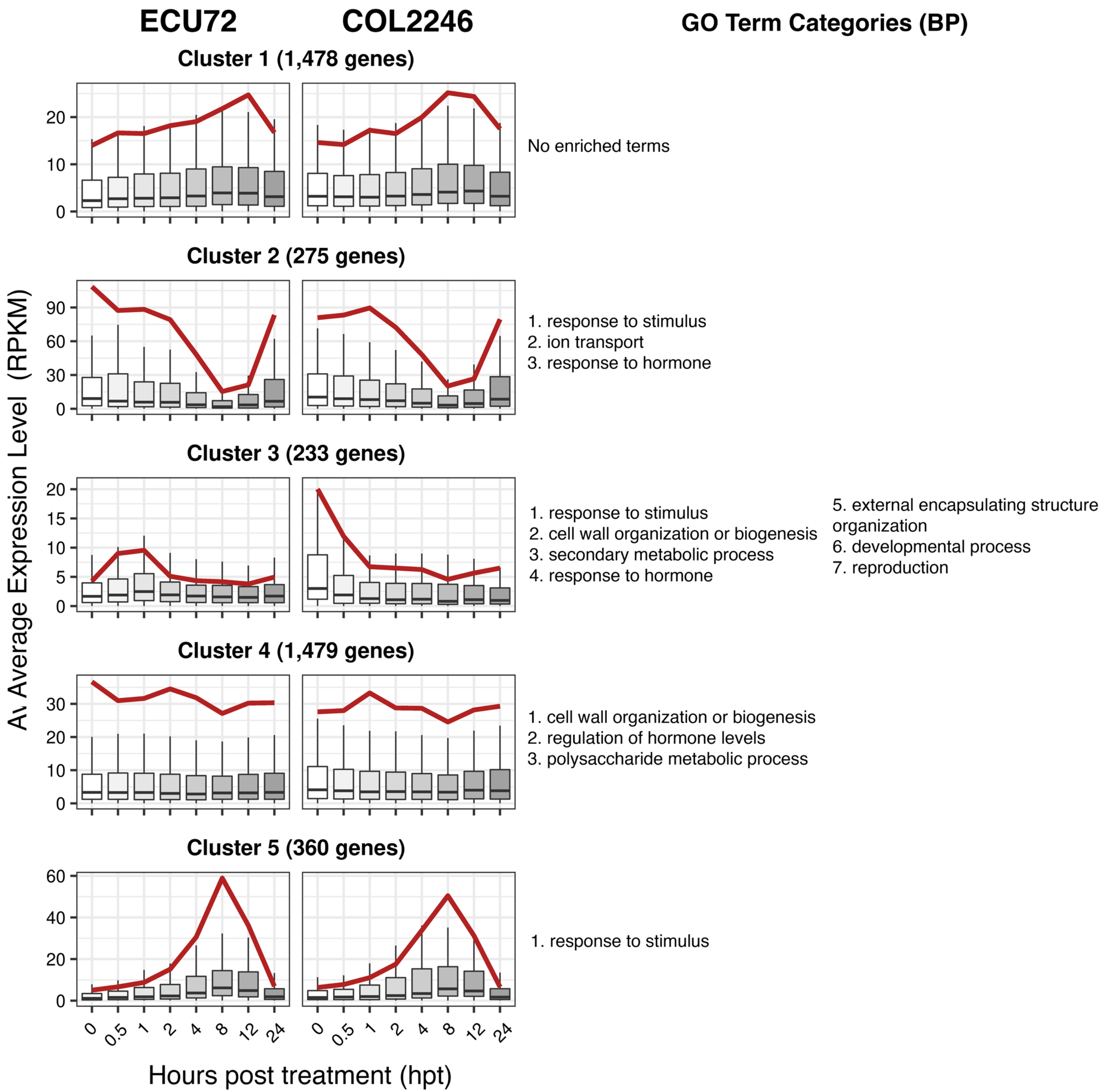

Supplement: Supplementary file 6 — Additional file 6. Clustering and functional enrichment of gDEGs in ECU72 versus COL2246 during hormone treatment displayed as k-means clusters. Figure S3. SA gDEGs. Figure S4. JA gDEGs. Figure S5. ET gDEGs. Fig. S6. ABA gDEGs. SA gDEGs mainly differed due to reciprocal regulation, while reciprocity in JA responses was primarily due to differences in transcript levels at 0 hpt (i.e., Clusters 1, 5 and 6). 71% of gDEGs (4,856 of 6,810 genes) identified in the JA response were gDEGs at 0 h (Additional file 1: Table S10). Differential responses of ECU72 and COL2246 to JA, ET, or ABA was largely attributed to differences in the transcript levels of gDEGs. Categories of significantly enriched (p ≤ 0.05) GO terms ranked by adjusted p-value are provided for each cluster in Additional file 7. gDEGs were identified by comparisons of transcript levels in ECU72 versus COL2246 during SA treatments and had |log2FC| ≥ 1 and FDR ≤ 5%. Boxplot whiskers represent values within 1.5 x IQR, and box values represent the first quartile, median, and third quartile values. Outliers (points beyond whiskers) are not displayed. Lines display average expression values (RPKM) at 0 to 24 hpt. [file 12870_2023_4607_MOESM6_ESM.pdf]
