# Supplementary material for: Integrative transcriptomics reveals association of abscisic acid and lignin pathways with cassava whitefly resistance
Source: BMC Plant Biol. 2023 Dec 20;23:657. doi: 10.1186/s12870-023-04607-y (PMC10731783; doi:10.1186/s12870-023-04607-y)
Supplement: Supplementary file 19 — Additional file 19: Figure S13. Clustering and functional enrichment of genes differentially expressed in ECU72 versus COL2246 during whitefly infestation. Whitefly-responsive gDEGs were grouped into six k-means clusters. For each cluster, enriched GO term categories (p ≤ 0.05) were ranked by adjusted p-value (Additional file 20). Cluster 1 and 2 had similar temporal regulatory programs, while Clusters 3-6 were distinct. Cluster 2 genes were enriched for cell-wall-related processes while all other cluster were enriched for processes related to defense such as response to biotic stimulus, response to stimulus, or immune system process. gDEGs were identified by comparisons of transcript levels in ECU72 versus COL2246 during whitefly infestation and had |log2FC| ≥ 1 and FDR ≤ 5%. Boxplot whiskers represent values within 1.5 x IQR, and box values represent the first quartile, median, and third quartile values. Outliers (points beyond whiskers) are not displayed. Lines display average expression values (RPKM) at 0 to 22 dpi. [file 12870_2023_4607_MOESM19_ESM.pdf]

Figure S13

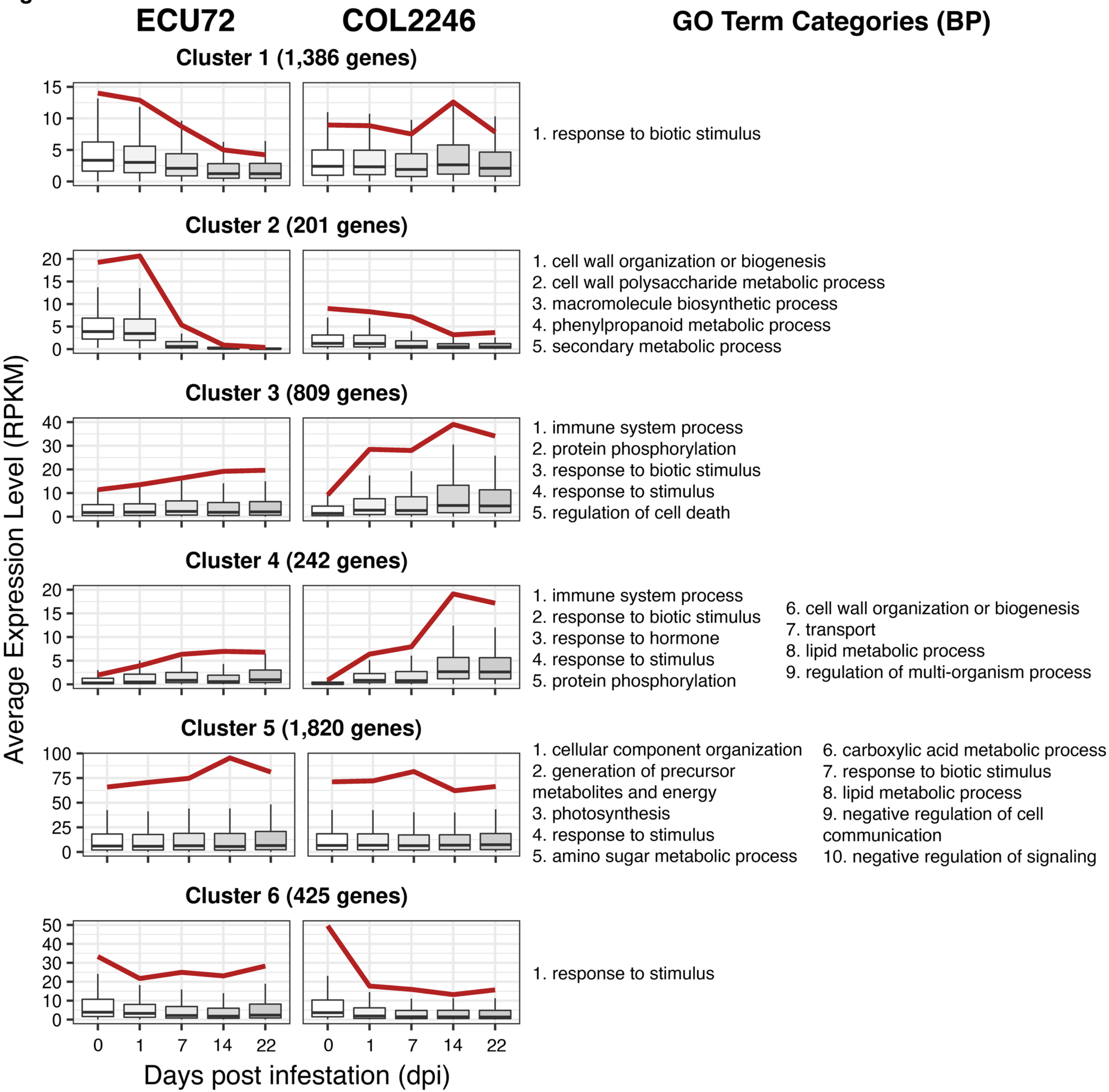
ECU72
COL2246
GO Term Categories (BP)
Cluster 1 (1,386 genes)
1. response to biotic stimulus
Cluster 2 (201 genes)
1. cell wall organization or biogenesis
2. cell wall polysaccharide metabolic process
3. macromolecule biosynthetic process
4. phenylpropanoid metabolic process
5. secondary metabolic process
Cluster 3 (809 genes)
1. immune system process
2. protein phosphorylation
3. response to biotic stimulus
4. response to stimulus
5. regulation of cell death
Cluster 4 (242 genes)
1. immune system process
2. response to biotic stimulus
3. response to hormone
4. response to stimulus
5. protein phosphorylation
6. cell wall organization or biogenesis
7. transport
8. lipid metabolic process
9. regulation of multi-organism process
Cluster 5 (1,820 genes)
1. cellular component organization
2. generation of precursor metabolites and energy
3. photosynthesis
4. response to stimulus
5. amino sugar metabolic process
6. carboxylic acid metabolic process
7. response to biotic stimulus
8. lipid metabolic process
9. negative regulation of cell communication
10. negative regulation of signaling
Cluster 6 (425 genes)
1. response to stimulus
Average Expression Level (RPKM)
0
1
7
14
22
Days post infestation (dpi)
