# Supplementary material for: Integrative transcriptomics reveals association of abscisic acid and lignin pathways with cassava whitefly resistance
Source: BMC Plant Biol. 2023 Dec 20;23:657. doi: 10.1186/s12870-023-04607-y (PMC10731783; doi:10.1186/s12870-023-04607-y)

Figure S18

a

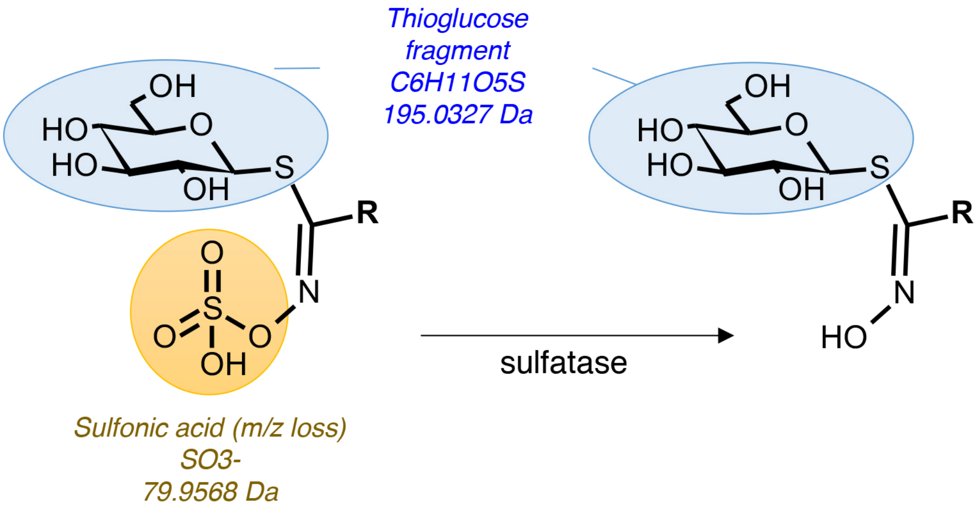

b

Measurement	Glucotropaeolin (benzyl-derivative)	Sinigrin (allyl-derivative)
**RT intact**	2.96 min	0.9 min
**RT desulfonated**	3.72 min	1.3 min
**Molecular Formula intact**	C14H19NO9S2	C10H17NO9S2
**Molecular Formula desulfonated**	C14H19NO6S (Δ: SO3)	C10H17NO6S (Δ: SO3)
**Measured m/z intact [M-H]-**	408.048	358.0272
**Measured m/z desulfonated [M-H]-**	328.0878 (**Δ:79.96**)	278.0672 (**Δ:79.96**)
**MS spectrum (desulfonated)**	657.1811 = 2M-H]-<br>166.03449 = M-gluc<br>**195.0351 = thioglucose**	557.1495 = 2M-H]-<br>116.0187 = M-gluc<br>**195.0351 = thioglucose**

c

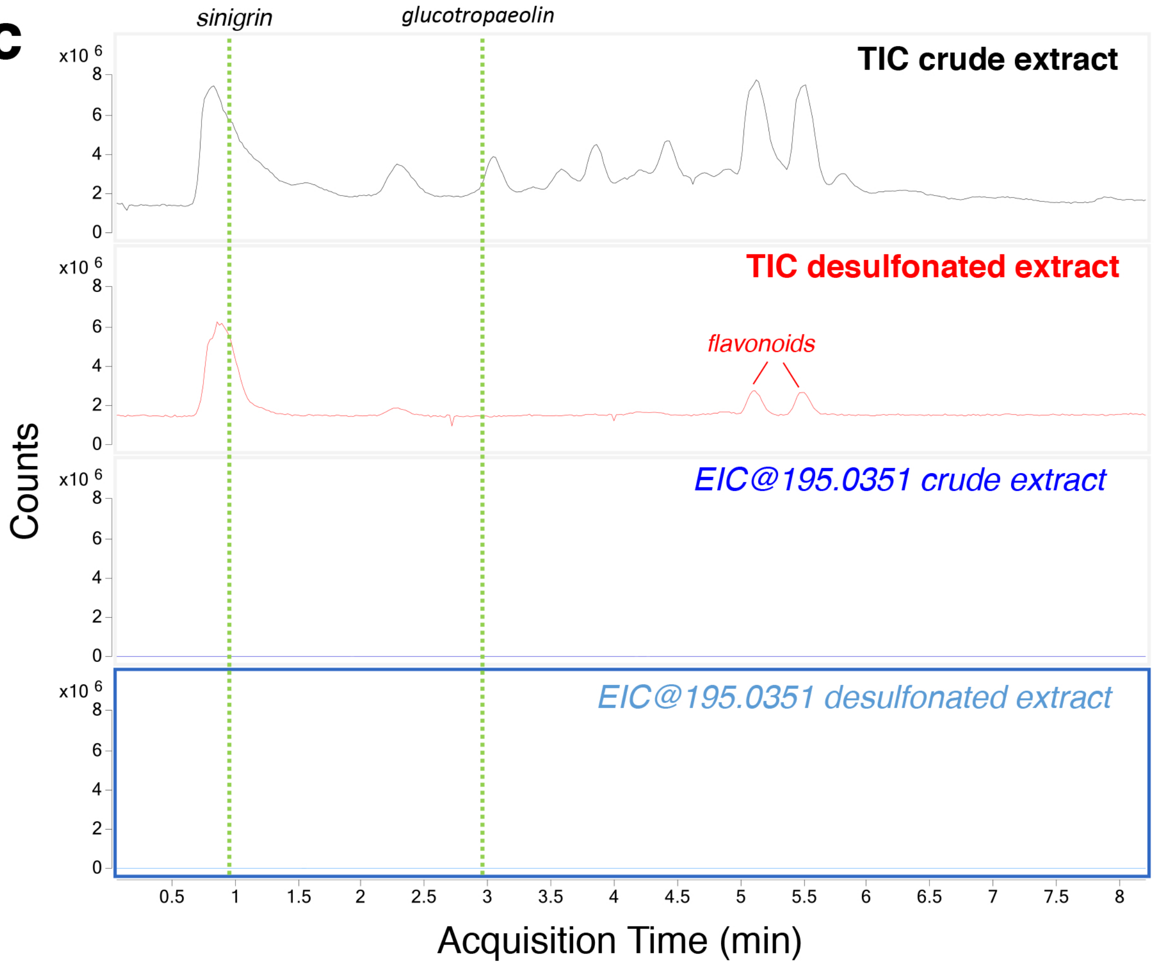

Supplement: Supplementary file 29 — Additional file 29: Figure S18. Analysis of glucosinolates in cassava leaves. (a) Scheme of sulfatase reaction and its effect on glucosinolate structure. (b) LC-MS measurements of glucosinolates standard solutions treated with sulfatase. (c) Total ion chromatograms (TIC) of leaf extracts before (crude) and after (desulfonated) the sulfatase reaction and extracted ion chromatogram (EIC) of thioglucose fragment, as indicator of presence of glucosinolated structures. [file 12870_2023_4607_MOESM29_ESM.pdf]
